# Clinical Benefits and Utility of Pretherapeutic *DPYD* and *UGT1A1* Testing in Gastrointestinal Cancer: A Secondary Analysis of the PREPARE Randomized Clinical Trial

**DOI:** 10.1001/jamanetworkopen.2024.49441

**Published:** 2024-12-06

**Authors:** Rossana Roncato, Alessia Bignucolo, Elena Peruzzi, Marcella Montico, Elena De Mattia, Luisa Foltran, Michela Guardascione, Mario D’Andrea, Adolfo Favaretto, Fabio Puglisi, Jesse Joachim Swen, Henk-Jan Guchelaar, Giuseppe Toffoli, Erika Cecchin

**Affiliations:** 1 Experimental and Clinical Pharmacology, Centro di Riferimento Oncologico di Aviano (CRO) IRCCS, Aviano, Italy; 2 Department of Medicine, University of Udine, Udine, Italy; 3 Clinical Trial Office, Scientific Direction, Centro di Riferimento Oncologico di Aviano (CRO), IRCCS, Aviano, Italy; 4 Department of Medical Oncology, Centro di Riferimento Oncologico di Aviano (CRO), IRCCS, Aviano, Italy; 5 Department of Medical Oncology, Ospedale San Paolo / Ospedale Padre Pio, Civitavecchia, Rome, Italy; 6 Department of Medical Oncology, Azienda ULSS 2 Marca Trevigiana Distretto di Treviso, Treviso, Italy; 7 Department of Clinical Pharmacy and Toxicology, Leiden University Medical Center, Leiden, the Netherlands

## Abstract

**Importance:**

To date, the clinical benefit and utility of implementing a *DPYD/UGT1A1* pharmacogenetic-informed therapy with fluoropyrimidines and/or irinotecan have not been prospectively investigated.

**Objective:**

To examine clinically relevant toxic effects, hospitalizations, and related costs while preserving treatment intensity and efficacy outcomes in patients with gastrointestinal cancer.

**Design, Setting, and Participants:**

This nonprespecified secondary analysis stems from Pre-Emptive Pharmacogenomic Testing for Preventing Adverse Drug Reactions (PREPARE), a multicenter, controlled, open, block-randomized, crossover implementation trial conducted from March 7, 2017, to June 30, 2020, and includes data from Italy according to a sequential study design. The study population included 563 patients (intervention, 252; control [standard of care], 311) with gastrointestinal cancer (age ≥18 years) who were eligible for fluoropyrimidine and/or irinotecan treatment. Data analysis for the present study was performed from May 27 to October 10, 2024.

**Interventions:**

Participants with actionable variants (DPYD*2A, DPYD*13, .*DPYD* c.2846A>T, and *DPYD* c.1236G>A for fluoropyrimidines, and UGT1A1*28, UGT1A1*6, and UGT1A1*27 for irinotecan) received drug or dose adjustments based on Dutch Pharmacogenetics Working Group recommendations.

**Main Outcomes and Measures:**

The primary outcome was clinically relevant toxic effects (National Cancer Institute Common Terminology Criteria for Adverse Events grade ≥4 hematologic, grade ≥3 nonhematologic, or causing hospitalization, fluoropyrimidines and/or irinotecan causally related). Secondary outcomes included hospitalization rates, toxic effect management costs, intensity of treatment, quality-adjusted life-years, and 3-year overall survival.

**Results:**

Overall, 1232 patients were enrolled in Italy, with 563 included in this analysis (317 [56.3%] men; median age, 68.0 [IQR, 60.0-75.0] years). In the intervention arm, carriers of any actionable genotype exhibited a 90% lower risk of clinically relevant toxic effects compared with the control arm (odds ratio, 0.1; 95% CI, 0.0-0.8; *P* = .04). They also presented higher toxic effect management costs per patient ($4159; 95% CI, $1510-$6810) compared with patients in the intervention arm ($26; 95% CI, 0-$312) (*P* = .004) and a higher rate of hospitalization (34.8% vs 11.8%; *P* = .12). The differences were not significant among all patients. Three-year overall survival did not differ significantly between arms, while quality-adjusted life-years significantly improved in the intervention arm. The pharmacogenetics-informed approach did not manifest a detrimental effect on treatment intensity in actionable genotype carriers.

**Conclusions and Relevance:**

In this secondary analysis of PREPARE, pretreatment application of *DPYD*- and *UGT1A1*-guided treatment appeared to increase safety and reduce hospitalizations and related costs in patients with gastrointestinal cancer. Clinical benefit did not appear to be affected.

**Trial Registration:**

ClinicalTrials.gov Identifier: NCT03093818

## Key Points

**Question:**

What is the clinical and utility impact of pharmacogenetic-informed prescribing of fluoropyrimidines and/or irinotecan based on *DPYD*/*UGT1A1* panel analysis in patients with gastrointestinal cancer compared with standard of care?

**Findings:**

In this secondary analysis of 563 patients in the PREPARE trial, carriers of any actionable genotype in the intervention arm showed a significant 90% decrease in clinically relevant toxic effects, reduced number of hospitalizations, and lower toxic effect management cost per patient. No significant decrease in the intensity of treatment and 3-year overall survival was observed.

**Meaning:**

In this trial, pharmacogenetic-informed prescribing increased safety and did not appear to compromise efficacy in patients with gastrointestinal cancer.

## Teaser

This secondary analysis of a randomized clinical trial examines the use of pharmacogenetic-informed prescribing of fluoropyrimidines and/or irinotecan in patients with gastrointestinal cancer.

## Introduction

Fluoropyrimidines are a class of antimetabolite drugs used for the treatment of solid tumors, including gastrointestinal (GI) tract malignant tumors.^1^ Combining fluoropyrimidines with irinotecan has been shown to improve treatment outcomes, especially in advanced stages of disease.^2,3^ Although fluoropyrimidine-based regimens are generally well tolerated, adverse drug reactions (ADRs) can be severe in 20% to 30% of patients. Combination with irinotecan carries an additional risk of toxic effects, with complex regimens such as folinic acid, fluorouracil, and irinotecan often being administered in combination with biologic agents (eg, cetuximab or bevacizumab)^4,5^ or oxaliplatin.^6^ Genetic variants of *DPYD* resulting in dihydropyrimidine dehydrogenase (DPD) deficiency have been associated with the risk of developing severe fluoropyrimidine-related toxic effects in randomized clinical trials,^7,8,9^ meta-analyses,^10^ and prospective studies.^11,12,13^ In addition, defective *UGT1A1* activity has been associated with ADRs, such as neutropenia and diarrhea, after irinotecan treatment.^14,15^ Pharmacogenetic guidelines are available for both fluoropyrimidines (eg, fluorouracil, capecitabine, and tegafur) and irinotecan.^16,17,18^

Members of our group and others have previously highlighted the association between *DPYD* and *UGT1A1* genotypes and toxic effect–related patient costs,^19,20,21^ as well as the cost-effectiveness of a genotype-based dosing approach in large patient cohorts.^22^ However, despite approximately 40% of patients receiving a first fluoropyrimidine prescription for GI cancer will receive further treatment, including irinotecan, at disease progression,^23^ to our knowledge, no study has examined the clinical benefit and utility of a combined *DPYD/UGT1A1* panel analysis.

Since 2020, the European Medicines Agency has recommended testing for DPD deficiency before fluoropyrimidine-based treatment to adjust the starting dose.^24^ Variants DPYD*2A (rs3918290), DPYD*13 (rs55886062), *DPYD* c.2846A>T (rs67376798, D949V), and *DPYD* c.1236G>A (rs56038477, tagging *DPYD*-HapB3), which cause decreased or absent DPD enzyme function, are recommended for pretreatment analysis.^16,17^ Similar recommendations exist for the gene-drug interaction between *UGT1A1* and irinotecan, focusing on UGT1A1*28 and UGT1A1*6, associated with decreased protein function and increased toxic effect risk.^18^ However, their clinical application is debated^25^ due to major concerns related to possible impairment of drug exposure in patients undergoing first-line dose reduction, which in turn could affect treatment success.^26^

In the present study, a secondary analysis of the multicenter, controlled, open, block-randomized Pre-Emptive Pharmacogenomic Testing for Preventing Adverse Drug Reactions (PREPARE)-Ubiquitous Pharmacogenomics trial,^27,28^ was performed. The PREPARE protocol is available in Supplement 1. The aim was to assess the clinical benefits and utility of *DPYD/UGT1A1* pharmacogenetic panel–informed dose individualization in participants in the Italian PREPARE cohort receiving fluoropyrimidine and/or irinotecan treatment for GI cancer.

## Methods

### Study Population

**Figure 1.**
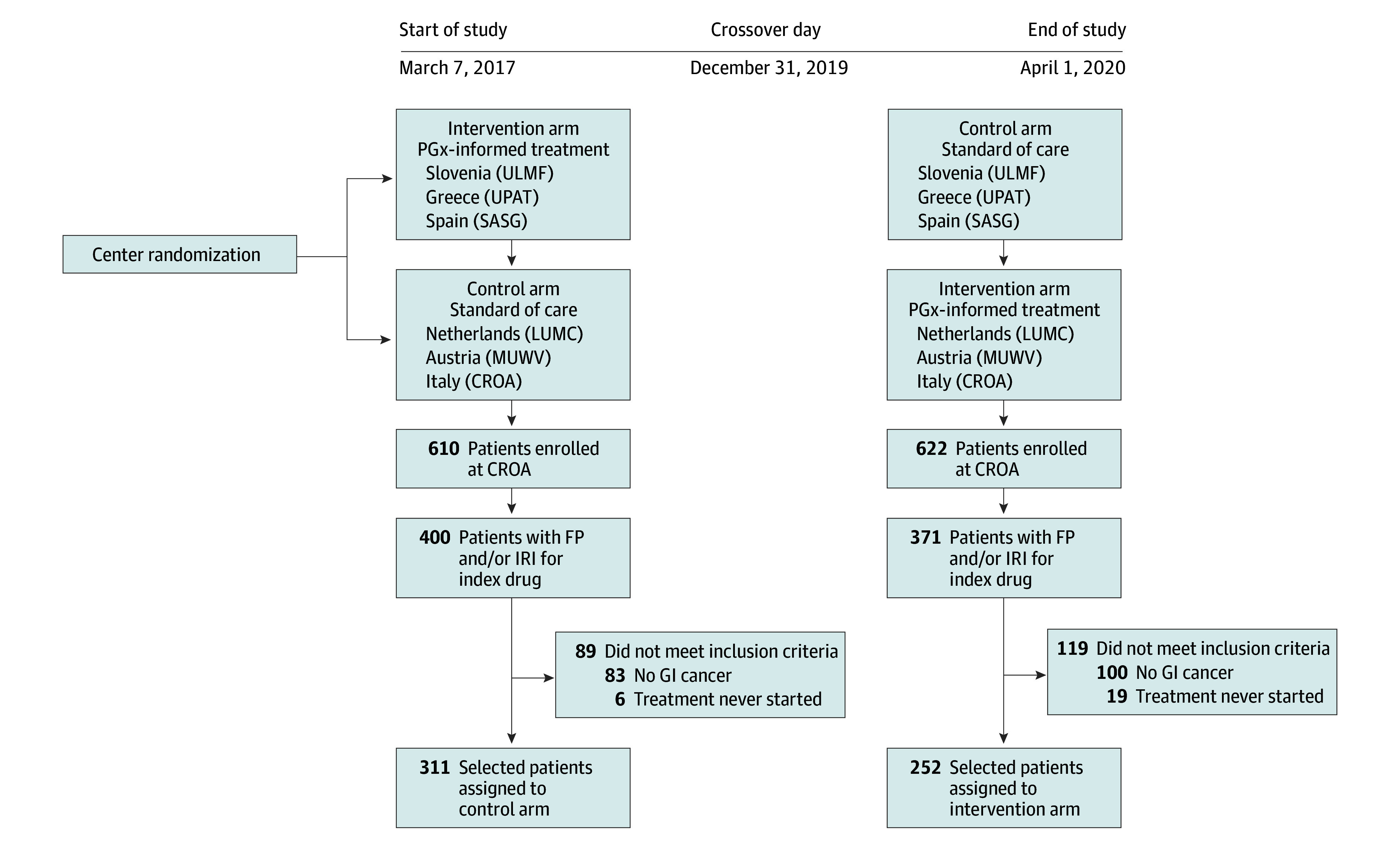
Patient Flow and Study Design CROA indicates Centro di Riferimento Oncologico di Aviano; FP, fluoropyrimidine; GI, gastrointestinal; IRI, irinotecan; LUMC, Leiden University Medical Center; MUWV, Medical University of Vienna; PGx, pharmacogenetics; SASG, Servicio Andaluz de Salud; ULMF, University of Ljubljana; and UPAT, University of Patras.

Inclusion criteria for this single-country nonprespecified secondary analysis were enrollment in the PREPARE trial (between March 7, 2017, and June 30, 2020) at 1 of the 3 Italian clinical centers (eMethods in Supplement 2), use of fluoropyrimidines (capecitabine or systemic fluorouracil) or irinotecan as the index drug, availability of *DPYD* and *UGT1A1* genotype data, and a confirmed histologic diagnosis of GI cancer (Figure 1). The analysis focused on patients with GI cancer due to their high likelihood of receiving fluoropyrimidines and/or irinotecan throughout their treatment. Approval was granted by the Ubiquitous Pharmacogenomics Consortium Executive Board. All participants provided informed consent for participation in the PREPARE trial and for additional pharmacogenetic research after completion of PREPARE. Participants did not receive any financial compensation. The Consolidated Standards of Reporting Trials (CONSORT) reporting guideline was followed.^29^

According to the PREPARE protocol, patients were asked to self-report their ethnicity at the time of enrollment, according to 6 predetermined categories (Caucasian, African, East/Southeast Asian, West Asian, Hispanic, or other). The variable was collected because the minor allele frequency of the pharmacogenetic variants included in the PREPARE panel may vary by ethnicity, potentially affecting study outcome.

### Genotyping and Intervention

As part of central study procedures, a blood or saliva sample was collected for genomic DNA extraction prior to initiating the index drug for all participants. For this secondary analysis, the patient status for the following polymorphisms was considered: DPYD*2A (rs3918290), DPYD*13 (rs55886062), *DPYD* c.2846A>T (rs67376798, D949V), *DPYD* c.1236G>A (rs56038477, tagging HapB3), UGT1A1*28/*37 (rs8175347), UGT1A1*6 (rs4148323), and UGT1A1*27 (rs35350960).

Patients treated with fluoropyrimidines and carrying at least 1 *DPYD* variant or treated with irinotecan and carrying at least 2 *UGT1A1* variants were defined as having an actionable genotype. Recommended dose changes for actionable genotype carriers in the intervention arm were based on Dutch Pharmacogenetics Working Group guidelines^30^ (Table 1) and conveyed to physicians through a pharmacogenetics report before treatment initiation. Adherence to clinical recommendations was not mandatory (eMethods in Supplement 2). For patients in the control arm, genotyping results were available only at the end of the study, and treatment was started according to the standard-of-care dosing.

**Table 1.** Frequency of Genotype-Based Phenotypes and Recommended Dose Adjustments

Patients treated with fluoropyrimidines and/or irinotecan^a^	Control arm (n = 311), No. (%)	Intervention arm (n = 252)	
		No. (%)	Pharmacogenetic-informed treatment according to DPWG guidelines
Actionable genotype carriers	23 (7.4)	17 (6.7)	
*DPYD* AS 0.5	1 (0.3)	0	Avoid fluoropyrimidines or perform DPD phenotyping
*DPYD* AS 1	6 (1.9)	2 (0.8)	50% of the standard dosage
*DPYD* AS 1.5	7 (2.3)	11 (4.4)	50% of the standard dosage
*UGT1A1* PM	9 (2.9)	4 (1.6)	70% of the standard dosage
Nonactionable genotype carriers (*DPYD* AS 2, *UGT1A1* NM/IM)	288 (92.6)	235 (93.3)	Standard dosage

Abbreviations: AS, activity score; DPD, dihydropyrimidine dehydrogenase; DPWG, Dutch Pharmacogenetics Working Group; IM, intermediate metabolizer; NM, normal metabolizer; PM, poor metabolizer.

^a^ Activity scores for *DPYD* and metabolizer status for *UGT1A1* were assigned based on PharmGKB Clinical Guideline Annotations.^30^

### Clinical and Demographic Data Collection

Clinical and demographic characteristics, including toxic effect data, were planned to be recorded at baseline, 4 and 12 weeks postinclusion, and at the end of treatment (eMethods in Supplement 2). Utility values to assess quality of life^27^ and calculate the quality-adjusted life-years (QALYs) were collected at the same time points. Additional clinical data (ie, tumor location, treatment regimen, schedule, and setting) were extracted from the patients’ clinical records in accordance with the study objectives. For patients with actionable genotypes, the number of chemotherapy cycles, days of suspension, milligrams of dose intake for each drug administration, planned duration of treatment, and reasons for treatment discontinuation were collected for dose intensity calculation.

### Outcomes

Adverse drug reaction severity and causality were evaluated by trained assessors (L.F. and M.G.). Severity was classified using the Common Terminology Criteria for Adverse Events (CTCAE) classification, version 4.0, and causality with the Liverpool Causality Assessment Tool. Only ADRs categorized as definitely, probably, or possibly related to fluoropyrimidines and/or irinotecan were considered to be likely related. According to the PREPARE trial, a severe toxic effect was categorized as clinically relevant in case of likely related CTCAE grade 4 or higher hematologic or grade 3 or higher nonhematologic toxic effects, or when hospitalization was needed for toxic effect management.^27^

The primary outcome was the development of at least 1 clinically relevant toxic effect. Additional health economics and efficacy outcomes included hospitalization rate for any toxic effect management (regardless of the causality assessment) and related costs, quality of life, and intensity of treatment. Toxic effect management costs were collected as described within the PREPARE trial.^31^

Dose density, expressed as a percentage, was evaluated for fluoropyrimidine and/or irinotecan treatment and referred to the entire treatment course. The overall amount of drug received, by unit of time (considering dose reductions, treatment delays, and treatment interruptions), was compared with the overall amount expected based on the standard chemotherapy schedule and dosing regimen. This evaluation included only patients with actionable genotypes and complete chemotherapy information (eMethods in Supplement 2). Overall survival (OS) was assessed at 3 years since inclusion in the PREPARE trial.

### Statistical Analysis

Data analysis was performed from May 27 to October 10, 2024. Logistic regression analysis was used to estimate crude and adjusted odds ratios (ORs) with the corresponding 95% CIs. Due to the cost distribution, quantile regression was used to model the median and 75th percentile of total toxic effect costs, adjusting for sex and age. For the main toxic effect outcome, according to the PREPARE study protocol, a gatekeeping analysis was adopted. Briefly, the risk of developing clinically relevant toxic effects was compared between patients with an actionable genotype in the intervention arm vs those in the control arm, and an analysis of all patients was performed only if this difference was statistically significant^27^ (eMethods in Supplement 2).

## Results

### Demographic and Clinical Characteristics

Among the 1232 patients enrolled at the Italian implementation sites, 563 patients were included in this secondary analysis: 311 in the control arm and 252 in the intervention arm (Figure 1). A total of 246 women (43.7%) and 317 men (56.3%) were included; median age was 68.0 (IQR, 60.0-75.0) years. Further baseline characteristics are described in eTable 1 in Supplement 3. Median follow-up was 348 (IQR, 201-540) days. Almost all patients (553 [98.2%]) were of self-reported European ethnicity; the remaining 1.8% were from other ethnicities (2 patients were of self-reported African ethnicity, 4 Hispanic, 3 West Asian, 1 did not reply to the question). A total of 412 hematologic and 1723 nonhematologic toxic effects were recorded (eResults and eFigure 1 in Supplement 2).

### Description of Pharmacogenetic Characteristics

Among the 50 polymorphisms tested, data for *DPYD* and *UGT1A1* were extracted and used for this secondary analysis (Table 1; eTable 2 in Supplement 3). The prevalence of carriers of an actionable genotype was 23 of 311 (7.4%) in the control arm and 17 of 252 (6.7%) in the intervention arm (eTable 3 and eTable 4 in Supplement 3). All treating physicians reduced the dose according to the Dutch Pharmacogenetics Working Group pharmacogenetic guidelines in all 17 patients with an actionable genotype in the intervention arm (Table 1). No patient in the intervention arm was a carrier of more than 1 *DPYD* polymorphic variant or more than 2 *UGT1A1* polymorphic variants.

### Comparison of Clinically Relevant Toxic Effects Between Arms

Considering the toxic effects that developed throughout the course of therapy with the index drug, 91 patients (16.2%) developed clinically relevant toxic effects causally related to fluoropyrimidines or irinotecan treatment. Among these patients, 83 (91.2%) experienced at least 1 grade 4 or higher hematologic toxic effect or grade 3 or higher nonhematologic toxic effect during treatment with the index drug, whereas 8 patients (8.8%) were included because they warranted hospitalization for toxic effect management. In the intervention arm, 1 of 17 patients (5.9%) with an actionable genotype developed clinically relevant toxic effects, compared with 8 of 23 (34.8%) in the control arm (OR, 0.1; 95% CI, 0.0-0.8; *P* = .04, adjusted for age and sex). Since the difference was statistically significant and the first gatekeeping analysis^27^ was passed, we subsequently extended the analysis to all patients. The difference was not significant (Table 2).

**Table 2.** Difference in the Incidence of Clinically Relevant Toxic Effects Between Arms^a^

Variable	Patients, No.	Patients with clinically relevant toxic effects, No. (%)	OR (95% CI)^b^	*P* value^b^
Any actionable genotype carriers				
Control arm	23	8 (34.8)	1 [Reference]	.04
Intervention arm	17	1 (5.9)	0.1 (0.0-0.8)	
All patients				
Control arm	311	54 (17.4)	1 [Reference]	.41
Intervention arm	252	37 (14.7)	0.8 (0.5-1.3)	
Subanalysis focusing only on the actionable *DPYD* genotype carriers				
Control arm	14	5 (35.7)	1 [Reference]	.04
Intervention arm	13	0	NA	

Abbreviations: NA, not applicable; OR, odds ratio.

^a^ Gatekeeping analysis was conducted for patients with an actionable genotype and all patients, irrespective of genotype.

^b^ Multivariate logistic regression adjusted for age and sex.

### Comparison of Hospitalizations and Costs Between Arms

During the study follow-up, 76 of 563 patients (13.5%) were hospitalized for any toxic effect management. In the intervention arm, 2 of 17 patients (11.8%) with an actionable genotype were hospitalized, compared with 8 of 23 (34.8%) in the control arm (*P* = .12). Among all patients, 35 of 252 (13.9%) in the intervention arm were hospitalized vs 41 of 311 (13.2%) in the control arm (*P* = .79) (Table 3).

**Table 3.** Difference in the Incidence of Patients Hospitalized for Toxic Effect Management and the Toxic Effect Management Costs

Variable	Patients, No.	Hospitalized patients, No. (%)	OR (95% CI)^a^	*P* value^a^	Adjusted cost (95% CI), $^b^	*P* value^a^
Any actionable genotype carriers						
Control arm	23	8 (34.8)	1 [Reference]	.12	4159 (1510-6810)	.004
Intervention arm	17	2 (11.8)	0.25 (0.04-1.42)		26 (0-312)	
All patients						
Control arm	311	41 (13.2)	1 [Reference]	.79	50 (25-76)	.11
Intervention arm	252	35 (13.9)	1.07 (0.66-1.73)		25 (3-48)	
Subanalysis focusing only on the actionable *DPYD* genotype carriers						
Control arm	14	5 (35.7)	1 [Reference]	.26	4168 (1481-6854)	.006
Intervention arm	13	2 (15.4)	0.34 (0.05-2.24)		23 (0-522)	

Abbreviation: OR, odds ratio.

^a^ Multivariate logistic regression adjusted for age and sex.

^b^ Cost description is based on quantile regression; costs reported at the 75th percentile.

The highest toxic effect management cost per patient (75th percentile) was observed in the control arm for actionable genotype carriers ($4159; 95% CI, $1510-$6810) compared with the intervention arm ($26; 95% CI, $0-$312) (*P* = .004). Median hospitalization costs were $47 in the control arm and $10 in the intervention arm. For all patients, toxic effect management costs (75th percentile) were not significantly different between arms, with median costs of $8 in the control arm and $0 in the intervention arms (Table 3).

### Comparison of Outcomes Between Arms for Patients With Actionable Genotype for *DPYD* Only

As a subanalysis, toxic effect and health economics outcomes were compared between arms, focusing on actionable *DPYD* genotype carriers for fluoropyrimidine prescription. Regarding the primary outcome (clinically relevant toxic effects), none of the 13 actionable *DPYD* carriers in the intervention arm developed toxic effects, compared with 5 of 14 patients (35.7%) in the control arm (OR not assessable; *P* = .04) (Table 2). Hospitalization rates were lower in the intervention arm, with 2 of 13 patients (15.4%) hospitalized, compared with 5 of 14 (35.7%) in the control arm (Table 3). The highest toxic effect management cost at the 75th percentile was $4168 (95% CI, $1481-$6854) for actionable *DPYD* carriers in the control arm compared with $23 (95% CI, $0-$522) (*P* = .006) for noncarriers. Median hospitalization costs for actionable *DPYD* carriers were $56 in the control arm and $0 in the intervention arm.

### Intensity of Treatment and Efficacy Outcomes

**Figure 2.**
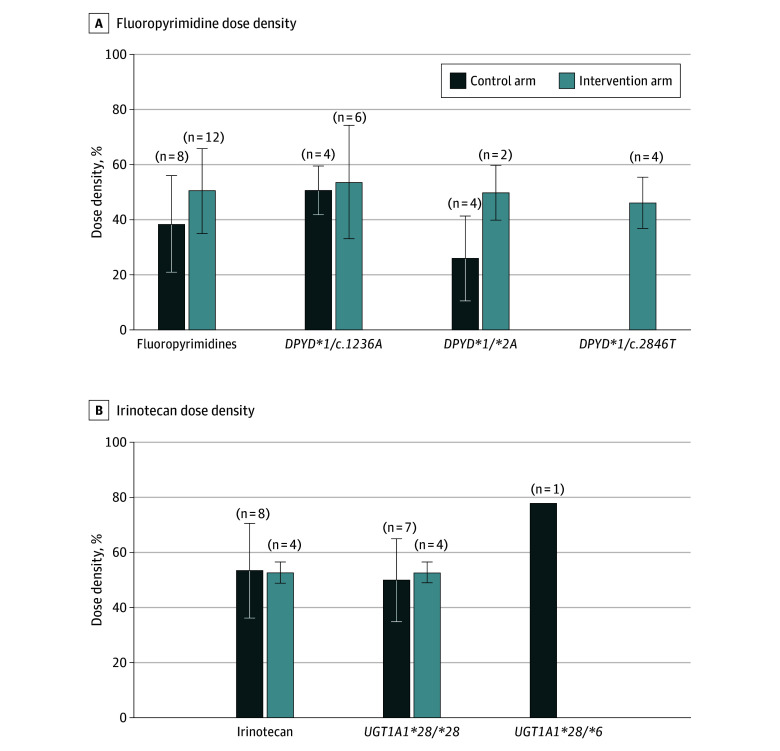
Descriptive Analysis of Fluoropyrimidines and/or Irinotecan Intensity of Treatment This analysis was performed in a subgroup of patients (n = 32) with actionable genotypes and complete chemotherapy information. Distribution of mean percentage values of dose density for patients treated with fluoropyrimidines in carriers of any *DPYD* variant (fluoropyrimidines) or specific *DPYD* genotypes (*DPYD* *1/c.1236A; DPYD*1/*2A; and DPYD*1/c.2846T) and irinotecan in carriers of any *UGT1A1* variant (irinotecan) or specific *UGT1A1* genotypes (UGT1A1*28/*28; and UGT1A1*28/*6) in both control and intervention arms. One patient in the group DPYD*1/*2A is a compound heterozygous carrier with genotype DPYD*2A/c.1236A. Error bars indicate SDs.

An analysis of fluoropyrimidine and irinotecan dose density throughout the entire course of therapy was performed on 16 of 23 (69.6%) patients in the control arm and 16 of 17 (94.1%) in the intervention arm, with an actionable genotype and complete chemotherapy data (Figure 2; eFigure 2 in Supplement 2). The mean (SD) fluoropyrimidine dose density in patients with any *DPYD* actionable genotype was 38% (18%) in the control arm, and 51% (15%) in the intervention arm. The mean (SD) irinotecan dose density was 54% (17%) for patients with any *UGT1A1* actionable genotype in the control arm, and 53% (4%) in the intervention arm. Four patients with a DPYD*1/*2A genotype in the control arm had a mean fluoropyrimidine dose density of 26% (15%) compared with 2 patients in the intervention arm (mean, 50% [9%]) (Figure 2; eTable 5 in Supplement 3 and eResults in Supplement 2).

A further exploratory analysis was focused on 17 patients receiving a combination regimen including both fluoropyrimidines and irinotecan. Mean (SD) fluoropyrimidine dose density for *UGT1A1* variant carriers was 59% (16%) in the control arm and 70% (8.5%) in the intervention arm. Mean irinotecan dose density for *DPYD* variant carriers was 71% (18%) in the control arm and 67% (12%) in the intervention arm (eFigure 3 and eResults in Supplement 2).

Three-year overall survival (OS) data were not significantly different between the control and intervention arms limited to patients with an actionable genotype or for all patients. At 48 months, there were 109 deaths recorded in the control arm and 104 in the intervention arm. The number of deaths at 48 months was 8 in the control arm and 6 in the intervention arm in patients with an actionable genotype.

### Quality-Adjusted Life-Years

Comparative analysis of QALYs in a subset of 491 patients with complete data (n = 281 in control arm and n = 210 in intervention arm) found a mean QALY of 62% (29%) in the intervention arm vs 53% (26%) (*P* = .001, Mann-Whitney test) in the control arm. Pairwise comparison based on genotype actionability and arm of treatment is reported in eFigure 4 in Supplement 2.

## Discussion

This single-country secondary analysis of the PREPARE trial^27^ suggests the potential clinical benefit and effectiveness of a pharmacogenetic panel, consisting of *DPYD* and *UGT1A1*, in patients with GI cancer treated with fluoropyrimidines and/or irinotecan, drugs that are frequently prescribed in this oncology setting. Carriers of an actionable genotype whose treatment was adjusted based on panel results exhibited a 90% reduction in the incidence of clinically relevant toxic effects compared with carriers in the control arm. This finding reflects the 30% reduction in the rate of ADRs in the intervention arm compared with the control arm in the main PREPARE trial.^27^ In this analysis of a selected and clinically homogeneous subpopulation, this difference was nonsignificant when considering the whole patient population, likely due to the low prevalence of patients with an actionable genotype as described previously for other rare variants.^32^

This analysis also showed that prospectively applying a *DPYD/UGT1A1* genotyping approach normalizes the risk of ADR-related hospitalization compared with patients without a variant and receiving the standard of care. This aligns with work by Henricks et al^33^; however, our study uniquely compared genotype-adapted dosing within a prospective clinical trial. In the control arm, patients with an actionable genotype were at higher risk of being hospitalized for toxic effects and incurred higher toxic effects management costs. Our findings reaffirm the significance of hospitalization as the primary factor in cost. Previous work reported that a combined *DPYD/UGT1A1* genotyping approach not only increased the number of patients with high cost management identified by genotyping compared with a single-gene approach, but also increased the incremental cost between patients with actionable and nonactionable genotypes.^20,21^ In this study, when restricting the analysis to fluoropyrimidine/*DPYD* actionable genotype carriers, the pattern of association remained consistent with the overall cohort but capturing a lower number of patients at risk of toxic effects, highlighting the advantage of a combined genotyping approach. The increased cost-effectiveness of multigene panel testing was already proven to be critically dependent on incidental findings in other clinical contexts.^34^ To the extent that multigene panel testing becomes a reliable alternative to single-gene testing, and considering the frequency of simultaneous or sequential irinotecan and fluoropyrimidine administration in patients with GI cancer,^23^ the assessment of all actionable genotypes at the start of treatment may be associated with greater cost-effectiveness.^34^

This study also tested for what was, to our knowledge, the first time, the prospective application of a *DPYD*-based fluoropyrimidine dose reduction using the most recent pharmacogenetic guidelines.^17,35^ Specifically, an upfront 50% fluoropyrimidines dose reduction was applied for heterozygous carriers of *DPYD* variants *c.1236G>A*, *c.2846A>T*, DPYD*13, or DPYD*2A, regardless of the activity score (range, 1-1.5), instead of the previously recommended 25% dose reduction recommended for heterozygous carriers of *DPYD-c.1236G>A* and *DPYD-c.2846A>T* (activity score 1.5).^36^

Despite the increasing adoption of *DPYD* genotyping before fluoropyrimidine treatment in many countries, particularly in Europe,^37,38^ it has yet to become standard in others, such as the US,^25,39^ due to concerns about decreasing treatment intensity and anticancer efficacy.^26^ Although this study was not designed to address the impact of dose reduction on treatment efficacy, our data suggest that pharmacogenetic-guided dose reductions in fluoropyrimidines and/or irinotecan may not affect the intensity of the treatment. Patients starting therapy at the full dose often require dosage reductions, treatment delays, and premature discontinuations due to ADRs, leading to underexposure. For impactful mutations, such as DPYD*2A, treatment intensity appears lower in patients starting with a full fluoropyrimidine dose compared with those starting with a safe halved dose in the intervention arm. This also seemed to affect the intensity of concomitantly administered drugs not directly impacted by the mutation, such as irinotecan, as the common clinical practice is to reduce the dose or delay the administration of all chemotherapeutic agents after the occurrence of clinically relevant toxic effects. These data, although exploratory, are, to our knowledge, the first evidence of the association between a pharmacogenetic-informed approach and treatment adherence and intensity. The front-line dose reduction in patients at risk for toxic effects appeared not to negatively affect the OS compared with standard of care as in other studies.^40^

Patients in the intervention arm experienced a quality-of-life advantage despite not carrying an actionable genotype, likely due to the positive psychological impact of pretreatment DNA testing. Improvements in patient-reported outcomes, including quality of life, for patients treated with personalized approaches have been reported in oncology.^41^ In addition, other prescriptions received during 18-month follow-up may have been adapted according to the pharmacogenetic profile, further improving quality of life, although this aspect was not formally analyzed.

### Limitations

Some limitations of the study should be considered. The PREPARE trial was designed as an open-label trial, which could have affected the ADR reporting. This secondary analysis includes data from a single country, where patients were enrolled according to a sequential study design, potentially influencing results. However, we focused only on documented severe clinical toxic effects, with independent systematic review of severity and causality by 2 trained investigators. The PREPARE trial was not designed to assess improvements in survival, and further studies are needed for comprehensive evaluation. Our analysis suggested that treatment intensity was not affected or even improved in patients treated according to their genotype, with comparable OS between the intervention and control arms. Additionally, the variants analyzed primarily focused on the European population, necessitating further exploration of variants more prevalent in other populations.^42,43^

## Conclusions

The findings of this single-country secondary analysis stemming from the PREPARE cluster-randomized clinical trial appear to support integrating a panel-based pharmacogenetic testing strategy in patients with GI cancer undergoing fluoropyrimidine or irinotecan treatment. The pharmacogenetic-informed approach of *DPYD* and *UGT1A1* testing reduced the percentage of patients developing clinically relevant toxic effects and normalized the hospitalization risk and costs, while increasing patients’ quality of life. An exploratory analysis on efficacy outcomes highlighted that the approach did not apparently affect treatment intensity or OS.

## Notes

Supplement 1.
Trial Protocol

Supplement 2. **eMethods.** Detailed Methods **eResults.** Detailed Results **eFigure 1.** Frequency of Toxicity According to Grade and Causality and Hospitalization Costs **eFigure 2.** Swimmer Plot: Case-by-Case Description of Treatment Adherence for Actionable Genotypes Carriers **eFigure 3.** Crossed Drug/Gene Comparison of Dose Density **eFigure 4.** Quality-Adjusted Life Years
eReferences

Supplement 3. **eTable 1.** Baseline Demographic and Clinical Characteristics **eTable 2.** Frequency of *DPYD* and *UGT1A1* Genotypes **eTable 3.** Demographic and Clinic Characteristics of the Patients With an Actionable Genotype in the Control Arm **eTable 4.** Demographic and Clinic Characteristics of the Patients With an Actionable Genotype in the Study Arm **eTable 5.** Mean Dose Density Percentage Values Between Control and Intervention Arms for Fluoropyrimidines and Irinotecan

Supplement 4.
Data Sharing Statement
