# Supplementary material for: Clinical Benefits and Utility of Pretherapeutic DPYD and UGT1A1 Testing in Gastrointestinal Cancer: A Secondary Analysis of the PREPARE Randomized Clinical Trial
Source: JAMA Netw Open. 2024 Dec 6;7(12):e2449441. doi: 10.1001/jamanetworkopen.2024.49441 (PMC11624585; doi:10.1001/jamanetworkopen.2024.49441)

CONFIDENTIAL

CLINICAL STUDY PROTOCOL:

PREemptive Pharmacogenomic testing for Preventing Adverse drug REactions

(PREPARE)

March 4th 2022, Protocol Framework

Version 8.0

On behalf of The Ubiquitous Pharmacogenomics Consortium

U-PGx | Ubiquitous Pharmacogenomics

Change History:

Version	Date	Change	Original	Revised	Page in relevant version
**1.0**	**October 2016 (never submitted in NL)**	-	-	-	
**2.0**	**November 2016 (initial submission in NL)**	Removal of buccal swab to be collected	Patients who endure an ADR which is categorized as an “extreme phenotype” will be asked to provide an additional blood spot sample and (buccal) saliva sample at the time of the ADR.	Patients who endure an ADR which is categorized as an “extreme phenotype” will be asked to provide an additional blood spot sample within 24 hours of the ADR.	43
		Addition of information on logistics if a patient discontinues with the index drug	-	If a patient discontinues the index drug during the 12 week follow-up period, follow-up will also discontinue. However, the cross-sectional end-of study survey will be performed.	15
		Addition of information on LIM registration	-	Research nurses will register patients for the LIM tool at baseline. Their study-ID number, e-mail address, drug of inclusion and initiation date will be reported in the LIM registration portal. An automated e-mail will be sent to the patient at week two and eight after drug initiation. This e-mail will include a link to the LIM portal. Patients will be asked to log-in to the LIM portal using their e-mail address and a standard password. Patients will be asked to change this password once they log in. This will give patients access to the LIM surveys.	15
		Addition of information on local storage of patient contact information	-	Research nurses will collect and store patient contact information (e-mail address, telephone number, name and study-ID number) in a separate, local file (national subject identification code list).	16
		Removal of sample and PGx result logistics	Inpatients must provide a blood sample at least 24 hours after signing informed consent, but preferably directly after providing informed consent. Outpatients will provide a saliva sample at least 24 hours after inclusion, but preferably directly after providing informed consent. Samples will be sent to the local genetic laboratory for analysis. Once the genetic laboratory has received the blood or saliva sample PGx results must be provided to the prescribing physician or dispensing pharmacist within 3 working days.	-	19
		Additional information on DNA sample collection and storage	-	Blood samples will be stored at 2-8°C before DNA isolation. Saliva samples will be collected using the ORAGENE DNA 500 collection kit and stored at room temperature.	26

		Addition and removal of information regarding patient recruitment logistics	Patients who have previously started with this prescription for a maximum of 48 hours are still be eligible for inclusion. In this case the time of first dose will be noted in the eCRF.	Patients who are planned to receive a first prescription for a drug listed in Table 5 are also eligible for inclusion and may be recruited in the study. The time of the first dose of the index drug (t=0) will be noted in the eCRF.	31
		Addition of information on blood plasma sub-study	-	See Chapter 6.3.3. on page 49.	49
**3.0**	**February 2017**	Exemption of patient insurance		Removal of patient insurance information	59
**4.0**	**March 2017**	Revised wording of secondary outcomes	2) reduces the total number of 'serious' ADRs (causally related to both the index drug and subsequent drug)	2) reduces the total number of 'serious' ADRs (causally related to the index drug)	17
		Revised information on logistics if a patient discontinues the index drug	If a patient discontinues the index drug during the 12 week follow-up period, follow-up will also discontinue. However, the cross-sectional end-of study survey will be performed.	When the index drug is discontinued within the twelve week follow-up period, the follow-up will continue as initially anticipated.	20
		Revised LIM logistics	If patients are not able to do so, research nurses will assist the patients.	If patients do not have an email address or are not able to fill in an online survey, then the LIM tool will not be used.	20
		Addition of information in LIM portal	-	The patient's date of birth is recorded in the LIM registration portal	20
		Summary matrix for data collection is updated to fit eCRF database structure	-	See Chapter 3.1.2. on page 22.	22
		Clozapine was removed from the list of drugs of inclusion due to removal of CYP1A2 from panel			29
		Addition of information regarding PGx result logistics	-	PGx results related to the drug of interest must be returned to the treating HCP within seven days of index drug initiation (the three working day turnaround time for genotyping after receiving the sample is included within these seven days)	32
		Removal of patient exclusion in study arm if logistical requirements are not met	PGx-results regarding the gene of interest must be reported to the physician/pharmacist within one week of index drug initiation. The "Safety-Code card" may be provided after this week. If this is not complied, the patient is excluded.	Study arm: PGx-results regarding the gene of interest must be reported to the physician/pharmacist within one week of index drug initiation. The "Safety-Code card" may be provided after this week. If this is not complied then the patient will be excluded from the data set in a sensitivity analysis (see statistical analysis plan for details).	32
		Removal of patient exclusion in control arm if logistical requirements are not met	Patients must provide informed consent and provide a DNA sample within one week of index drug initiation. The mock "Safety-Code card" may be provided after this week. If this is not complied, the patient is excluded.	Control arm: patients must provide informed consent and provide a DNA sample within one week of index drug initiation. The mock "Safety-Code card" may be provided after this week. If this is not complied then the patient will be excluded from the data set in a sensitivity analysis (see statistical analysis plan for more detail).	32

		Addition of index drug in patient log- book	The recruiting physicians and pharmacists shall note all patients (including their age, sex and indication) that are considered for participation in their study logbook.	The recruiting physicians and pharmacists shall note all patients (including their age, sex, index drug and indication) that are considered for participation in their study logbook.	32
		Handling missing data in more detail	Data from patients who withdraw after 12 weeks follow-up will be included in the primary analyses. When patients withdraw before 12 weeks follow-up, they will be included in secondary analyses.	How missing data is handled in the analyses is described in detail in the statistical analysis plan.	34
		Panel was edited due to expert discussion	See 5.1.1. on page 36	See 5.1.1. on page 37	37
		Revised wording of primary endpoint	The primary endpoint is the frequency of causal (definite, probable or possible), clinically relevant (classified as NCI-CTCAE grade 2, 3, 4, or 5), drug-genotype specific ADRs, for the index drug, for each patient.	The primary outcome is the occurrence of at least one causal (definite, probable or possible), clinically relevant (classified as NCI-CTCAE grade 2, 3, 4, or 5), drug-genotype specific ADR, attributable to the index drug, within 12 weeks of follow-up.	43
		Different version of NCI-CTCAE will be used to assess severity	NCI-CTCAE version 5.0 will be used to assess severity	NCI-CTCAE version 4.0 will be used to assess severity	45
		Addition of information on severity and causality re-assessments	-	All causality and severity assessments will be reassessed by Lareb to independently validate these assessments. These assessors will be blinded to patient's arm allocation. The methods used by the assessors will be described in a Standard Operating Procedures 4.8 and 4.9.	45,46
		Addition of information on drug-genotype association assessment	-	The drug-genotype association assessment will be performed by an expert panel, once the study is completed. The assessor is blinded to patient's arm allocation. The methods used by the assessors are described in a Standard Operating Procedure 4.10.	48
		Addition of information on drug-drug-gene sub-study	-	See Chapter 6.3.3. on page 40.	50
		Revised sample collection when a patient endures a serious ADR	Patients who endure an ADR which is categorized as an "extreme phenotype" will be asked to provide an additional blood spot sample within 24 hours of the ADR.	Patients who endure an ADR which is categorized as an "extreme phenotype" will be asked to provide an additional blood sample (10mL EDTA) within 24 hours of the ADR.	54
**5.0**	**June 2017**	Incidence of drug adherence questions increased	Asked at baseline and end of study	Asked at baseline, 4 weeks, 12 weeks and end of study	22
		Incidence of PGx knowledge questions increased	Asked at baseline and end of study	Asked at baseline, 12 weeks and end of study	22
		Windows for 4 week and 12 week survey expanded	± 1 week	± 2 weeks	21
		ADE report and data collection		Patients must be contacted by the research team withtin the described time windows for data collection. However,	47

		described in more detail		patients and physicians may also report ADEs outside these time windows. ADEs may also be derived from (electronic) medical records	
**6.0**	**March 2018**	Addition of specific criteria for withdrawal	-	Patient does not use index drug for a minimum of 7 consecutive days	32
		Adjustment of primary composite endpoint definition regarding oncological patients	-	For oncology patients receiving 5-FU, capecitabine, tegafur or irinotecan, only hematological toxicities of NCI-CTCAE grade 4-5 and non-hematological toxicities of NCI-CTCAE grade 3-5 will be considered clinically relevant.	40
		Adjustment of extreme phenotype definition	1.Experience a serious ADR which is not expected on the basis of the pre-emptive PGx testing results.<br>2.Experience a serious ADR (already known to be associated with the drug in the DPWG guidelines, Table 7) even though the patient had received an altered drug or dose selection as a result of an actionable genotype.	See Figure 10 on page 49	49
		Removal of carbamazepine from index drug list	-	Removal of carbamazepine from index drug list.	28
		Removal of HLA-B*1502 and HLA-A*3101 from panel	50 variants in 13 pharmacogenes	46 SNPs (to determine 44 variants) in 12 pharmacogenes	34
		Decrease the number of severity and causality assessments for validation	All causality and severity assessments will be reassessed by Lareb to independently validate these assessments.	10% of causality and severity assessments will be reassessed by Lareb to independently validate these assessments.	41, 43
		Completion of appendix by addition of warfarin DPWG recommendations	-	Addition of recommendations related to warfarin.	85
**6.0**	**February 2019**	Windows for 12 week survey expanded	± 2 weeks	± 3 weeks	21
		Enrolment logistics surrounding cross-over	It is anticipated that the study will start in January 2017 and end in December 2019.	It was anticipated that the study would start in January 1st 2017 and end in December 31st 2019. Due to a delay, the study started in March 6th 2017 and the cross-over was performed on October 1st 2018.	30
		Update of recruiting centers and their corresponding areas of focus	-	See Table 4	31
		Removal of oxycodone from index drug list	-	DPWG guidelines are periodically updated and these changes need to be reflected in the protocol. As of February 20th 2018, the DPWG guideline for oxycodone was updated. The updated version no longer had an actionable recommendation for any phenotype. As a result, oxycodone	33, 34, 42

				was removed as an index drug as per May 15th 2018.	
		Timing of drug-genotype association assessment	The drug-genotype association assessment will be performed by an expert panel once the study is completed.	The drug-genotype association assessment will be performed by an expert panel in bulk.	51
		Addition of CYP2B6*5 and NUDT15 *3, *6 and *9 to panel		CYP2B6*5 (rs3211371), NUDT15*3 (rs116855232), NUDT15*6 (rs869320766) and NUDT15*9 (rs746071566).	39
**7.0**	**March 2020**	Extension of time-block 2 enrolment with 3 months	The first time block will encompass an 19 month enrolling period (and an additional 3 months follow-up) and the second time block will encompass an 18 month enrolling period (and an additional 3 months follow-up), where a new set of patients is recruited in each arm.	The first time block will encompass an 19 month enrolling period (and an additional 3 months follow-up) and the second time block will encompass an 21 month enrolling period (and an additional 3 months follow-up), where a new set of patients is recruited in each arm.	20
			It was anticipated that the study would start in January 1st 2017 and end in December 31st 2019. Due to a delay, the study started on March 6th 2017 and the cross-over was performed on October 1st 2018.	It was anticipated that the study would start in January 1st 2017 and end in December 31st 2019. Due to a delay, the study started on March 6th 2017 and the cross-over was performed on October 1st 2018. The second time-block is extended with a 3 month enrolment period from April 1st 2020 to July 1st 2020.	29
**8.0**	**March 2022**	Details on the primary endpoint and statistical analysis are removed from the protocol and specified in a separate Statistical Analysis Plan (SAP) version 3.0		6.1 figure 8 was updated<br>6.1.3. Drug-genotype association was removed<br>7 for the statistical analyses a refence is made to the SAP 3.0	53

CLINICAL STUDY PROTOCOL:

PREemptive Pharmacogenomic testing for Preventing Adverse drug REactions

(PREPARE)

Protocol ID	Grant Agreement N° 668353 (U-PGx)
Protocol Name	**PREPARE**
Version	**7.0**
Date	**March 27th 2020**
Coordinating investigator	**Jesse J. Swen, PharmD, PhD**<br>**Associate Professor of Pharmacogenetics**<br>**Dept. Clinical Pharmacy & Toxicology**<br>**Leiden University Medical Center**<br>**P.O. Box 9600**<br>**NL 2300 RC Leiden**<br>**The Netherlands**<br>**Tel.: +31 (0)71 526 2790**<br>**Fax: +31 (0)71 526 6980**<br>**E-mail: J.J.Swen@lumc.nl**
National Principal investigator(s):<br>**Leiden University Medical Center: (LUMC)**<br>**University of Liverpool: (PHUL)**<br>**Medical University of Vienna: (MUWV)**<br>**Centro di Riferimento Oncologico: (CROA)**<br>**Servicio Andaluz de Salud: (SASG)**<br>**University of Patras: (UPAT)**<br>**University of Ljubljana: (ULMF)**	(see Appendix 18.1 for contact information)<br>**Jesse J. Swen**<br>**Munir Pirmohamed**<br>**Gere Sunder-Plassmann**<br>**Giuseppe Toffoli**<br>**Cristina Lucía Davila Fajardo**<br>**George P. Patrinos**<br>**Vita Dolzan**
Sponsor	**Leiden University Medical Center**<br>**Albinusdreef 2, 2333 ZA, Leiden**
Subsidising party	**European Commission, Horizon 2020**<br>**Grant Agreement N°668353 (U-PGx)**
Independent expert	**Dr. Gijs Santen**<br>**Klinisch Geneticus, LUMC**<br>**Albinusdreef 2**<br>**2333 ZA Leiden**<br>**Tel: 071 5268033**<br>**E-mail: g.w.e.santen@lumc.nl**
Laboratory sites	**FARMACOGENOMIC TESTING:**<br>**LUMC: Department of Clinical Pharmacy and Toxicology, Albinusdreef 2, 2223 ZA, Leiden Building 1**<br>**Tel: (071) 526 2790 Fax: (071) 526 6866**<br>**E-mail: KFTsecretariaat@lumc.nl**<br><br>**NEXT GENERATION SEQUENCING OF 'EXTREME PHENOTYPES':**

	**Section of pharmacogenetics/ Department of Physiology and Pharmacology, Karolinska Institutet, Nanna Svartz vag 2, SE 171 77 Stockholm, Sweden**<br>**Tel: +46852487735**<br>**Contact: Prof. Dr. Magnus Ingelman-Sundberg**<br>**E-mail: Magnus.Ingelman-Sundberg@ki.se**<br><br>**<u>DRUG-DRUG-GENE INTERACTION SUB-STUDY:</u>**<br>**Dr. Margarete Fischer-Bosch-Institute of Clinical Pharmacology**<br>**Auerbachstr. 112**<br>**D-70376 Stuttgart, Germany**<br>**Tel: ++49-711-8101 3700**<br>**Fax: ++49-711-859295**<br>**Contact: Prof. Dr. Matthias Schwab**<br>**E-mail: Matthias.Schwab@ikp-stuttgart.de**

The clinical study will be conducted, and essential documentation archived, in compliance with EU Directives, which incorporate the requirements of the ICH Guideline for Good Clinical Practice.

PROTOCOL SIGNATURE PAGE

Name	Signature	Date
**Head of Department:**<br>**Prof. Dr. Henk-Jan Guchelaar**<br>**Professor of Clinical Pharmacy**<br>**Dept. Clinical Pharmacy & Toxicology**<br>**Leiden University Medical Center**		**04-03-2022**
**Coordinating Investigator/Project Leader:**<br>**Jesse J. Swen, PharmD, PhD**<br>**Section Head Laboratory**<br>**Associate Professor of Pharmacogenetics**<br>**Dept. Clinical Pharmacy & Toxicology**<br>**Leiden University Medical Center**		**04-03-2022**
**Principal Investigator NL:**<br>**Jesse J. Swen, PharmD, PhD**<br>**Section Head Laboratory**<br>**Associate Professor of Pharmacogenetics**<br>**Section Chair Laboratory**<br>**Dept. Clinical Pharmacy & Toxicology**<br>**Leiden University Medical Center**		**04-03-2022**

PROTOCOL SYNOPSIS

Rationale: In recent years, multiple randomized controlled trials for a variety of drug-gene combinations have strongly indicated that pharmacogenomics (PGx) testing prior to prescribing, to guide the dose and drug selection, can improve patient outcomes. Almost 15% of medicinal products evaluated by the European Medicines Agency between 1995 and 2014 contain PGx information in their product label. PGx-guided therapeutic dose and drug selection recommendations have also been created and published by the Dutch Pharmacogenetics Working Group (DPWG). However, despite these major scientific and clinical advances in PGx, and the availability of several commercially available PGx tests, the application of PGx into routine care remains very limited. PREPARE will implement pre-emptive genotyping of an entire panel of clinically relevant PGx markers (for which DPWG guidelines are available: 'pharmacogenes') across seven countries within the European Union, in a prospective clinical study and investigate its collective impact on patient outcomes and cost-effectiveness.

Objective: To determine whether implementing pre-emptive PGx testing of an entire panel of clinically relevant PGx markers, to guide the dose and drug selection for 39 commonly prescribed drugs, will result in an overall reduction in the number of clinically relevant drug-genotype associated adverse drug reactions (ADRs). We hypothesize that the implementation of PGx-guided drug prescribing will reduce both the occurrence and severity of drug-genotype associated ADRs in comparison to patients receiving standard of care treatment.

Study design: A multi-center, open, randomized, cross-over implementation study conducted in seven countries across Europe. Countries will be randomised to start with enrolling either patients receiving PGx-guided prescribing (study arm) or standard of care (control arm) for 19 months. After this period, a new set of patients will be recruited and the opposite strategy will implemented for 21 months. All study patients will be followed-up for a minimum of 12 weeks; maximum follow-up is limited to 22 months per patient.

Study population: Patients of any ethnicity who are at least 18 years old and receive a first prescription for a drug for which a DPWG guideline is available, as part of routine care, are eligible to participate. 4,050 patients will receive PGx-guided drug and dose selection (study group) and 4,050 patients will receive standard of care (control group).

Intervention: All patients will donate a DNA sample that will be genotyped for a panel of 48 genetic variants in 13 pharmacogenes. For patients within the study arm, their results will be: 1) recorded in the (electronic) medical record and 2) provided to the patient in the form of plastic card, akin to a credit card. Genetic results and DPWG guidelines can used by physicians and pharmacists to guide the dose and drug selection for the initial drug of inclusion, and for the prescription of any subsequent drugs that are newly started during follow-up and are on the list of drugs eligible for inclusion in PREPARE (i.e. a DPWG guideline is available for the drug). Physicians and pharmacists are given PGx test results but are not forced to adhere to the DPWG guidelines.

Main study parameters/endpoints:

The primary endpoint of PREPARE is a composite endpoint of clinically relevant drug-genotype associated ADRs. Secondary outcomes include other clinical outcome measures (e.g. total number of ADEs, dose changes, drug cessations etc.), a cost-effectiveness evaluation, and process metrics for implementation; the latter includes physician and pharmacist adherence to the DPWG guidelines, and the acceptance of PGx-informed prescribing to health care professionals and patients.

Nature and extent of the burden and risks associated with participation, benefit and group relatedness:

When a patient chooses to participate in this study, genetic information concerning 48 genetic variants in 13 pharmacogenes will be determined. Burdens to the patient are: 1) the supply of a blood or saliva sample for DNA collection, 2) being contacted four times at regular intervals by a research nurse (at baseline, four weeks and 12 weeks and at the end of the arm's follow-up period), and 3) being asked to complete online surveys (at two weeks, and eight weeks). Patients who endure an ADR which is categorized as an "extreme phenotype" will be asked to provide an additional blood sample within 24 hours of the ADR. In a sub-study, patients included in the study for a first prescription of voriconazole, metoprolol, simvastatin, atorvastatin, fluorouracil or capecitabine will be asked to provide additional blood spot samples at multiple time points. Benefits to patients in the study arm include a potential reduced risk of ADRs. Overall, minimal risks are expected for included patients due to the fact that all of the drugs included within this study have previously been licensed for routine use and thus have been evaluated as having a positive benefit/risk ratio.

TABLE OF CONTENTS

1. INTRODUCTION AND RATIONALE 16
2. STUDY OBJECTIVES 19
2.1. Primary Objective 19
2.2. Secondary Objectives 19
3. STUDY PLAN AND PROCEDURES 20
3.1. Overall study design 20
3.2. Data collection procedures 22
3.2.1. Data collection summary matrix 24
3.2.2. Study arm patient journey 26
1.1.1 Standard of care arm patient journey 27
1.1 DNA sample collection 28
1.2 Randomisation and Blinding 28
3.2.3. Randomisation 28
3.2.4. Blinding 29
3.3. Study timetable 29
4. STUDY POPULATION 30
4.1. Population 30
4.2. Inclusion criteria 31
4.3. Exclusion criteria 32
4.4. Patient recruitment and inclusion 33
4.5. Patient log and screening 34
4.6. Sample size calculation 34
4.7. Withdrawal of individual subjects 35
4.7.1. Specific criteria for withdrawal 35
4.7.2. Replacement of individual subjects after withdrawal 36
4.7.3. Follow-up of subjects withdrawn from treatment 36
5. TREATMENT OF SUBJECTS 36
5.1. Investigational strategy 36
5.1.1. Variant Selection and Platform 36
5.1.2. The DPWG Guidelines 39
5.1.3. “Safety-Code” Card 41
6. ENDPOINT ASSESSMENT 44
6.1. Primary endpoint 44
6.1.1. Severity Classification 45
6.1.2. Causality Assessment 46

6.1.3. Drug-genotype association....Error! Bookmark not defined.
6.2. Procedure of data collection 48
6.3. Secondary endpoints 48
6.3.1. Cost-effectiveness 49
6.3.2. Implementation and adoption of guidelines 49
6.3.3. Drug-Drug-Gene Interaction Sub-study 49
6.3.4. Extreme phenotypes 52
7. STATISTICAL ANALYSIS 53
7.1. Primary analysis....Error! Bookmark not defined.
7.2. Secondary analyses ....Error! Bookmark not defined.
8. ASSESSMENT OF SAFETY 54
8.1. Definition SAEs 54
1.1 Methods for recording and follow-up of ADEs 54
8.2. Reporting of SAEs 54
8.3. Safety Committee 55
8.4. Premature termination of the study 55
9. STRUCTURED RISK ANALYSIS 56
9.1. Potential issues of concern 56
10. DATA HANDLING AND RECORD KEEPING 59
10.1. Electronic Case Report Forms 59
10.2. Data security and subject privacy 60
10.3. Access to source data and record retention 60
10.4. Storage of human material 60
10.5. Data management 60
11. QUALITY CONTROL AND QUALITY ASSURANCE 61
11.1. Monitoring 61
11.2. Direct access to documents 61
12. TEMPORARY HALT AND PREMATURE TERMINATION OF THE STUDY 62
12.1. Temporary halt of study 62
12.2. Premature termination of study 62
13. ETHICS 63
13.1. Ethical conduct of the study 63
13.2. Independent ethics committee 63
1.1 Patient information and informed consent 63
13.2.1. Recruitment in community pharmacies 63
13.2.2. Informed Consent and Patient Brochure 63
13.3. Changes to the study protocol 64

13.4. Annual progress report 64

14. INSURANCE 65

15. STUDY REPORT 65

16. PUBLIC DISCLOSURE AND PUBLICATION POLICY 65

17. REFERENCES 66

18. APPENDICIES 67

18.1. National Coordinators and Principal Investigators 67

18.2. Flow Charts of Patient Journey 68

18.2.1. General Flow Chart of Patient Journey 68

18.2.2. Site Specific Flow Chart of Patient Journey 69

18.3. DPWG Guidelines 71

18.4. Scientific Advisory Board 89

18.5. Organogram of U-PGx Consortium 91

LIST OF ABBREVIATIONS AND RELEVANT DEFINITIONS

ADR	Adverse Drug Reaction
ADE	Adverse Drug Event
ADL	Activities of Daily Living
AE	Adverse Event
AR	Adverse Reaction
CDS	Clinical Decision System
CROA	Centro di Riferimento Oncologico
CV	Curriculum Vitae
DPWG	Dutch Pharmacogenomics Working Group
eCRF	Electronic Case Report Form
EMA	European Medicines Agency
EU	European Union
EQ5D	European Quality of Life – Five Dimensions
GCP	Good Clinical Practice
IB	Investigator's Brochure
IC	Informed Consent
ICH	International Council of Harmonization
LCAT	Liverpool Causality Assessment Tool
LIM	Lareb Intensive Monitoring
LUMC	Leiden University Medical Center
MAF	Minor Allele Frequency
MSC	Medication Safety Code
MUWV	Medical University of Vienna
NCI-CTCAE	Common Toxicity Criteria Adverse Events
PGx	Pharmacogenomic(s)
PHUL	University of Liverpool
PREPARE	PREemptive Pharmacogenomic testing for Preventing Adverse drug REactions
QAC	Quality Assurance Committee
QoL	Quality of Life
(S)AE	(Serious) Adverse Event
SASG	Servicio Andaluz de Salud
SPC	Summary of Product Characteristics
ULMF	University of Ljubljana
UPAT	University of Patras

1. INTRODUCTION AND RATIONALE

Pharmacogenomics is the study of genetic variability affecting an individual's response to a drug. It is one of the first clinical applications of the post-genomic era and allows personalized medicine rather than the established 'one size fits all' approach to prescribing drugs. The expected reduction in 'trial and error' drug prescribing will result in safer and more cost-effective drug therapy.

Indeed, in recent years a number of studies have shown that genetic variation in genes encoding drug transporters and drug metabolic enzymes affect drug disposition, and can lead to under-dosing or overdosing in affected individuals. Furthermore, variation in genes encoding drug targets has also been shown to have a major influence on a drug's effect, influencing both efficacy and safety. The results of five randomized clinical trials strongly indicate that PGx testing prior to prescribing, with subsequent genotype-informed treatment stratification, can be associated with improved patient outcomes (Table 1).

In addition, almost 15% of medicinal products evaluated by the EMA between 1995 and 2014 contain PGx information in their product label (1). PGx-based therapeutic dose and drug selection recommendations have also been created and published by the DPWG (2).

Table 1 Randomized clinical trials supporting PGx testing prior to prescribing with clinical outcome endpoints.

Drug	Endpoint	Genetic variants	Reference
Abacavir	Hypersensitivity	*HLA-B *5701*	*N Engl J Med* 2008 Feb 7;358(6):568-579.
Acenocoumarol/ Phenprocoumon	% of time in therapeutic INR	*VKORC1/CYP2C9*	*N Engl J Med* 2013 Dec 12;369(24):2304-2312.
Warfarin	% of time in therapeutic INR	*VKORC1/CYP2C9*	*N Engl J Med* 2013 Dec 12;369(24):2294-2303.
	Risk of bleeding	*VKORC1/CYP2C9*	*Lancet* 2015 Jun 6;385(9984):2231-2232.
Mercaptopurine/ Azathioprine	Incidence of leukopenia	*TPMT*	*Gastroenterology* 2015 Oct;149(4):907-17.e7.

Importantly, over 95% of the population carry at least one actionable genotype for one of the genes covered by the DPWG guidelines (Figure 1).

However, despite major scientific and clinical advances in PGx and the availability of several

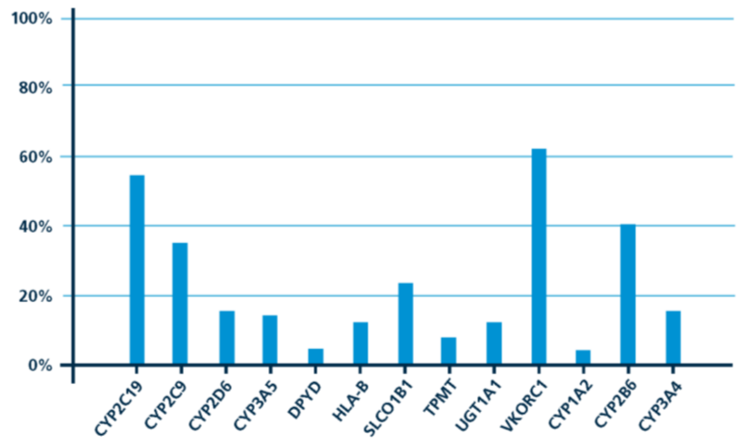

Figure 1 The frequency of actionable pharmacogenes. Over 95% of the population carry at least one actionable genotype for one of the genes covered by the DPWG guidelines (4).

commercially available PGx tests, the application of PGx into routine patient care remains very limited. We have described the barriers for implementation of clinical PGx into routine patient care and recognized the following obstacles (3):

- Lack of guidelines directing the clinical use of PGx test results;
- Lack of selection of a panel of clinically relevant PGx tests;
- Lack of data on diagnostic test criteria (e.g. specificity, sensitivity) of PGx testing;
- Need for training and educating health care professionals and patients about the potential and practicalities of PGx;
- Lack of information on cost-effectiveness and cost-consequences of PGx testing;
- Lack of appropriate ICT technologies and infrastructure to implement routine PGx testing. This requires data storage and clinical interpretation aids, and the incorporation of PGx into the workflow patterns of physicians and pharmacists.

PREPARE will address these major challenges and obstacles for implementing PGx testing into routine care, taking into account the diversity of healthcare systems and citizens across Europe. To achieve successful implementation, PREPARE will provide an extensive training and education programme to prepare recruiting physicians and pharmacists for pre-emptive PGx-based prescribing and dispensing. The effectiveness of this training and education programme will be evaluated by performing pre- and post-programme surveys in the participating countries. Additionally, novel ICT tools will be created to integrate the PGx results into (electronic) medical records before initiation of the study.

Following this, PREPARE will primarily determine if pre-emptive genotyping of an entire panel of clinically relevant PGx markers (for which DPWG guidelines are available: 'pharmacogenes') results in better outcomes for patients (i.e. reduces the number of clinically relevant drug-genotype associated ADRs) and is more cost-effective than standard care. Furthermore, both quantitative and qualitative indicators of implementation success will be monitored and evaluated. In a sub-study, the drug-drug-gene interactions of voriconazole, metoprolol, simvastatin, atorvastatin, fluorouracil and capecitabine will be investigated in more detail by sampling additional plasma blood levels through bloodspots. Finally, explorative data analysis will be performed to associate novel variants identified by NGS techniques with extreme phenotypes, and elucidate novel drug-drug-gene interactions, and so advance the field of PGx.

2. STUDY OBJECTIVES

2.1. Primary Objective

To determine whether the implementation of pre-emptive PGx testing of an entire panel of clinically relevant PGx markers, to guide the dose and drug selection for 39 commonly prescribed drugs, will result in an overall reduction in the number of clinically relevant drug-genotype associated ADRs which are causally related to the initial drug of inclusion (referred to as 'index drug').

2.2. Secondary Objectives

To determine whether the implementation of PGx-guided drug and dose selection: 1) reduces the total number ADRs (causally related to both the index drug and subsequent newly prescribed drugs), 2) reduces the total number of 'serious' ADRs (causally related to the index drug), 3) reduces the number of dose changes for the index drug, 4) reduces the number of drug cessations for the index drug and 5) is cost-effective compared to standard of care. Additionally, the attitudes towards PGx and knowledge of PGx among healthcare providers and patients will be evaluated. Process metrics for implementation among physicians and pharmacists will also be monitored. These metrics include: adherence to DPWG guidelines, and the acceptance of PGx testing. Explorative data analysis will be performed to associate novel variants identified through NGS techniques with extreme phenotypes, and elucidate novel drug-drug-gene interactions, and so advance the field of PGx. Six drug-drug-gene interactions will be investigated extensively by sampling additional plasma blood levels (voriconazole, metoprolol, simvastatin, atorvastatin, fluorouracil and capecitabine) in a sub-study.

3. STUDY PLAN AND PROCEDURES

3.1. Overall study design

This study is a multi-center, open, randomized, cross-over study conducted in seven countries across Europe (Figure 2). The sequence of study and control arm is randomized per country to minimize the influence of time dependent variables. To avoid bias and account for differences between countries, countries will serve as their own controls. The countries will be randomized to start with: 1) implementing pre-emptive PGx testing to guide drug and dose selection (study arm), or 2) providing standard clinical care (control arm). The first time-block will encompass an 19 month enrolling period (and an additional 3 months follow-up) and the second time block will encompass an 21 month enrolling period (and an additional 3 months follow-up), where a new set of patients is recruited in each arm. All patients will be followed up for a minimum of 12 weeks, and up to a maximum of 22 months (first time block), and 21 months (second time block). 4,050 patients in the study arm and 4,050 patients in the control arm will be recruited prospectively in Austria, Greece, Italy, Slovenia, Spain, The Netherlands, and United Kingdom. Each implementation country will focus on, but is not limited to, recruiting patients within a specific therapeutic area.

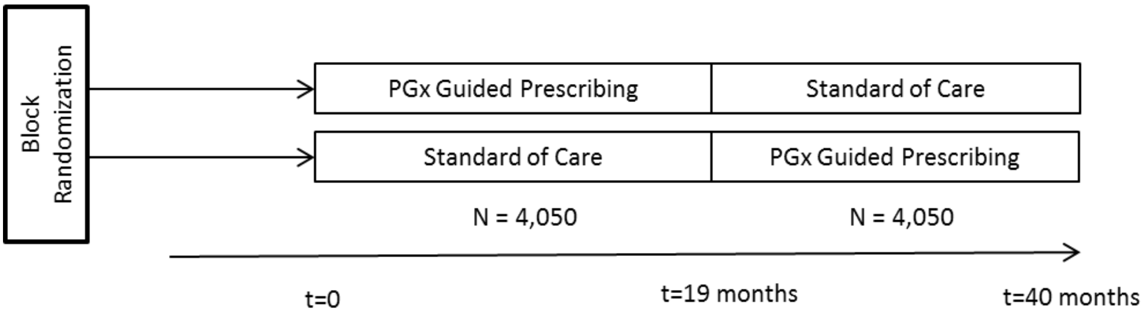

Figure 2 Study design of the multi-center open randomized cross-over trial.

A patient who receives a first prescription for any drug listed in Table 5 (i.e. essentially all drugs that have a DPWG PGx guideline available) will be eligible for inclusion. This is further defined as the 'index drug'. T=0 is defined as the day the patient initiates the index drug. All included patients (both study and control arm patients) will donate DNA by a blood or saliva sample and will be genotyped for our panel of 48 genetic variants in 13 pharmacogenes. Patients in the control arm will receive the usual prescription, without PGx-guided drug or dose selection and a mock "Safety-Code" card (see Section 5.1.3). In the study arm only, a patient's DNA test results will be: 1) recorded in the (electronic) medical record and 2) handed to the patient on a "Safety-Code" card (see Section 5.1.3). PGx results will be returned to the physicians and pharmacists within seven days of initiating the index drug guide the patient's drug and dose selection as per the DPWG guidelines. Health care

providers are not forced to adhere to the DPWG guidelines, but are simply provided with all the tools necessary to implement PGx-guided prescribing. Patients in the study arm may start with the usual prescription, to bridge the lag-time, and will be contacted by their physician or pharmacist if their PGx results recommend a prescription alteration.

Data on ADEs, costs and implementation process parameters will be collected during follow-up (at baseline, four weeks and 12 weeks after initiation of the index drug and a cross-sectional collection at the end of their time block). Data on patient reported outcomes ADEs are collected at two and eight weeks (Figure 3). All patients who receive a subsequent new prescription for another drug during the their time block will be asked to report this to the research nurse. Patients in the study arm will also be asked to present their PGx results (on their “Safety-Code” card) to the prescribing physician or dispensing pharmacist so that they may use these to guide drug and dose selection. It is recognized that these health care providers may be outside the realm of the study and may therefore not have received training in incorporating pharmacogenomics in clinical care. The research nurse will establish whether this newly prescribed drug is on the list of drugs eligible for PREPARE (Table 5). If the drug is indeed eligible, additional data on ADEs, costs and implementation process parameters regarding this drug will be collected. This prescription is further defined as the ‘subsequent drug’ (Figure 3). This data will be used for the secondary analyses.

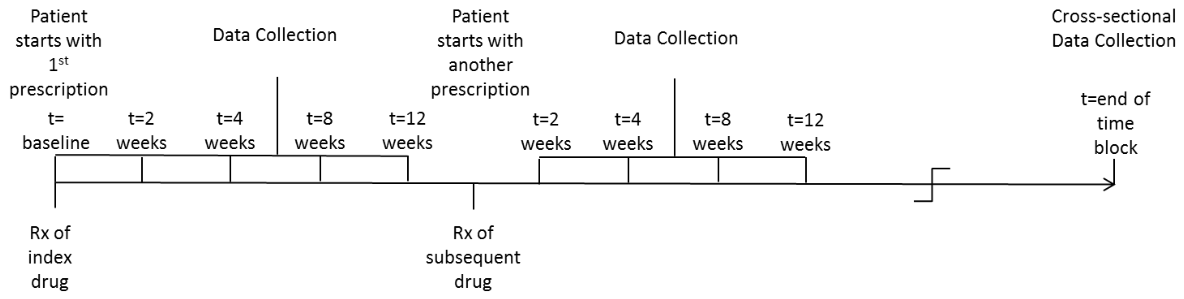

Figure 3 Timeline of data collection

The minimum follow-up time is 12 weeks; the follow-up of all patients recruited in the first time block will cease at the end of the time block in which they were recruited (Figure 3).

PGx test results will be recorded in the patient’s (electronic) medical record and a system will be developed to generate (automated) alerts whenever a relevant drug (listed in Table 5) is prescribed or dispensed to the patient by the physician or pharmacist, respectively. In this way, alerts will be

fully integrated into the regular workflow (prescribing and dispensing) of physicians and pharmacists. Since the genotype results will be recorded in the (electronic) medical record, they can be used for PGx-guided prescribing of subsequent newly started drugs listed in Table 5.

In parallel to this, the DNA of patients who endure an ADE categorized as an "extreme phenotype" during follow-up will be analyzed through NGS sequencing of 150-200 ADME genes, to find new variants associated with this phenotype. Six drug-drug-gene interactions will also be further investigated by sampling additional plasma blood levels through blood spots (voriconazole, metoprolol, simvastatin, atorvastatin, fluorouracil and capecitabine) in a sub-study.

3.2. Data collection procedures

T=0 is defined as the day the patient initiates the index drug. During the follow-up period trained research nurses will contact patients at baseline (t=0) (± one week), four weeks (± two weeks) and 12 weeks (± three weeks), to go through a standardized questionnaire and to collect data on the occurrence of any ADEs (Figures 4 and 5).Inpatient participants will be visited by the research nurse. Outpatient participants will be contacted by telephone. The research nurses will acquire information about the occurrence and severity of all ADEs by asking an open question regarding the occurrence of any ADEs, followed by various specific questions related to the patient's answers. In addition, all patients will be contacted by the research nurse at the end of the time block (± four weeks from the end of each time block) to complete the questionnaire for a final time. Research nurses additionally will collect required lab and or test results from patients' (electronic) medical records. If a patient discontinues the index drug during the 12 week follow-up period, follow-up will continue as initially anticipated.

Complementary to this, patients will be followed up by receiving online surveys at two weeks and eight weeks after initiation of the index drug. Patients are requested to complete the surveys within two weeks. The questions within this survey will be used to assess the occurrence and severity of any ADEs experienced by the patients. These surveys will be filled in by the patients online using the web-based Lareb Intensive Monitoring (LIM) tool (see Section 6.1.1). If patients do not have an email address or are not able to fill in an online survey, then the LIM tool will not be used. This data will be used for secondary analyses. Research nurses will register patients for the LIM tool at baseline. Their study-ID number, e-mail address, date of birth, drug of inclusion and initiation date will be reported in the LIM registration portal. An automated e-mail will be sent to the patient at week two and eight after drug initiation. This e-mail will include a link to the LIM portal. Patients will be asked to log-in to

the LIM portal using their e-mail address and a standard password. Patients will be asked to change this password once they log in. This will give patients access to the LIM surveys.

All patients who receive a subsequent prescription for another drug during follow-up will be asked to report this to the research nurse. For all drugs newly started during the time block which are eligible for inclusion (listed in Table 5), follow-up will be in the same manner that was used to follow-up the index drug. This data will be used for the secondary analyses.

Collected data is summarized in Table 2. All data will be entered into an electronic Case Report Form (eCRF) provided, which will guarantee consistency of data collection procedures. Data will be used exclusively in a pseudonymous fashion and data protection will be guaranteed according to international regulations . An independent Contract Research Organization (CRO) will guarantee data integrity and data homogeneity across all seven implementation countries by risk based monitoring of a random sample of 10% of the collected data (Section 11.1).

Research nurses will collect and store patient contact information (e-mail address, telephone number, name and study-ID number) in a separate, local file (national subject identification code list).

3.2.1. Data collection summary matrix

Table 2 Summary of prospective data collection

All in yellow is ONLY collected in the study arm

	Research Nurse				Lareb Intensive Monitoring	
**Variable Domains**	Baseline	4 weeks	12 weeks	End of Study (cross-sectional at the end of each time-block)	2 weeks	8 weeks
**PATIENTS**						
**Personal Characteristics**						
**1.1 General information**	X					
**1.2 In- and exclusion criteria**	X					
**1.3 Demographics**	X					
**1.4 Recruitment Information**	X					
**1.5 Health behaviours**	X					
**1.6 DNA sample collection**	X					
**1.7.1 PGx testing results**	X					
**1.7.2 Safety-code card**	X					
**1.8.1 Index drug prescription**	X					
**1.8.2 Action ability**	X					
**1.8.3 Index drug prescription change**	X					
**1.8.3.1 Contact information HCP**	X					
**1.8.3.1 Adherence to DPWG guidelines**	X					
**1.9 Control for logistics**	X					
**Clinical Monitoring**						
**3. Comorbidities and allergies**	X	X	X	X		
**4. Co-medication and herbal remedies**	X	X	X	X		
**Nurse Assessment : Clinical Outcome**						
**2.1 General information**	X	X	X	X		
**2.2 Index drug changes**		X	X	X		
**2.3 Drug adherence**	X	X	X	X		
**2.4 Global Health Score**	X	X	X	X		
**2.5 Quality of Life**	X	X	X	X	X	X
**2.6 Attitudes and knowledge**	X		X	X	X	X
**Adverse events**						
**5. Adverse drug events**					X	X
**5.1 Patient perception of ADE**		X	X	X		
**5.2 Healthcare costs associated with ADE**		X	X	X		
**5.3 Identifying the extreme phenotype**		X	X	X		
**5.4 ADE assessment of severity**		X	X	X		
**5.5 ADE assessment of causality**		X	X	X		
**5.6 ADE assessment of drug-genotype association**		X	X	X		
**Those who are defined as 'Extreme Phenotypes' and their controls**						
**Identifying the 'Extreme Phenotype'**		X	X	X		
**Plasma blood level of PGx drug (blood sample)**	At time of serious ADR					
**Those included within the sub study (voriconazole, atorvastatin, simvastatin, capecitabine, fluorouracil and metoprolol)**						
**Routine plasma levels (blood spots)**	At multiple time points – see Table 9					

SUBSEQUENT DRUGS:

When a patient reports a subsequent prescription, and the nurse confirms it is a prescription of a drug of interest, the following will happen:

1. The nurse registers the subsequent index drug in 1.8.. Only in the study arm will 1.8.3 be registered.

2. Register the new index drug with LIM to initiate the 2w and 8w follow-up

3. New follow-up regarding the following "repeated measures" is initiated:

- Index drug changes (repeated measure at 4w, 12w)
  - Drug specific -> always ask
- Global health score (repeated measure – at BL 4w, 12w)
  - Time specific -> only asked if the previous collected global health score is at least 1 week old
- Quality of life (repeated measure – at BL, 4w,12w)
  - Time specific-> only asked if the previous collected quality of life is at least 1 week old

	Research Nurse				Lareb Intensive Monitoring	
**Variable Domains**	Baseline	4 weeks	12 weeks	End of Study (cross-sectional at the end of each time-block)	2 weeks	8 weeks
**PATIENTS**						
**Personal Characteristics**						
**1.1 General information**						
**1.2 In- and exclusion criteria**						
**1.3 Demographics**						
**1.4 Recruitment Information**						
**1.5 Health behaviours**						
**1.6 DNA sample collection**						
**1.7.1 PGx testing results**						
**1.7.2 Safety code card**						
**1.8.1 Index drug prescription**	X (add the subsequent drug)					
**1.8.2 Action ability**	X					
**1.8.3 Index drug prescription change as a result of PGx**	X					
**1.8.3.1 Contact information HCP**	X					
**1.8.3.2 Adherence to DPWG guidelines**	X					
**1.9 Control for logistics**						
**Clinical Monitoring**						
**3. Comorbidities and allergies**	X	X	X			
**4. Co-medication and herbal remedies**	X	X	X			
**Nurse Assessment : Clinical Outcome**						
**2.1 General information**	X	X	X			
**2.2 Index drug changes**		X	X			
**2.3 Drug adherence**						
**2.4 Global Health Score**	X	X	X			
**2.5 Quality of Life**	X	X	X		X	X
**2.6 Attitudes and knowledge**					X	X
**Adverse events**						
**5. Adverse drug events**		X	X		X	X
**5.1 Patient perception of ADE**		X	X			
**5.2 Healthcare costs associated with ADE**		X	X			
**5.3 Identifying the extreme phenotype**		X	X			
**5.4 ADE assessment of severity**		X	X			

**5.5 ADE assessment of causality**	X	X	
**5.6 ADE assessment of drug-genotype association**	X	X	
**Plasma blood level of PGx drug (blood sample)**	At time of serious ADR		

**PHYSICIANS AND PHARMACISTS**	
**PGx knowledge and attitudes**	
**Attitudes**	Before study initiation and after study completion
**Knowledge**	Before study initiation and after study completion
**Adoption of guidelines**	
**Guideline adoption**	Directly after every first and subsequent prescription
**Reason for (not) adopting**	Directly after every first and subsequent prescription

3.2.2. Study arm patient journey

Figure 4 shows a general overview of the patient journey in the study arm. A detailed flowchart of the site-specific patient journey can be found in Appendix 18.2.

Patients in the study arm will start with the usual prescription, to bridge the lag –time before the PGx results are ready, and will be contacted by their physician or pharmacist if their prescription is recommended to be changed due to their PGx test results.

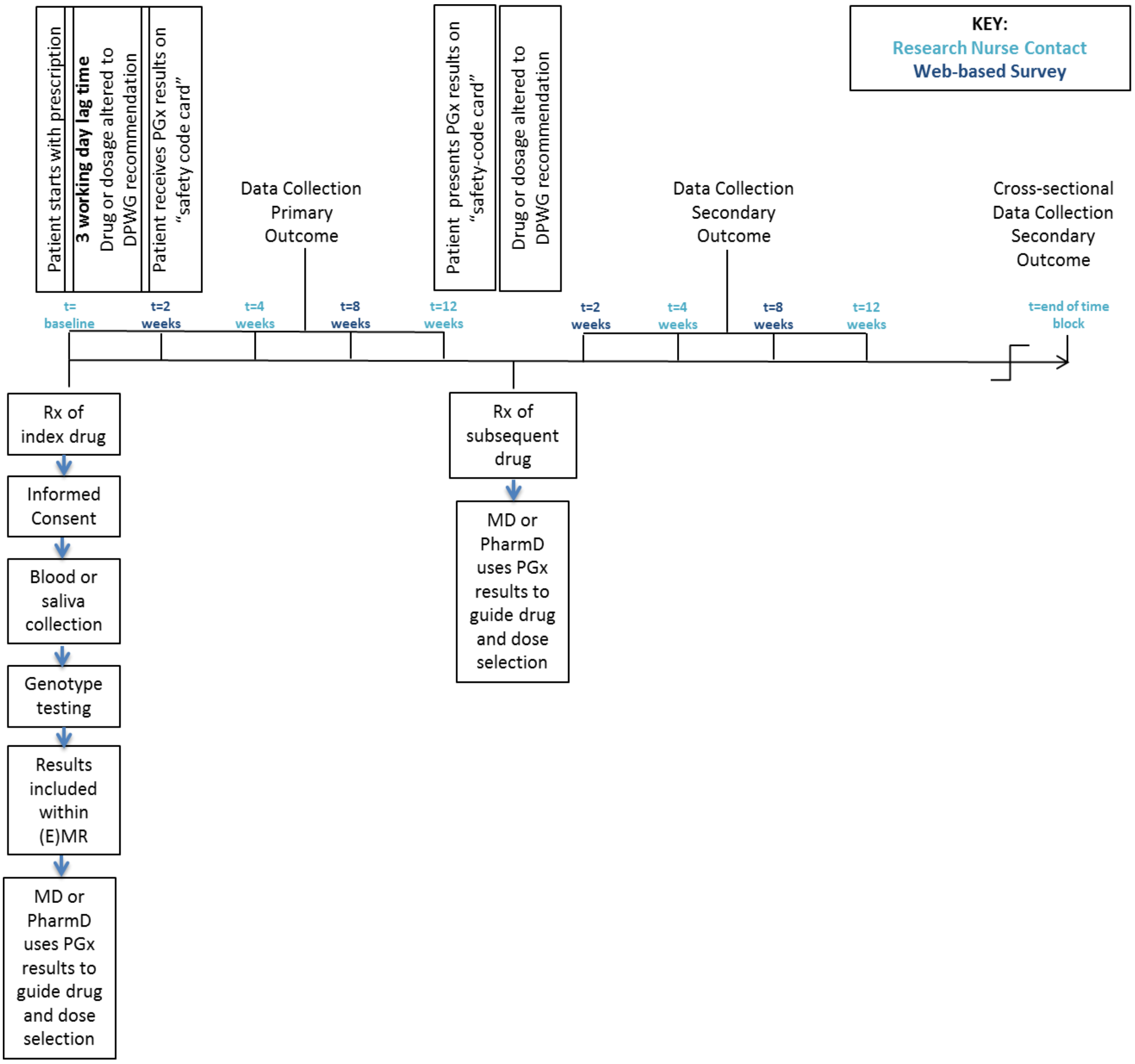

Figure 4 Timeline describing procedures within the study arm

3.2.3 Standard of care arm patient journey

Figure 5 shows a general overview of the patient journey in the standard of care arm. A flowchart of the site-specific patient journey can be found in Appendix 18.2. Genotyping is performed once the respective time-block is completed.

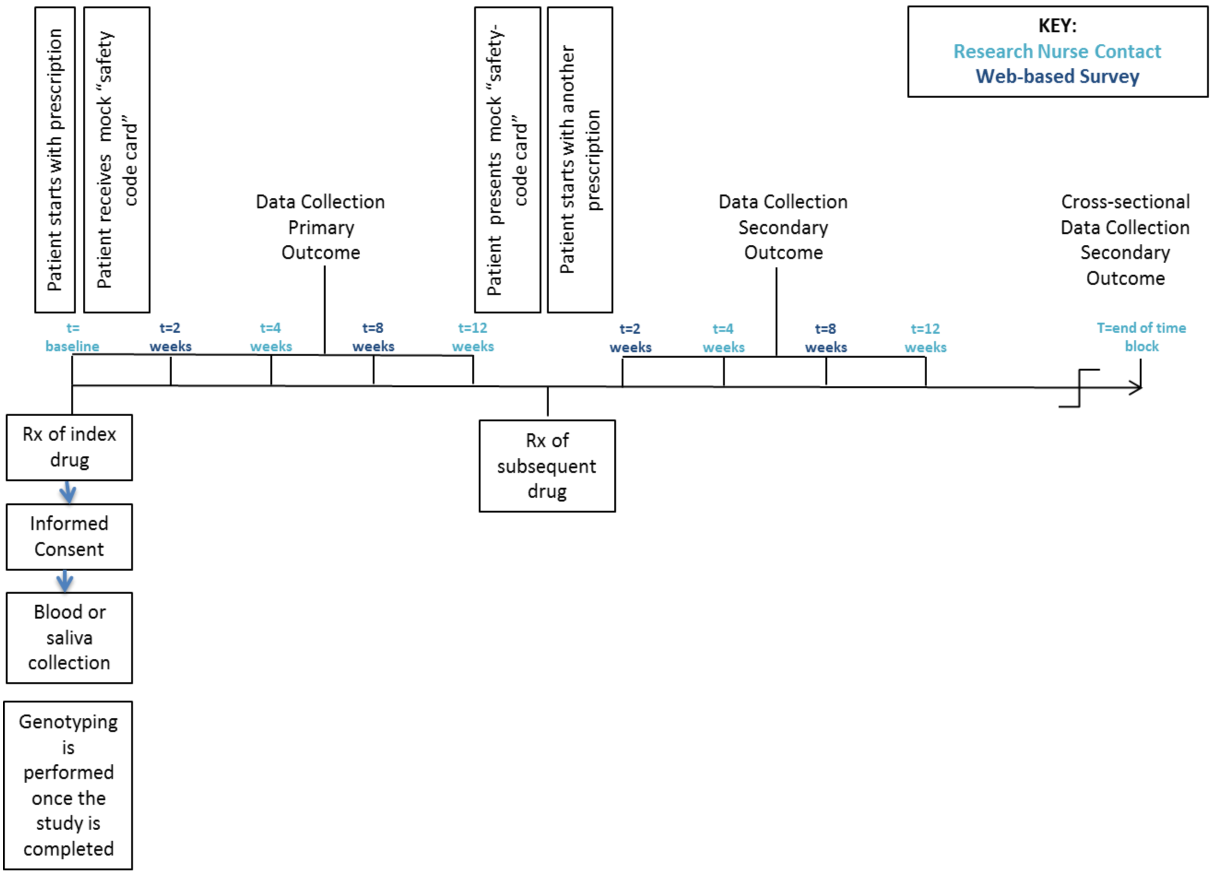

Figure 5 Timeline describing procedures within the standard of care arm

3.3 DNA sample collection

Inpatients will provide a 10 mL blood sample (EDTA) for DNA collection and outpatients will provide a 2mL saliva sample for DNA collection.

Blood samples will be stored at 2-8°C before DNA isolation.

Saliva samples will be collected using the ORAGENE DNA 500 collection kit and stored at room temperature.

3.4 Randomisation and Blinding

3.4.1 Randomisation

The PHUL is unable to start with the study arm due to practical reasons (relocation of the hospital and the genetic laboratory). Therefore, PHUL has been excluded from the randomization and has been assigned to start the study with the standard of care approach. The remaining six sites have

been randomized. Randomization has been performed by medical statistician Dr. Ron Wolterbeek (LUMC) see Table 3 for the results of the randomization.

Table 3 Randomization schedule of 6 study site, excluding PHUL

Site	Starting Strategy
**LUMC**	Standard of care
**SASG**	PGx-guided Prescription
**MUWV**	Standard of care
**UPAT**	PGx-guided Prescription
**ULMF**	PGx-guided Prescription
**CROA**	Standard of care

3.4.2 Blinding

This is an open study. Recruiting physicians/pharmacists cannot be blinded within this study because they must use the patients' PGx results to guide drug and dose selection. Research nurses cannot be blinded within this study because they must manage study logistics. Patients cannot be blinded as those in the study arm as PGx results are available on the "Safety-Code" card.

A 10% random sample of causality and severity assessments of recorded ADEs will be re-assessed by a second independent, blinded (unaware of patient allocation) assessor.

100% of drug-genotype association assessments as per the Dutch Pharmacogenomics Working Group guideline will be assessed by a blinded (unaware of patient allocation) review committee.

3.5 Study timetable

It was anticipated that the study would start in January 1st 2017 and end in December 31st 2019. Due to a delay, the study started on March 6th 2017 and the cross-over was performed on October 1st 2018. The second time-block is extended with a 3 month enrolment period from April 1st 2020 to July 1st 2020.

4. STUDY POPULATION

4.1. Population

Patients of any ethnicity who are at least 18 years old and receive a first prescription for a drug for which a DPWG guideline is available (Table 5), within the realm of routine care, are eligible to participate.

The participating hospitals each will concentrate on, but are not limited to, recruiting within a specific therapeutic area encompassing both in- and out-patient settings (see Table 4):

Table 4 Recruiting centers and their corresponding therapeutic areas of focus

Site	Center name	Therapeutic focus for recruitment	Country/ Language
LUMC	28 community pharmacies	Primary care patients	The Netherlands/ Dutch
	Leiden University Medical Center: Department of Neurology	Neurology in- and outpatients	
SASG	San Cecilio University Hospital: Departments of Pharmacy and Cardiology	will enroll patients with -Patients with unstable angina and acute coronary syndrome patients undergoing percutaneous coronary intervention with stent, patients with peripheral vascular disease and neurology patients	Spain/ Spanish
MUWV	Medical University of Vienna: Division of Nephrology and Dialysis	Patients receiving kidney transplantation	Austria/ German
UPAT	University of Patras Medical School	Psychiatric, oncology and cardiology patients	Greece/ Greek
	Attikon University Hospital in Athens	Psychiatric patients	
	Psychiatric Hospital of Attica in Athens	Psychiatric patients	
	Psychiatric Hospital of Attica in Athens “DROMOKAITEION"	Psychiatric patients	
ULMF	University Medical Centre Ljubljana: Department of Nephrology (Transplantation centre) Department of Abdominal Surgery Department of Neurology	Neurology, renal transplantation and abdominal surgery patients	Slovenia/ Slovenian
	University Psychiatric Hospital Ljubljana (3 divisions)	Psychiatric patients	
	Community Health Centre Ljubljana (8 units)	Primary care patients	
	Community Health Centre Litija	Primary care patients	
	Community Health Centre Kocevje	Primary care patients	
	Institute of Oncology Ljubljana	Oncology Patients	
	University Medical Center Maribor: Department of Psychiatry	Psychiatric patients	
	Sevnica Hospital	General medicine patients and psychiatric patients	
	Community Health Center Domzale	Primary care patients	
	Community Health Center Ivančna Gorica	Primary care patients	
PHUL	University of Liverpool Royal Liverpool University Hospital: Dept. of Molecular & Clinical Pharmacology	. General medicine inpatients.	United Kingdom/ English
	Fulwood Medical Practice	Primary care patients	United Kingdom/ English
	Clatterbridge Cancer Centre	Primary care patients	
CROA	Centro di Riferimento Oncologico, National Cancer Institute in Aviano	Oncology patients	Italy/ Italian

	Centro di Riferimento Oncologico, National Cancer Institute in Treviso	Oncology patients
	Medical Oncology department of the San Filippo Neri Hospital in Rome	Oncology and cardiology patients

4.2. Inclusion criteria

In order to be eligible to participate in this study, a subject must meet all of the following criteria:

1. Subject must be ≥ 18 years old
2. Subject must receive a 1st prescription (meaning no known prescription for this drug in the preceding 12 months) for a drug included in Table 5, which is prescribed to them in routine care.
3. Subject is able and willing to take part and be followed-up for at least 12 weeks
4. Subject is able to donate blood or saliva
5. Subject has signed informed consent

When 10% of total patients on any specific drug have been included in the study or control arm, recruitment of new patients on that drug into that particular arm will no longer be permitted. In other words, the inclusion of patients with a first prescription for the same drug is limited to 5% of the control arm and 5% of the study arm. More specifically, of this 2.5%,in the control arm and 2.5% in the study arm in the first time block, and likewise in the second time block.

Table 5 List of drugs eligible for inclusion (n=39)

Antiarrhythmic	Flecainide
	Propafenon
Analgesic	Codeine
	Tramadol
Anticancer	Capecitabine
	Fluorouracil
	Irinotecan
	Tamoxifen
	Tegafur
Anticoagulation	Acenocoumarol
	Clopidrogel
	Phenprocoumon
	Warfarin
Antidepressant	Citalopram
	Escitalopram
	Paroxetine
	Sertraline
	Venlafaxine
Antidepressant (TCA)	Amitriptyline
	Clomipramine
	Doxepine
	Imipramine
	Nortryptiline

Antiepileptic	Phenytoin
Antihypertensive	Metoprolol
Anti-infective	Efavirenz
	Flucloxacillin
	Voriconazole
Antipsychotic	Aripiprazole
	Haloperidol
	Pimozide
	Zuclopenthixol
Cholesterol-lowering	Atorvastatin
	Simvastatin
Immunosuppressive	Azathioprine
	Mercaptopurine
	Tacrolimus
	Thioguanine
Psychostimulant	Atomoxetine

Table 5 includes all drugs for which an actionable gene-drug interaction is present according to the DPWG recommendations with the exception of abacavir, omeprazole, esomeprazole, lansoprazole, pantoprazole, rabeprazole, oxycodone and oestrogen containing oral contraceptives. Abacavir is excluded because PGx-guided prescribing is part of routine care. Proton pump inhibitors are excluded because they are only associated with a difference in efficacy among aberrant genotypes. The primary outcome of this study is however a reduction in drug-genotype specific ADRs. Oxycodone was initially included as an index drug but removed after the DPWG guideline was updated to be non-actionable. Oestrogen containing drugs will only be included in the study as a subsequent prescription.

4.3. Exclusion criteria

A potential subject who meets any of the following criteria will be excluded from participation in this study:

1. Previous (direct-to-consumer, or clinical) genetic testing for a gene important to the index drug
2. Pregnancy or lactating
3. Life expectancy estimated to be less than three months by treating clinical team
4. Duration of index drug total treatment length is planned to be less than seven consecutive days. A drug whose route of administration changes during the first seven days (e.g. intravenous to oral flucloxacillin) but whose total treatment duration is seven days or longer, is still eligible.
5. For inpatients: hospital admission is expected to be less than 72 hours (to facilitate acting upon the PGX results)
6. Unable to consent to the study
7. Unwilling to take part
8. Subject has no fixed address

9. Subject has no current general practitioner
10. Subject is, in the opinion of the Investigator, not suitable to participate in the study
11. Patient has existing impaired hepatic or renal function for which a lower dose or alternate drug selection are already part of current routine care. This would not apply to any drugs specifically given to manage liver/renal impairment/transplantation.
12. Estimated glomerular filtration rate (MDRD) of less than 15 ml/min per 1,73m^2 in a subject with a functioning graft
13. Patients with advanced liver failure (stage Child-Pugh C)

4.4. Patient recruitment and inclusion

Patients who receive a first prescription for a drug listed in Table 5 will be identified by a 'trigger', and will be eligible for inclusion. Patients who are planned to receive a first prescription for a drug listed in Table 5 are also eligible for inclusion and may be recruited in the study. The time of the first dose of the index drug (t=0) will be noted in the eCRF.

The recruiting physician, pharmacist or nurse will check the in- and exclusion criteria and determine whether the patient is eligible for participation. If so, the patient will be asked if they would like to participate in the study. Patients will be informed about the aims of the study both verbally and in writing. Patients will have the opportunity to ask questions and refuse participation. Patients who consent to participate in the study will sign an informed consent form in duplicate. These forms will be co-signed by the recruiting physician/nurse/pharmacist. One signed consent form will be kept in the Investigator Site File, one given to the patient. Information regarding the informed consent will be noted in the eCRF.

Regarding patients in the study arm:

PGx-results regarding the gene of interest must be reported to the physician/pharmacist within one week of index drug initiation. For example, if a patient starts with the first dose of the index drug on Monday, then the PGx results must be returned to the physician/pharmacist on or before the following Monday. In the meantime, informed consent must be obtained, the DNA sample must be collected and the PGx test must be run. The PGx test will have a turnaround time of three working days. The "Safety-Code card" may be provided after this week. If this is not complied then the patient will be excluded from the data set in a sensitivity analysis.

Regarding patients in the control arm:

Patients must provide informed consent and provide a DNA sample within one week of index drug initiation. For example, if a patient starts with the first dose of the index drug on Monday, then

informed consent and a DNA sample must be provided on or before the following Monday. The mock "Safety-Code card" may be provided after this week. If this is not complied then the patient will be excluded from the data set in a sensitivity analysis.

4.5. Patient log and screening

The recruiting physicians and pharmacists shall note all patients (including their age, sex, index drug and indication) that are considered for participation in their study logbook. Reasons for excluding patients from entering the study should be stated in each case. Reasons given by patients who do not want to participate in the study will be noted in each case. This procedure will be used to assess whether included patients reflect the general target patient population and to establish that the subject population was selected without bias.

4.6. Sample size calculation

The sample size calculation is based on showing a significant reduction of clinically relevant drug-genotype associated ADRs among the subgroup of patients carrying an actionable drug-genotype combination.

The drug-genotype associated ADRs which are derived from the DPWG guidelines are shown in Table 7. All ADRs categorized as grade 2 or higher are considered clinically relevant and combined in the composite endpoint: grade2,3,4,5. To quantify the incidence of drug-genotype associated ADRs listed in Table 7, the EMA frequency classification of ADRs in drug labels was used. For example, an ADR of class 1 is common and has a frequency of ≥ 1/100 to < 1/10 (see Table 7).

Sample size calculation for subgroup of patients carrying an actionable index drug-genotype combination

The frequency of the composite endpoint grade 2,3,4,5 is estimated to be 0.04. We however hypothesize that this frequency is higher among those with an aberrant genotype and could range between 0.04 and 0.10.

We hypothesize that implementing PGx-guided drug and dose selection among those carrying an actionable index drug-gene combination will lead at least a 30% reduction in occurrence of these clinically relevant drug-genotype associated ADRs. If the frequency of the grade 2,3,4,5 endpoint is 0.10 and the true frequency for the carriers of an actionable index drug-gene combination PGx-guided subjects is 0.0683 a sample size of 1,200 subjects in the study arm and 1,200 subjects in the

control is still sufficient to reject the null hypothesis with a probability (power) 0.8 and a Type I error probability of 5%.

Preliminary data from a pilot experiment among 200 patients indicate approximately 30% of included patients will carry an actionable genotype for the index drug. Therefore, 4,000 subjects of unknown genotype in the study arm and 4,000 subjects of unknown genotype in the control arm are needed. To compensate for a 1.25% dropout rate, 100 extra patients will be included in the study. This equals a total inclusion of 8,100 patients, as shown in Table 4.

4.7. Withdrawal of individual subjects

Subjects can leave the study at any time for any reason if they wish to do so without any consequences. The National Principal Investigators can decide to withdraw a subject from the study for urgent medical reasons.

The eCRF will have a specific page that enables the type of withdrawal to be classified precisely, and where possible the individual reason to be documented. Types of withdrawal from which can be selected:

- Complete withdrawal (none of the patient's data can be used, e.g. because the subject wants to fully withdrawal/wrong initial drug etc.)
- Withdrawal from remaining follow-up only (patient consents for the study to use their data which has already been collected, but they decline to continue to be followed-up OR patient did not use index drug for at least 7 consecutive days)
- Lost to follow-up (the subject does not specify that they decline further follow-up, they are unable to be contacted)
- Partial withdrawal (e.g. withdraw from ever using the patient-reported online monitoring system, but happy to be contacted by research team)
- Death

4.7.1. Specific criteria for withdrawal

The subject may be withdrawn from the study if one of the following criteria is met:

- Voluntary discontinuation by the patient, who is at any time free to discontinue their participation in the study
- Incorrect enrolment of the patient
- Patient does not use index drug for a minimum of 7 consecutive days
- Patient experiences adverse events that contraindicate continuing the study (as judged by the National Principal Investigators)
- Patient's general condition contraindicates continuing in the study (as judged by the National Principal Investigators)

4.7.2. Replacement of individual subjects after withdrawal

Since 8,100 patient will be recruited and only 8,000 patients are necessary as calculated by the power calculation, individuals will not be replaced after withdrawal. The withdrawal is not expected to succeed 100 patients.

4.7.3. Follow-up of subjects withdrawn from treatment

If a patient does not attend scheduled visits or replies to attempts of contact, every effort will be made to contact the patient. If a patient wishes to withdraw, the reason for discontinuation will be noted, if the patient agrees. The date and reason (if disclosed) will be noted in the eCRF. How missing data is handled in the analyses is described in the statistical analysis plan.

5. TREATMENT OF SUBJECTS

5.1. Investigational strategy

The intervention in this study is the use of PGx testing to guide drug and dose selection according to the DPWG guidelines. The control arm will receive standard of care, without routine PGx testing. The intervention thus consists of two parts: 1) the panel of pharmacogenes, and 2) the clinical interpretation of the genetic data according to the DPWG guidelines. Physicians and pharmacists are not forced to adhere to the DPWG guideline, but are simply provided with all tools necessary to implement PGx-guided prescribing and dispensing. In order to facilitate implementation of this intervention, physicians and pharmacists will receive education on PGx through an extensive e-learning study initiation of the study arm.

5.1.1. Variant Selection and Platform

The pre-emptive PGx test consists of a panel of 50 PGx SNPs (=48variants) within 13 pharmacogenes. The panel of pharmacogenes includes the variants listed in Table 6 and originates from the DPWG guidelines (1). The criteria for variant inclusion were:

- A DPWG guideline with therapeutic recommendations for the specific genotype exists
- The effect of the variant on the gene is established (e.g. Is the enzyme inactive if the variant is present?)
- Is the overall MAF[1] ≥ 1%?
- If not, is the MAF in selected populations (European/Asian/African) [2] ≥ 1%?
- If the MAF is below 1% in all cases, selection of certain variants is possible if at least one of the implementation sites already determines the allele in patient care.

1. MAF = minor allele frequency. For the determination of the MAF we used www.ensembl.org and received input from Karolinksa Institutet and the DPWG. A MAF of 1% or greater is considered to be common. We restricted allele selection above this selected MAF cut off.

2. The selected populations are the most common populations in Europe. Besides the Europeans, Asians and Africans are present in Europe due to migration.

Variants included in the panel will be updated regularly, in parallel to the development of new guidelines by the DPWG. See Section 5.1.2.

All patients included in the study will be asked to provide informed consent for additional NGS sequencing of the most relevant regions of 150-200 ADME genes (i.e. genes involved in the metabolism and distribution of drugs) with respect to the drug treatment in question, encompassing 6-10 Mb of sequence. The chance of finding secondary findings is negligible within ADME genes. This data will only be used for exploratory analysis and not be implemented in clinical care or returned to the patient.

Table 6 The panel of pharmacogenes and respective variants (RS number included) selected. All patients enrolled in PREPARE will be tested for these variants.

Genes	Allele	Major Nucleotide Variation	dbSNP RS ID
**CYP2B6**	*6/*9	516G>T	rs3745274
**CYP2B6**	*4/*16	785A>G	rs2279343
**CYP2B6**	*18	983T>C	rs28399499
**CYP2B6**	*5	1459C>T	rs3211371
**CYP2C9**	*2	430C>T	rs1799853
**CYP2C9**	*3	1075A>C	rs1057910
**CYP2C9**	*5	1080C>G	rs28371686
**CYP2C9**	*11	1003C>T	rs28371685
**CYP2C19**	*2	681G>A	rs4244285
**CYP2C19**	*3	636G>A	rs4986893
**CYP2C19**	*4A/B	1A>G	rs28399504
**CYP2C19**	*5	1297C>T	rs56337013
**CYP2C19**	*6	395G>A	rs72552267
**CYP2C19**	*8	358T>C	rs41291556
**CYP2C19**	*9	431G>A	rs17884712
**CYP2C19**	*10	680C>T	rs6413438
**CYP2C19**	*17	-806C>T[3]	rs12248560
**CYP2D6**	*xN	Gene duplication or multiplication	X
**CYP2D6**	*3	2549delA	rs35742686
**CYP2D6**	*4	1846G>A	rs3892097
**CYP2D6**	*5	Gene deletion	X
**CYP2D6**	*6	1707delT	rs5030655
**CYP2D6**	*8	1758G>T	rs5030865
**CYP2D6**	*9	2615delAAG	rs5030656
**CYP2D6**	*10	100C>T	rs1065852
**CYP2D6**	*14A/B	1758G>A	rs5030865
**CYP2D6**	*17	1023C>T	rs28371706
**CYP2D6**	*41	2988G>A	rs28371725
**CYP3A5**	*3	6986A>G	rs776746
**CYP3A5**	*6	14690G>A	rs10264272
**CYP3A5**	*7	27131_27132insT	rs41303343
**DPYD**	*2A	IVS14 + 1G>A (1905+1G>A)	rs3918290
**DPYD**	*13	1679T>G	rs55886062
**DPYD**	X	2846A>T	rs67376798
**DPYD**	X	1236G>A	rs56038477
**F5**	X	1691G>A	rs6025
**HLA-B**	*5701	T>G	rs2395029

**NUDT15**	*3	7973C>T	rs116855232
**NUDT15**	*6	55_56insGAGTCG	rs869320766
**NUDT15**	*9	49delGGAGTC	rs746071566
**SLCO1B1**	*5/*15/*17	521T>C	rs4149056
**TPMT**	*2	238G>C	rs1800462
**TPMT**	*3B	460G>A	rs1800460
**TPMT**	*3C	719A>G	rs1142345
**UGT1A1**	*6	211(G>A)	rs4148323
**UGT1A1**	*27	686(C>A)	rs35350960
**UGT1A1**	*28/*37	A(TA)6TAA>A(TA)7TA A/A(TA)8TAA	rs8175347
**VKORC1**	X	1173C>T (C6484T)	rs9934438

All implementation sites will be provided with an identical genotyping platform so that samples can be analysed locally but in a standardised manner. To reflect the different backgrounds of local infrastructure and expertise, centres will be offered a suitable implementation process including staff training. Scaling and batch effects will be accounted for and test results will have a turnaround time of three working days.

The SNPline workflow, by LGC Group will be used to perform all genotyping assays. (See https://www.lgcgroup.com/products/genotyping-instruments/snpline/#.V_Ni1fl95aQ for specifications)

5.1.2. The DPWG Guidelines

In 2005, the KNMP established the DPWG with the objective to develop PGx-based therapeutic recommendations based on a systematic review of the literature. The DPWG consists of 12 members including clinical pharmacists, a general practitioner, physicians, clinical chemists, epidemiologists and a toxicologist. Currently, the database consists of 80 gene -drug-combinations comprising 13 genes.

Briefly, for development of the DWPG guidelines, clinical effects of different severities observed in patients with aberrant genotypes were classified on a seven-point scale derived from the National Cancer Institute's Common Toxicity Criteria (5). These clinical endpoints are ranked from non-significant changes classified as AA (lowest impact), through clinical effects of increasing severity up to the most extreme phenotypes (e.g. death) that are classified as F (highest impact) (Table 7). For each of the drugs included in the DPWG guidelines, specific drug-genotype associated ADRs (i.e. QTc prolongation related to the use of amitriptyline, or leukopenia related to the use of azathioprine) or other effects are systematically categorized into their clinical effect class (i.e. AA to F - Table 7).

Table 7 For development of the DWPG guidelines clinical effects observed in patients with aberrant genotypes were classified on a seven-point scale derived from the National Cancer Institute's Common Toxicity Criteria (CTC AE).

Class (Grade NCI-CTCAE)	EMA ADE frequency	Clinical Effect
AA#		Positive clinical effect (NS)
AA		Clinical effect (NS): no change or a non-significant change of clinical parameters<br>Kinetic effect (NS): no change or a non-significant change of kinetic parameters.
A	Common ≥ 1/100 to < 1/10	Minor clinical effect (S): QTc prolongation (<450 ms ♀, <470 ms ♂); QTc time increase < 60ms; INR increase < 4.5<br>Kinetic effect (S): significant change of kinetic parameters
B (Grade 1)	Common ≥ 1/100 to < 1/10	Clinical effect (S): short-lived discomfort (< 48 hr) without permanent injury: e.g. reduced decrease in resting heart rate; reduction in exercise tachycardia; decreased pain relief from oxycodone; ADE resulting from increased bioavailability of atomoxetine (decreased appetite, insomnia, sleep disturbance, depressive mood, etc); neutropenia > 1.5x10^9/l; leucopenia > 3.0x10^9/l; thrombocytopenia > 75x10^9/l; moderate diarrhea not affecting daily activities; reduced glucose increase following oral glucose tolerance test; muscle complaints creatine kinase <3 times normal upper limit
C (Grade 2)	Common ≥ 1/100 to < 1/10	Clinical effect (S): long-standing discomfort (48-168 hr) without permanent injury e.g. failure of therapy with tricyclic antidepressants, atypical antipsychotic drugs; extrapyramidal side effects; parkinsonism; ADE resulting from increased bioavailability of tricyclic antidepressants, metoprolol, propafenone (central effects e.g. dizziness); increased INR 4.5-6.0; neutropenia 1.0-1.5x10^9/l; leucopenia 2.0-3.0x10^9/l; thrombocytopenia 50-75x10^9/l; muscle complaints creatine kinase 3-10 times normal upper limit
D (Grade 3)	Uncommon ≥ 1/1,000 to< 1/100	Clinical effect (S): long-standing discomfort (> 168 hr), permanent symptom or invalidating injury e.g. failure of prophylaxis of atrial fibrillation; venous thromboembolism; decreased effect of clopidogrel on inhibition of platelet aggregation; ADE resulting from increased bioavailability of phenytoin; INR > 6.0; neutropenia 0.5-1.0x10^9/l; leucopenia 1.0-2.0x10^9/l; thrombocytopenia 25-50x10^9/l; severe diarrhea; myopathy (muscle complaints creatine kinase ≥10 times normal upper limit)
E (Grade 4)	Uncommon ≥ 1/1,000 to< 1/100	Clinical effect (S): Failure of lifesaving therapy e.g. anticipated myelosuppression; prevention of breast cancer relapse; arrhythmia; neutropenia < 0.5x10^9/l; leucopenia < 1.0x10^9/l; thrombocytopenia < 25x10^9/l; life-threatening complications from diarrhea; rhabdomyolysis
F (Grade 5)	Rare ≥ 1/10,000	Clinical effect (S): death; arrhythmia; unanticipated myelosuppression

NS: not statistically significant difference; S: statistically significant difference; INR: international normalized ratio; EMA: European Medicines Agency; ADE: adverse drug event.

Before initiation of this study, the DPWG guidelines will be translated into all local languages to facilitate interpretation by local physicians and pharmacists. The guidelines will be maintained and updated regularly to ensure all available scientific information is included and recommendations are based on the latest literature. The DPWG will update each gene-drug interaction every two years. The

DPWG also expects to develop four new gene drug-interactions per year. These drugs will not be added to the list of drugs for inclusion (Table 5), as this would result in a difference in the study and control arms. These guidelines will still be available for physicians and pharmacists once the study is completed. Additionally, the CPIC guidelines can also be consulted by physicians and pharmacists through the www.PharmGKB.org. These too are updated regularly and will remain available once the study is completed. The original version of the DPWG guidelines are attached in Appendix 18.3. DPWG guidelines are periodically updated and these changes are directly implemented in the study protocol. As of February 20th 2018, the DPWG guideline for oxycodone was updated. The updated version no longer had an actionable recommendation for any phenotype. As a result, oxycodone was removed as an index drug as per May 15th 2018.

5.1.3. "Safety-Code" Card

Patients in the study arm will receive their PGx results on a "Safety-Code" card (see Figure 6 and Figure 7). This card is part of a mobile-based clinical decision support system called the 'Medication Safety Code' (MSC) system that is independent of existing IT infrastructures, and enables the quick retrieval of patient-relevant PGx drug dosing guidelines. The MSC system does not require central patient data storage. Instead, the "Safety-Code" card contains a QR code that stores the patient's encoded PGx results. It can be decoded and interpreted by common smartphones and other devices. After scanning the QR code, the medical professional is led to a website that provides drug dosing recommendations customized to the PGx profile of the patient.

In the context of PREPARE, the MSC system is aimed to serve as an auxiliary tool to maximize the accessibility and sharing of PGx results within and between different health care settings and health care professionals. Patients will be asked to show their "Safety-Code" card to physicians and pharmacists who prescribe or dispense drugs to them during the follow-up period of the study. These providers can thus use the patient's PGx results to guide drug and dose selection. Concomitantly, patients will be asked to report additional drug prescriptions to the research nurse during the follow-up period.

The "Safety-Code" card will include the following information:

- Study patient ID number
- Phenotypic PGx results
- List of drugs that are critical for the card owner, and for which a DPWG guideline is available (Table 5)

- QR code which will direct the physician to the DPWG guidelines applicable for the patients results
- URL which will direct the physician to the DPWG guidelines applicable for the patients results
- Patient name and date of birth
- PREPARE research team contact details

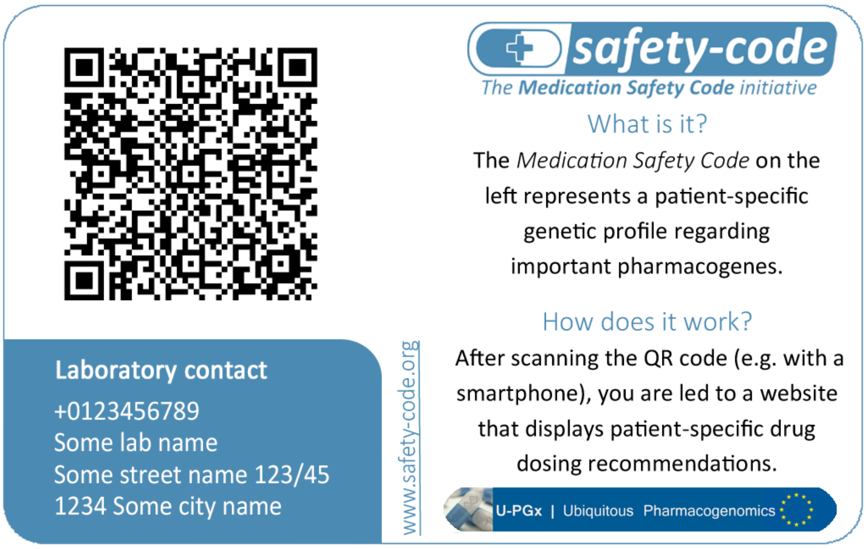

Figure 6 Front of the 'Safety-Code' card

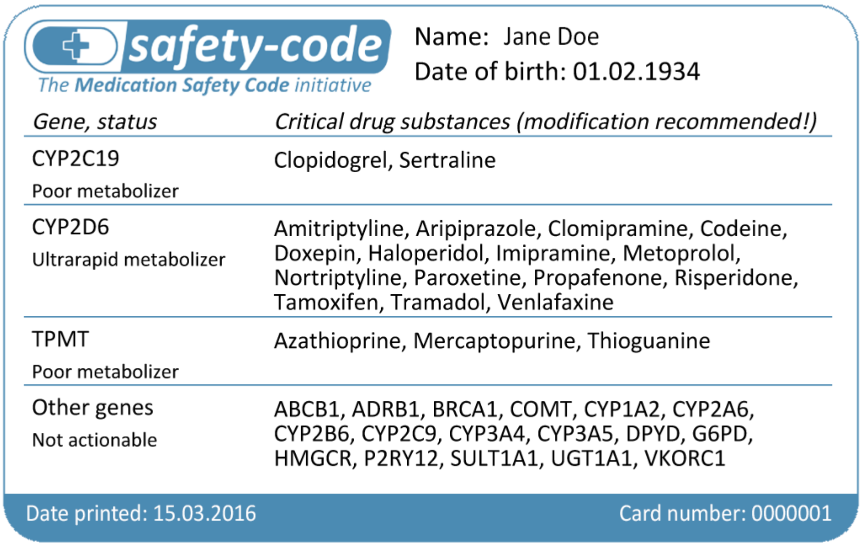
safety-code
The Medication Safety Code initiative

Name: Jane Doe
Date of birth: 01.02.1934

Gene, status	*Critical drug substances (modification recommended!)*
CYP2C19<br>Poor metabolizer	Clopidogrel, Sertraline
CYP2D6<br>Ultrarapid metabolizer	Amitriptyline, Aripiprazole, Clomipramine, Codeine, Doxepin, Haloperidol, Imipramine, Metoprolol, Nortriptyline, Paroxetine, Propafenone, Risperidone, Tamoxifen, Tramadol, Venlafaxine
TPMT<br>Poor metabolizer	Azathioprine, Mercaptopurine, Thioguanine
Other genes<br>Not actionable	ABCB1, ADRB1, BRCA1, COMT, CYP1A2, CYP2A6, CYP2B6, CYP2C9, CYP3A4, CYP3A5, DPYD, G6PD, HMGCR, P2RY12, SULT1A1, UGT1A1, VKORC1

Date printed: 15.03.2016 Card number: 0000001

Figure 7 Back of the 'Safety-Code' card

Patients in the control arm will also receive a mock safety code card. This card will not contain any PGx information, but only include a list of drugs of interest to the study (Table 5) and PREPARE research team contact details.

Local card logistics

The mock “Safety-Code” card will be provided to patients once they have provided the DNA sample. The “Safety-Code” card including the patient’s PGx results will be provided to patients once PGx results have been provided to the treating physician/pharmacist.

Study completion and card disposal

At the end of the study arm the participating patients in the study arm will have the choice to keep or dispose of the card. If they wish to dispose of the card, they will be asked to return the card and it will be destroyed. If they wish to keep the card, they may continue to use it in clinical care.

Participants in the control arm may receive their “Safety-Code” card once the study is completed. They are able to opt-in for this option during the informed consent and may change their mind at any time. Again, they may continue to use it in clinical care once the study is completed.

6. ENDPOINT ASSESSMENT

6.1. Primary endpoint

The primary outcome is the occurrence of at least one causal (definite, probable or possible), clinically relevant (classified as NCI-CTCAE grade 2, 3, 4, or 5), attributable to the index drug, within 12 weeks of follow-up. For oncology patients receiving 5-FU, capecitabine, tegafur or irinotecan, only hematological toxicities of NCI-CTCAE grade 4-5 and non-hematological toxicities of NCI-CTCAE grade 3-5 will be considered clinically relevant.

All collected ADEs during the follow-up period will be assessed with regard to causality, severity and the association with genotype (Figure 8). The severity of the ADE is classified using the CTCAE classification scale (5) for primary analysis and the WHO classification (serious/non-serious) for secondary analysis. Following this, a causality assessment (Section 6.1.2) will be performed using the Liverpool Causality Assessment Tool (LCAT) (6). Once an ADE has been assessed regarding causality it is referred to as an ADR.. Only clinically relevant adverse events, categorized as definitely, probably or possibly caused by the drug (i.e. now constituting an ADR), will contribute to the primary endpoint, as highlighted in green in Figure 8.

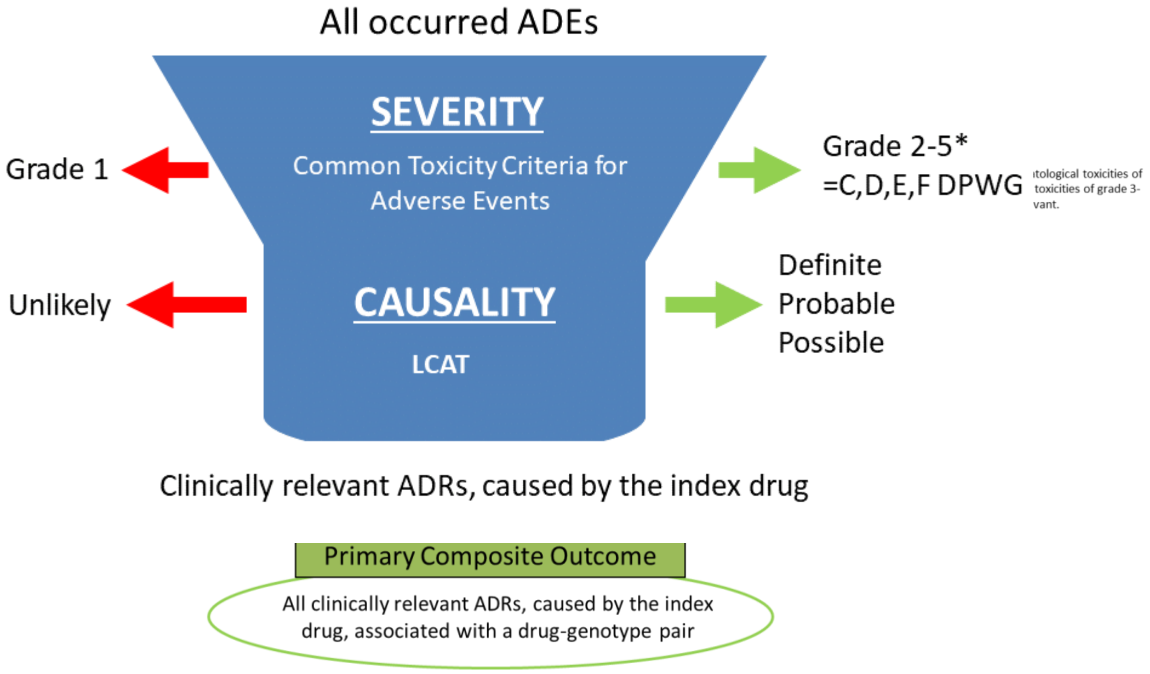

*Figure 8 Funnel plot of classification of ADEs which contribute to the primary endpoint.*For oncology patients only hematological toxicities of grade 4-5 and non-hematological toxicities of grade 3-5 will be considered clinically relevant. LCAT: Liverpool Causality Assessment Tool*

6.1.1. Severity Classification

All ADEs will be classified using both the National Cancer Institute's Common Toxicity Criteria for Adverse Events (NCI-CTCAE) scale (primary outcome) and the WHO classification (secondary outcomes).

The NCI-CTCAE scale is a descriptive terminology that can be utilized for ADE reporting. A grading (severity) scale is provided for each ADE term. The current CTCAE (version 4.0) consists of 790 individual ADE items, which often occur as a result of cancer treatment. Each item represents a discrete event which is graded for severity on a five point scale based on clinical criteria. Since this study also measures ADEs outside the realm of cancer treatment the ADEs which cannot be classified using the NCI-CTCAE (such as bleeding events) will be classified using the pre-specified DPWG classification as listed in Table 7. In fact the DPWG classification is an expanded version of the CTCAE including all ADRs identified by the DPWG during the systematic literature review of gene-drug pairs. If an ADE occurs which cannot be classified using either of these scales, the ADE will be classified using the general definition of the NCI-CTCAE as described in Table 8.

Table 8 Equivalence of DPWG ADR classification and CTCAE classification

DPWG Classification	CTCAE	General definition of the CTCAE
**A**	-	-
**B**	Grade 1	Mild; asymptomatic or mild symptoms; clinical or diagnostic observations only; intervention not indicated.
**C**	Grade 2	Moderate; minimal, local or non-invasive intervention indicated; limiting age-appropriate instrumental activities of daily living*.
**D**	Grade 3	Severe or medically significant but not immediately life-threatening; hospitalization or prolongation of hospitalization indicated; disabling; limiting self-care activities of daily living**.
**E**	Grade 4	Life-threatening consequences; urgent intervention indicated.
**F**	Grade 5	Death related to AE.

Activities of Daily Living (ADL) *Instrumental ADL refer to preparing meals, shopping for groceries or clothes, using the telephone, managing money, etc. **Self-care ADL refer to bathing, dressing and undressing, feeding self, using the toilet, taking medications, and not bedridden.

NCI-CTCAE version 4.0 will be used by clinicians and research nurses to grade severity of all ADEs reported at baseline, four weeks, 12 weeks and at the end of the time block (cross-sectional). If data from patients' medical records are be used to classify severity, this data will be recorded in the eCRF. The methods used by the assessors will be described in a Standard Operating Procedure (4.9).

10% of severity assessments will be reassessed by Lareb (www.lareb.nl) to independently validate the severity assessments. These assessors will be blinded to patient's arm allocation.

For secondary analyses the Lareb Intensive Monitoring (LIM) tool will be used to measure the frequency and severity of patient reported ADEs at two weeks and eight weeks. The LIM tool is a web-based intensive monitoring system and has previously been validated in several clinical trials as a feasible and accurate method to collect adverse drug event data (7, 8). The LIM surveys will be translated to local languages before study initiation.

The WHO classifies (secondary analysis) SAEs as:

An adverse event is any untoward medical occurrence or effect that

- results in death;
- is life threatening (at the time of the event);
- requires hospitalisation or prolongation of an existing inpatient hospitalisation;
- results in persistent or significant disability or incapacity;
- is a congenital anomaly or birth defect; or
- is any other important medical event that did not result in any of the outcomes listed above due to medical or surgical intervention but could have been based upon appropriate judgement by the Principal Investigator.

An elective hospital admission will not be considered a SAE. Any ADE which is not classified as being serious is classified as being non-serious.

6.1.2. Causality Assessment

The Liverpool Causality Assessment Tool has been developed by the University of Liverpool. It assigns the full range of causality categories and has demonstrated a good inter-rater reliability (6). The tool is shown in Figure 9. Once ADEs are assessed with regard to causality they are referred to as ADRs. Only ADRs classified as definitely, probably or possibly will contribute to the primary endpoint. The methods used by the assessors will be described in a Standard Operating Procedure (4.8).

A health care professional assigned by the National Principal Investigators will perform the causality assessments. To reduce inter-observer variability, providers performing the causality assessments will receive a two-hour training session, provided by the Royal Liverpool Hospital to ensure similar assessments.

10% of causality assessments will be reassessed by Lareb (www.lareb.nl) to independently validate the causality assessments, once data collection is complete. These assessors will be blinded to patient's arm allocation.

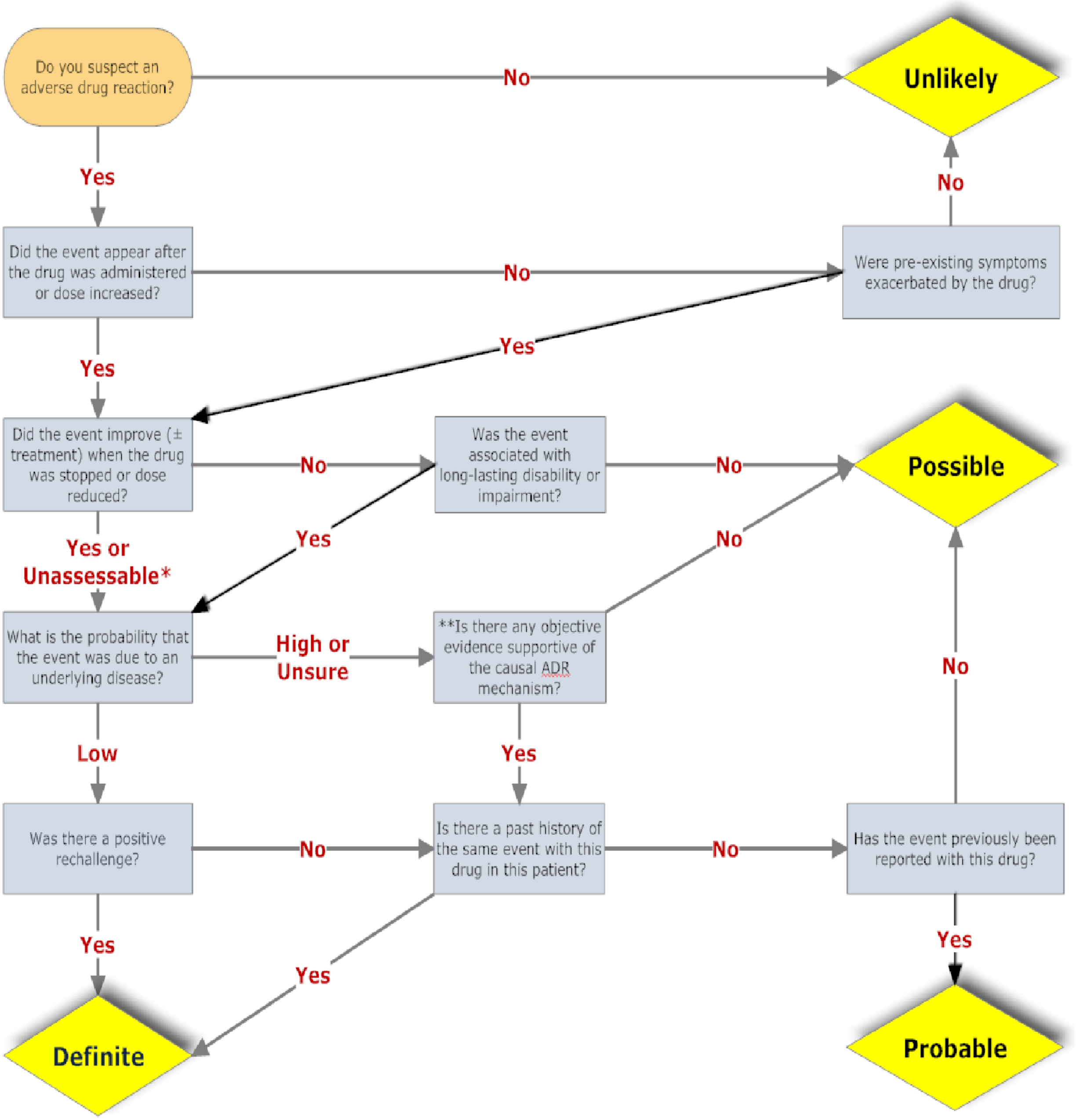

*Unassessable refers to situations where the medicine is administered on one occasion (e.g. Vaccine), the patient receives intermittent therapy (e.g. Chemotherapy), or is on medication which cannot be stopped (e.g. Immunosuppressants)

** Examples of objective evidence: positive laboratory investigations of the causal ADR mechanism (not those merely confirming the adverse reaction), supra-therapeutic drug levels, good evidence of dose-dependent relationship with toxicity in the patient

Figure 9 Flow-chart of Liverpool Causality Assessment Tool

6.2. Procedure of data collection

Patients must be contacted by the research team for data collection within the time window as described in section 3.2. This is purely to manage logistical and practical data collection issues. However, both patients and physicians may report ADEs outside these time windows, by contacting the research team (for example in the case of serious ADEs). In both cases (contact by the research team in the time window or contact by the patient or physician outside the time window), the start date (and end date) of each reported ADE must be entered into the ADE record (chapter 5.1). Additionally, if the research team notices an ADE in the (electronic) medical record which was not reported by either patients or physicians, the research team may enter this as an ADE in the eCRF.

The results of additional tests (e.g. leukocyte count) may be necessary to be able to classify the severity of the ADE according to the NCI-CTCAE. These tests will not be additionally performed if they have not been performed within the realm of clinical care. Results of available tests will be collected from the medical record by the research nurse. These test results will be recorded in the eCRF.

6.3. Secondary endpoints

The secondary analyses of PREPARE are aimed at evaluating clinical outcome, cost-effectiveness, quantitative and qualitative indicators of successful implementation strategies, finding novel variants associated with extreme phenotypes and elucidating drug-drug-gene interactions. These will be collected during follow-up as stipulated in Table 2.

Baseline demographics and clinical monitoring:

- Demographic measurements
- Current and past medical history
- Smoking status
- Alcohol consumption
- Self-perceived level of exercise
- Existing drug allergies
- Comorbidities
- Global Health Score
- Co-medication
- Renal function
- Liver function

Secondary clinical outcome:

- Total number of ADEs (related to index and subsequent drugs)
- Dose adjustments to index drug
- Drug cessation (and reason for discontinuation)
- Additional drugs that are prescribed during follow-up
- Routine drug levels (only those that are collected routinely) as a proxy for exposure
- Patient-reported drug adherence

6.3.1. Cost-effectiveness

- Quality of life
- Measurement of effectiveness (QALY)
- Total cost estimation (healthcare consumption, cost of drugs, cost of hospitalization, cost of ADEs, cost of genetic testing etc.)

6.3.2. Implementation and adoption of guidelines

Among patients:

- Beliefs, knowledge and usability of PGx

Among implementing physicians and pharmacists:

- Process indicators for implementation (e.g. guideline compliance, extent of adoption, number of tests)
- Effectiveness of the training and education programme will be evaluated by performing pre- and post-programme surveys in the participating countries
- When health care professionals do not comply with the DPWG guidelines, they will be asked to report the reason(s) for not doing so.

A generic trigger in the electronic prescribing system will alert the physician or pharmacist that PGx results are available in the patient's medical record. When prescribing (or dispensing), the software will ask the prescribing physician or dispensing pharmacist to write whether they complied with the DPWG guidance, and if not, why not.

A sub-analysis will be performed among physicians and pharmacists who did receive extensive pharmacogenomics training before initiation of the study, and those who did not. Additionally the adherence of physicians will be compared to those of pharmacists.

6.3.3. Drug-Drug-Gene Interaction Sub-study

Through a systems pharmacology approach, PGx and non-genetic determinants of drug response (such as gender, age, and concomitant drug intake) are integrated to create novel, powerful and practice-oriented models of personalized medicine. This work will strive toward assessing the relative contribution of PGx to the variability in drug response by utilizing pharmacometric models that integrate PGx with other sources of variability. The models will describe the events from dose to the drug response, thus including effects of PGx on pharmacokinetics and pharmacodynamics.

Population and physiologically-based pharmacokinetic models will be utilised to predict the impact of genetic variability on pharmacokinetic and pharmacodynamic variables.

The aim of this sub-study is to collect multiple drug levels per patient at different time points, as well as measurements of the relevant pharmacodynamic/clinical endpoints. The models developed will be applied to the assembled data (drug levels, endpoints, demographic information, PGx, and non-genetic factors) to elucidate the agreement of model predictions with the observed real-world data (i.e. model qualification). If sufficient amount of data (drug levels, endpoints) will be collected in this sub-study this approach also allows to assess population pharmacokinetic parameters of the target drug in the study population, to further investigate the sources of variability and to potentially improve models already developed.

Therefore, patients included in the primary study for a first prescription of voriconazole, metoprolol, simvastatin, atorvastatin, fluorouracil or capecitabine are eligible to participate in the sub study and will be asked to provide additional blood samples.
Patients will need to provide informed consent for this sub-study before additional blood sampling can occur. Patients may choose not to provide consent for the sub study and still participate in the general study.

A population phamacokinetic sampling design will be used, i.e. a limited number of blood samples will be drawn from subjects at various time points following drug administration. Four to five blood samples for each patient will be collected using the dried blood spots (DBS) method at one day. The number of samples to be collected from each patient depends on the target drug (for details see Table 9). Five sample collection windows will be used as followed: t_0=pre-dose, t_1=0.5-1.5, t_2=2-4, t_3=5-8 and t_4=9-12 hours after a medication intake. In addition, patients will be asked to provide a blood sample within 24 hours after a serious ADR. For each blood sample, descriptive data, including sample ID, collection time window, target drug and dosage as well as dose history must be documented. The dose history contains information about the last (when applicable, at least three and up to five) previous drug administrations, including dose amounts as well as route, date and time of drug administrations. Clinical endpoint data, demographic information as well as clinically relevant drug-drug interactions will be extracted from the general study (PREPARE).

The dried blood samples can easily be collected using a Whatman Protein Saver Card, which holds a maximum of 80uL of blood per spot. Blood samples must be wrapped and stored at -20oC. The

advantage of using DBS, compared with venipuncture, includes the relative ease and low cost of sample collection and storage. They can easily be shipped and analytically analyzed using HPLC. Furthermore, there is only minor discomfort associated with this minimally invasive method for the patients.Outpatients are asked to document their drug administrations (date, time, and dose amount) in a checklist and bring it to the study center at the visit for the blood sampling procedure.

Table 9 Summary of drug-specific data collection and blood sampling in the drug-drug-gene sub-study

Drugs for inclusion to sub-study	Blood sampling time windows						Endpoints
	t_0*	t_1 [0.5-1h]	t_2 [2-4h]	t_3 [5-8h]	t_4 [9-12h]	t_{ADR}**	
**Atorvastatin**	x	x	x	x	x	x	Lipid panels (TC, HDL-C, LDL-C, and TG)
**Capecitabine**	x	x	x	x		x	-5-FU related ADRs, e.g. hand-and-foot syndrome; leucopenia, neutropenia, thrombocytopenia. (Tumor response)
**Fluorouracil**	x	x	x	x		x	-5-FU related ADRs, e.g. hand-and-foot syndrome; leucopenia, neutropenia, thrombocytopenia. (Tumor response)
**Metoprolol**	x	x	x	x		x	-Resting blood pressure<br>-Heart rate
**Simvastatin**	x	x	x	x	x	x	Lipid panels (TC, HDL-C, LDL-C, and TG)
**Voriconazole**	x	x	x	x		x	-clinical symptoms and signs (e.g., body temperature, CT scans, MRI findings),<br>-microbiological response, (e.g. -microscopic examination, the cultivation result)<br>-serological tests (b-D-glucan test, galactomannan test)

* pre-dose.
**not later than 24h after event.

6.3.4. Extreme phenotypes

Extreme phenotypes are identified from patients who have consented to the main study. Extreme phenotypes are defined as those patients who:

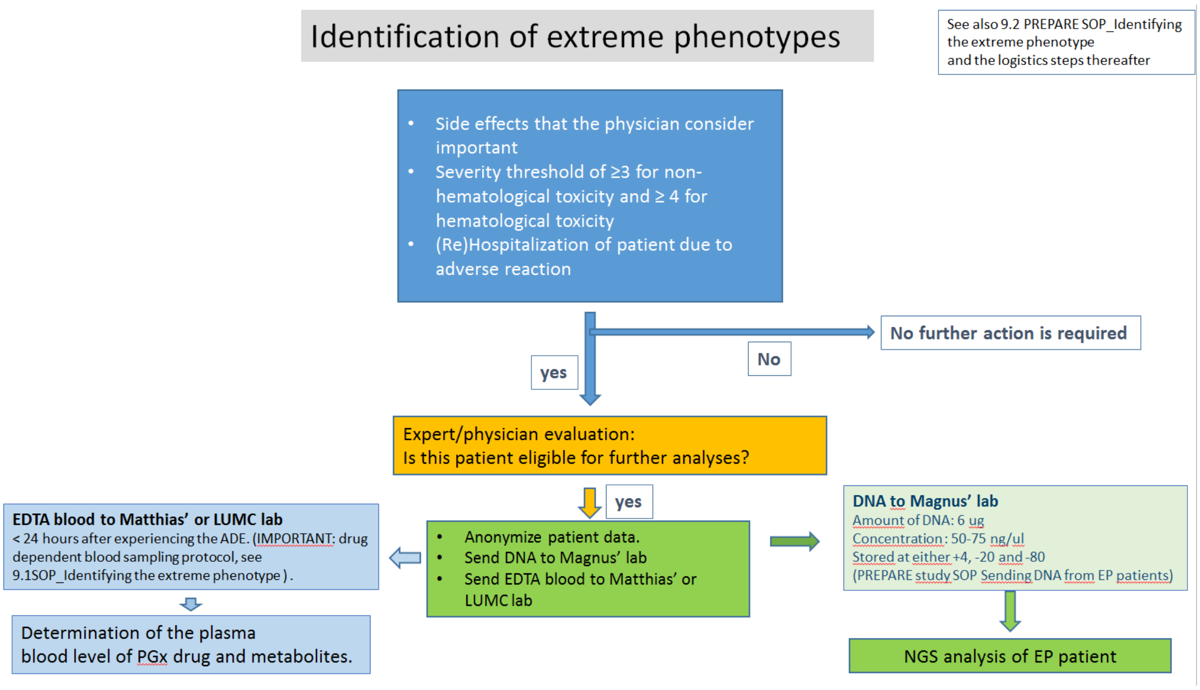

Figure 10: Flow-chart used to identify extreme phenotypes

Extreme phenotypes will be flagged and contacted by the research nurse to obtain an additional blood sample (10mL EDTA) within 24 hours of the ADR, if possible, to be able to determine the plasma blood level of the index drug and other concurrent drugs. The last dose and time of both index drug and co-medications must be recorded in the eCRF . This sample collection will be described in Standard Operating Procedure (9.1). A maximum of 35mL of blood (intended for research purposes) will be collected within a four week period.

To identify a possible genetic origin of the extreme phenotype, all patients included in the study will be asked to provide informed consent for additional NGS sequencing of the most relevant regions of 150-200 ADME genes (i.e. genes involved in the metabolism and distribution of drugs) with respect

to the drug treatment in question, encompassing 6-10 Mb of sequence, in an anonymous analysis. The chance of finding secondary findings is negligible within ADME genes. This data will only be used for exploratory analysis and not be implemented in clinical care or returned to the patient.
Once the identity of the subject has been removed, it will not be able to be reconnected to the subject. Therefore no secondary findings will be returned to the patients. NGS sequencing will be performed to search for novel variants associated with the extreme phenotype, only if the patient provided consent for anonymised NGS.

7. STATISTICAL ANALYSIS

All analyses are pre-specified in detail in the statistical analysis version 3.0

8. ASSESSMENT OF SAFETY

All drugs included within this study have previously been registered for use in humans. Therefore, no additional safety issues are expected in the study arm. However, to support and stimulate spontaneous reporting of ADEs, SAEs will be reported to national spontaneous reporting systems which are connected to the Eudravigilance Database.

8.1. Definition SAEs

Only adverse events related to the study procedures (i.e. blood draws, saliva collection, blood spot sampling, or PGx tests) will be regarded as SAEs.

A Serious Adverse Event (SAE) is an AE occurring during any study phase and at any dose of the investigational product, comparator or placebo that fulfils one or more of the following criteria:

- results in death
- is life-threatening
- requires inpatient hospitalisation or prolongation of existing hospitalisation
- results in persistent or significant disability/incapacity
- results in a congenital anomaly/birth defect
- is an important medical event that may jeopardise the patient or may require medical intervention to prevent one of the outcomes listed above.

Any adverse event leading to hospitalization or prolongation of hospitalization will be considered as ‘serious’, unless at least one of the following exceptions are met:

- the admission is pre-planned (e.g. elective or scheduled surgery arranged prior to the start of the study, documented in the patient’s file);
- prolonged hospitalization for technical, practical or social reasons, in the absence of an adverse event.

8.2

8.2. Methods for recording and follow-up of ADEs

All AEs will be followed up until they have abated, or until a stable situation has been reached, as done within the realm of routine clinical care. Recording and ADEs is the primary outcome of this study. ADEs will be monitored by both a trained research nurse and via patient reporting through an online spontaneous reporting system as further explained in Section 6.1.

8.3. Reporting of SAEs

All National Principal Investigators will have the responsibility for safety within their own country. The local Investigators will report SAEs to the National Principal Investigators who will report it to their national spontaneous reporting system.

8.4. Safety Committee

Since the study is not blinded, and health care providers may choose to implement PGx testing results in their prescriptions, no additional safety issues are expected. Therefore a Data Safety Monitoring Board would be superfluous and not provide any additional safety benefit.

8.5. Premature termination of the study

No additional safety issues are expected in the study arm, and therefore no criteria have been defined for premature termination of the study.

9. STRUCTURED RISK ANALYSIS

9.1. Potential issues of concern

Recruitment of sufficient numbers of patients

Successful recruitment of sufficient numbers of patients is crucial for the success of PREPARE. The consortium therefore includes multiple institutions with large number of patients (see Table 4), excellent logistics to effectively include patients, collect outcome data and extensive experience with clinical PGx research. In addition, similar to the situation in clinical trials, institutions will receive a fee per included patient as coverage of costs and as a motivation. Physicians and pharmacists will be offered an education program to improve knowledge and appreciation and to stimulate PGx implementation. Close collaboration with patient associations will be established and a patient representative is a member of the Advisory Board. The recruitment rate will be closely monitored during the project. If recruitment rate appears to slow additional centers will be included by the Principal Investigator of the respective country. To this end the Principal Investigators already contacted collaborating centers in their countries that have shown interest in including patients in the unlikely events that this is needed. Although we do not foresee that more centers will be needed this contingency plan removes the main potential threat for PREPARE.

To prevent overrepresentation of a single drug, the number of prescriptions for any drug included in the study will be capped at 10% of the total population.

Collection of clinical data

Poor collection of clinical data would be detrimental for the success of PREPARE. To remove this possible threat we will implement a central web-based database including eCRFs and extensive resources are allocated for dedicated research assistants for each implementation site.

Ethics

The risk that ethical aspects cannot be managed within the project is extremely low as we will be proactive and managing the ethical issues prospectively in a separate work package. As soon as a potential problem would appear, it will be managed by open discussion within the consortium and finding external advice. In addition, we are aware that a change in ethical and legal regulations in the EU and member states may be enforced soon and that possibly adaptations in the project are warranted. We have specifically addressed these changing ethical and legal regulations by Anne

Cambon-Thomsen, who is closely involved in EU ethical and legal regulations by representation in several European working parties. Additionally, a Scientific Advisory Board has been commissioned to advise on the development of the protocol to ensure a scientifically sound method (see Appendix 18.4).

Changes over time

A potential threat is that during the project the panel of drugs and genetic variants will change. For example, new drugs for which genetic testing is recommended may be marketed, drugs may be removed from the market, or a genetic test may become mandatory. In addition new genetic variants may be identified that are not in the current panel. At this moment it is difficult to judge if and how fast these developments will proceed. However, the PREPARE can cope with these developments since maintenance and development of guidelines are responsibility of the DPWG. The discovery of novel genetic variants can easily be addressed by incorporating them in the genotyping platform which is open and flexible by design.

In addition, institutions which were randomized to start with PGx-guided prescribing in the first time block may be reluctant to start the standard of care control period (see Figure 2) since they have had experience with PGx-guided prescribing and may be more aware of pharmacological issues. To ameliorate this risk, and also for scientific reasons (i.e. minimize risk of physician carry over effect), patients will be blinded for the study outcome until the end of the inclusion period.

Clinicians' acceptance of clinical decision support tools

A potential risk is that CDS tools will not be (properly) used by clinicians at the participating sites. To address this issue, clinicians will receive extensive training and education. In addition acceptance of CDS will be closely monitored throughout the entire project and CDS tools will be improved through an iterative process based on clinician and patient feedback.

Differences in health care settings

Obviously, the differences in health care settings across the EU are a challenge for the project. Indeed, differences in languages, level of use of IT in health care, standards of care, regulatory issues etc. will differ considerably. However, in this H2020 project, implementation of personalised medicine taking into account the diversity in health care settings is an explicit aim. We have chosen the participating countries and settings in a way that the diversity is covered maximally. In this way, models for effective PGx implementation emerging form the PREPARE project can be extrapolated across Europe. However, the design of the project is chosen in a way that assessment of clinical

outcome and cost-effectiveness is not hampered by the differences in health care settings. To this end, a prospective cross-over design was chosen and every institution serves as its own control.

Management of the project

No threats are foreseen with work that deals with management issues. The study coordinator has ample experience with managing large international projects. A detailed and clear organizational structure has been set up and clear decision-making procedures have been described (see Appendix 18.5).

10.DATA HANDLING AND RECORD KEEPING

10.1. Electronic Case Report Forms

An electronic case report form (eCRF) will be completed for each included subject. The subject's identity will always remain confidential. The database will only contain the patient study ID code and never the name of the patient. The completed eCRFs are property of the PREPARE study and should not be made available to third parties (except for authorised representatives of appropriate regulatory authorities) without written permission from the PREPARE study. All data in the eCRFs will be in English. If necessary, the monitor should translate any information or comments recorded in another local language. The National Principal Investigator is responsible for ensuring the accuracy, completeness and timeliness of the data recorded in the electronic CRFs.

A TRE (Trial Related Events) section will be incorporated within the eCRF. This will record the occurrence of causal (definite, probable or possible), clinically relevant (Grade 2, 3,4, or 5) ADEs which are associated with a drug-genotype. This filtered data will be used for the primary analysis.

The eCRF database software ProMISe (Project Manager Internet Server) (see Section 10.5) has the following specifications:

- Central and secure storage of the study data in a central study database. The database server is situated at Leiden University Medical Center
- Participating centres have access to the eCRF through a log-in
- Access levels and restrictions to the central database, to the participating centres and to the subject data is configured and managed centrally.
- Allows quality control and data quality checks
- Allows central monitoring as well as local monitoring and source data verification.
- Allows inclusion of all sites to me monitored. Through this tool, the 10% capping if index drugs will be monitored by LUMC
- All changes to the data in the database, starting from the moment of creation of the data item, will be stored in an audit trail and will be time, date and user stamped by the system without any user intervention.
- The data in the database will be available for easy but quality controlled export to other applications to allow statistical analysis.

10.2. Data security and subject privacy

Data on subjects collected in eCRFs will be documented in an pseudonymous fashion such that a patient will not be identifiable from the information recorded on the eCRF. The confidentiality of those documents which could identify the patients must respect the subjects' privacy and the norms of confidentiality in accordance with the applicable national regulatory requirements. The National Principal Investigator will maintain a subject identification code list.

10.3. Access to source data and record retention

The National Principal Investigator is responsible for archiving all source documents and the study documentation as required by national laws and regulations. The Investigator will retain all study records for 15 years if not instructed otherwise by the sponsor.

10.4. Storage of human material

- Blood and saliva samples used to collect DNA will be stored until they are used up.
- The blood sample at the time of an "extreme phenotype" ADR will be kept until they are used up.
- The blood spot samples used to elucidate a drug-drug-gene interaction (sub-study) will be kept until used up.
- Other samples of human material which are collected within the realm of routine care will be kept in accordance to national laws and regulations.

10.5. Data management

ProMISe (https://www.msbi.nl/promise/) is a web-based relational database management system for the design and maintenance of clinical data management. ProMISe is developed within the Leiden University Medical Center (LUMC) Section of Advanced Data Management (ADM) who work closely with the department of Biostatistics and Bioinformatics. The ADM is NEN7510 certified and ProMISe meets the requirements for data-safety and privacy as set by the international legislations. ProMISe facilitates storage, exchange and retrieval of data according to the security conditions demanded by the GCP.

11.QUALITY CONTROL AND QUALITY ASSURANCE

11.1. Monitoring

A professional clinical research organization (CRO) will be subcontracted to perform the monitoring of PREPARE. The monitors are required to have a health related background training and specific GCP training. Trial monitors will visit all sites during the study. Trial monitors will oversee the progress of the study, ensure the wellbeing of study subjects, ensure accuracy, completeness of study data and monitor compliance with the study protocol, SOPs, ICH-GCP and the Declaration of Helsinki, EU directives and applicable regulatory requirements. A monitoring plan will be written before study initiation.

The monitor will visit each site three times during the study (two monitoring visits and once close-out visit). The monitor will check that each subject has provided written informed before participating in the study. The monitor will follow the patient inclusion and patient withdrawal frequencies, confirm the completeness of the trial master file and the investigator file, verify 100% of all informed consent procedures and paperwork, check 10% of eCRFs and all source files. Any discrepancies of data will be documented and explained in the monitoring reports. If necessary, the data in the database can be changed in order to match with the source data, by a person with sufficient access rights to the database to make changes of this type. Additionally, the monitor will control compliancy to the study protocol.

11.2. Direct access to documents

By signing the study protocol the participating centres confirm that they will provide source data during monitoring and inspection by regulatory agencies or local ethics committees. The investigational sites will give permission for trial-related monitoring, audits and provide direct access to source data and hospital records required.

12.TEMPORARY HALT AND PREMATURE TERMINATION OF THE STUDY

The investigator will notify the accredited ethical committee of the end of the study within a period of 8 weeks. The end of the study is defined as the last patient's last visit/telephone contact.
The sponsor will notify the ethical committee immediately of a temporary halt of the study, including the reason of such an action. In case the study is ended prematurely, the sponsor will notify the accredited ethical committee within 15 days, including the reasons for the premature termination. Within one year after the end of the study, the investigator/sponsor will submit a final study report with the results of the study, including any publications/abstracts of the study, to the accredited ethical committee.

12.1. Temporary halt of study

The sponsor will suspend the study if there is sufficient ground that continuation of the study will jeopardise subject health or safety. The sponsor will notify the accredited Ethical Committee without undue delay of a temporary halt including the reason for such an action. The study will be suspended pending a further positive decision by the accredited Ethical Committee. The investigator will be responsible for keeping all patients informed. The sponsor will notify each country's ethics committee immediately of a temporary halt of the study, including the reason of such an action.

12.2. Premature termination of study

The U-PGx Consortium reserves the right to discontinue the study prior to inclusion of the intended number of subjects, but intends to exercise this right only for valid scientific or administrative reasons, and only after consultation with the National Principal Investigators. After such a decision, all study-related materials must be collected without delay and all eCRFs must be completed as far as possible.

13.ETHICS

13.1. Ethical conduct of the study

The PREPARE study will be conducted according to the study protocol, applicable regulatory requirements, ICH/GCP, the ethical principles of the Declaration of Helsinki (latest version 2013, www.wma.net) and in accordance to national laws and legislation.

13.2. Independent ethics committee

It is the responsibility of the National Principal Investigators to obtain ethical approval or favourable opinion in writing of the study protocol and protocol amendments, the patient information leaflet and the informed consent form from the before enrolment of any subject into the study.

1.1 Patient information and informed consent

Before any site can begin recruitment, they must receive approval of the study by the regulatory authority (if required) and accredited ethical committee, as well as other country-specific required approvals. Recruiters (treating physicians of pharmacists) will be provided with a checklist of inclusion and exclusion criteria and a recruitment study log book. To participate in the study, eligible patients must give written, informed consent

13.2.1. Recruitment in community pharmacies

Patients receiving a first prescription for at least one drug for which a DPWG guideline is available (Table 5) will be recognized by the treating pharmacist by a newly developed automated trigger. This trigger will invite the pharmacist to check eligibility criteria for this particular patient in the PREPARE study in real time.

13.2.2. Informed Consent and Patient Brochure

Informed consent will be obtained by recruiting pharmacists and research nurses who are informed of patient eligibility by the recruiting pharmacist and trained to answer questions about the protocol and consent forms.

The consent process will begin with a verbal description of the study as outlined by the consent form. This process includes a verbal description of what the patient would be asked to do if he or she participated in the study, the risks and benefits of participating in the study, confidentiality of health information while in the study, and his or her right to refuse or withdraw participation without consequence and reason.

Informed consent forms signed in twofold by the patient and the Investigator or delegate shall be filed in the Investigator's File for possible future audits and inspections. A copy of the patient information leaflet and an originally signed informed consent form will be given to the subject.

13.3. Changes to the study protocol

Amendments are changes made to the research after a favourable opinion by the accredited Ethical Committee has been given. All amendments will be notified to the ethical committee that gave a favourable opinion. All substantial amendments will be notified to the ethical committee. Non-substantial amendments will not be notified to the accredited ethical committee, but will be recorded and filed by the sponsor.

13.4. Annual progress report

The National Principal Investigator will submit a summary of the progress of the study to the accredited ethical committee once a year. Information will be provided on the date of inclusion of the first subject, numbers of subjects included and numbers of subjects that have completed the study, SAEs, other problems, and amendments.

14.INSURANCE

The sponsor/investigator has received exemption for patient insurance for this protocol.

15.STUDY REPORT

The study report is sent to each national regulatory agency (if applicable), independent ethics Committees and to the European Commission.

16.PUBLIC DISCLOSURE AND PUBLICATION POLICY

The clinical study report will form of manuscripts intended for publication in a medical/scientific journal. Publication policy details are presented in the U-PGx Consortium Agreement.

17.REFERENCES

1. Ehmann F. *et al.* Pharmacogenomic information in drug labels: European Medicines Agency perspective. Pharmacogenomics J. 2015 Feb 24;15(3):201-10
2. Swen JJ. *et al.* Pharmacogenetics: from bench to byte--an update of guidelines. Clin Pharmacol Ther. 2011 May;89(5):662-73.
3. Swen JJ. *et al.* Translating pharmacogenomics: challenges on the road to the clinic. PLoS Med. 2007 Aug;4(8):e209.
4. Dunnenberger HM. e*t al.* Preemptive clinical pharmacogenetics implementation: current programs in five US medical centers. Annu Rev Pharmacol Toxicol. 2015;55:89-106.
5. https://ctep.cancer.gov/protocoldevelopment/electronic_applications/ctc.htm
6. Gallagher RM, et al. Development and inter-rater reliability of the Liverpool adverse drug reaction causality assessment tool. PLoS One 2011;6(12):e28096.
7. van Balveren-Slingerland L. *et al.* Web-based intensive monitoring of adverse events following influenza vaccination in general practice. Vaccine. 2015 [Epub ahead of print]
8. Härmark L, *et al.* Intensive monitoring of pregabalin: results from an observational, Web-based, prospective cohort study in the Netherlands using patients as a source of information. Drug Saf. 2011 1;34(3):221-31.

18.APPENDICIES

18.1. National Coordinators and Principal Investigators

Appendix Table 1 National Coordinators and Principal Investigators

Country	Name Institute	National Principal Investigator	Email address
**The Netherlands**	Leiden University Medical Center	Jesse J. Swen	j.j.swen@lumc.nl
**United Kingdom**	University of Liverpool	Munir Pirmohamed	munirp@liverpool.ac.uk
**Austria**	Medical University of Vienna	Gere Sunder-Plassmann	gere.sunder-plassmann@meduniwien.ac.at
**Italy**	Centro di Riferimento Oncologico	Giuseppe Toffoli	gtoffoli@cro.it
**Spain**	Servicio Andaluz de Salud	Cristina Davila Fajardo	cristinal.davila.sspa@juntadeandalucia.es
**Greece**	University of Patras	George P. Patrinos	gpatrinos@upatras.gr
**Slovenia**	University of Ljubljana	Vita Dolzan	vita.dolzan@mf.uni-lj.si

18.2. Flow Charts of Patient Journey

18.2.1. General Flow Chart of Patient Journey

Study arm - logistics for index drug

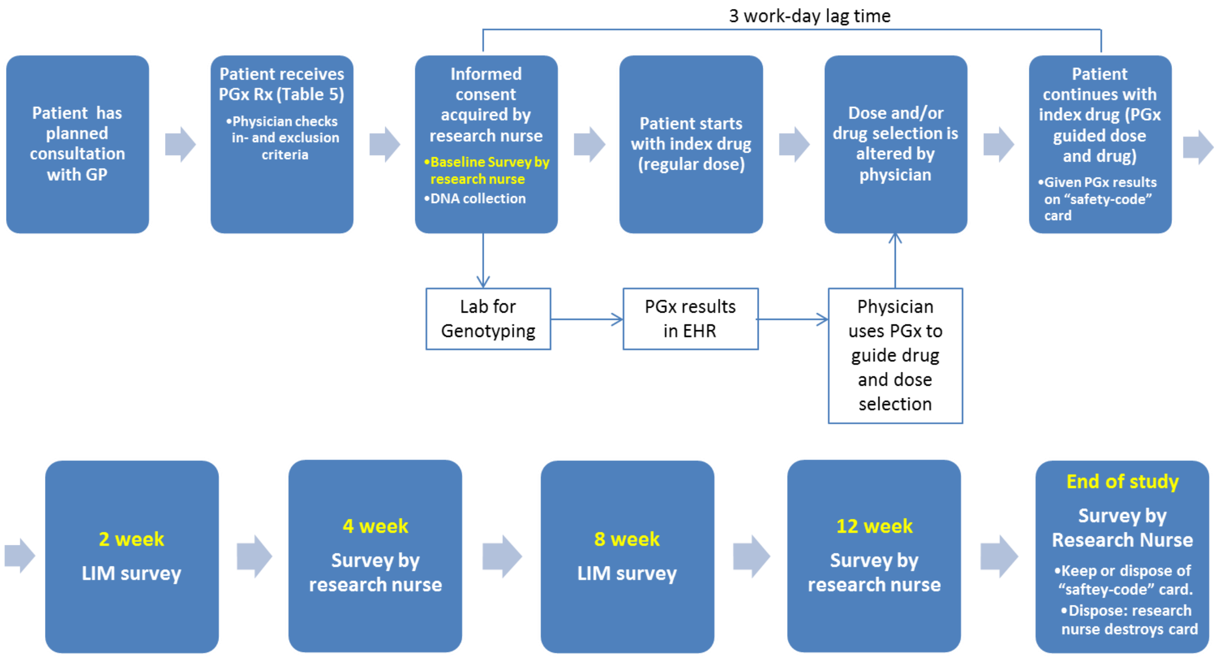

Standard of care arm - logistics for index drug

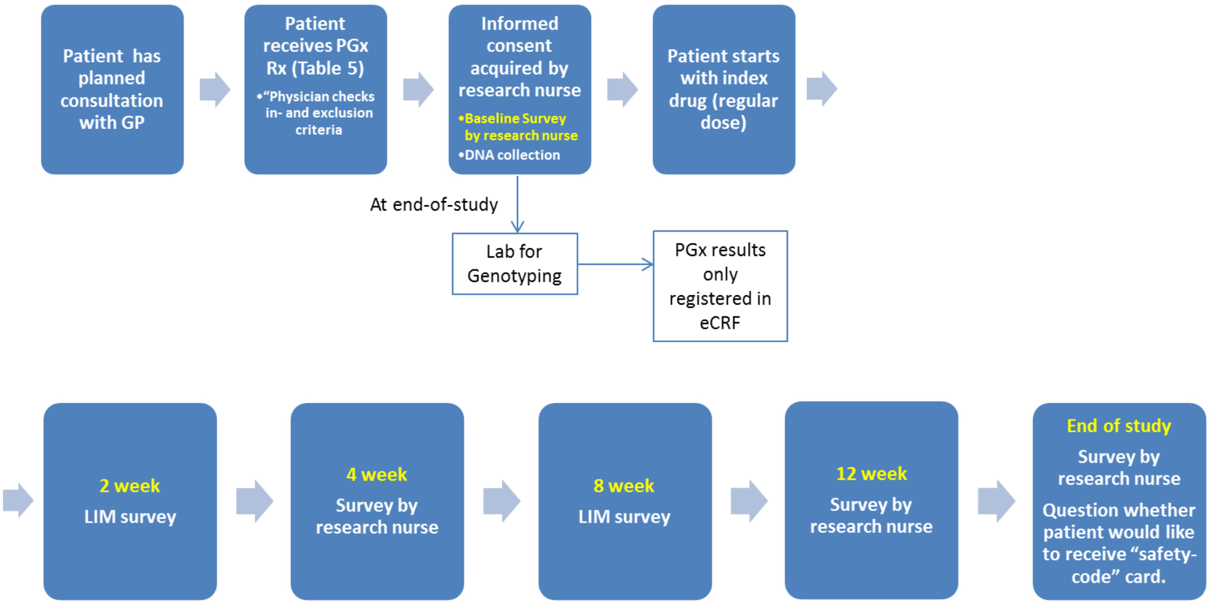

18.2.2. Site Specific Flow Chart of Patient Journey

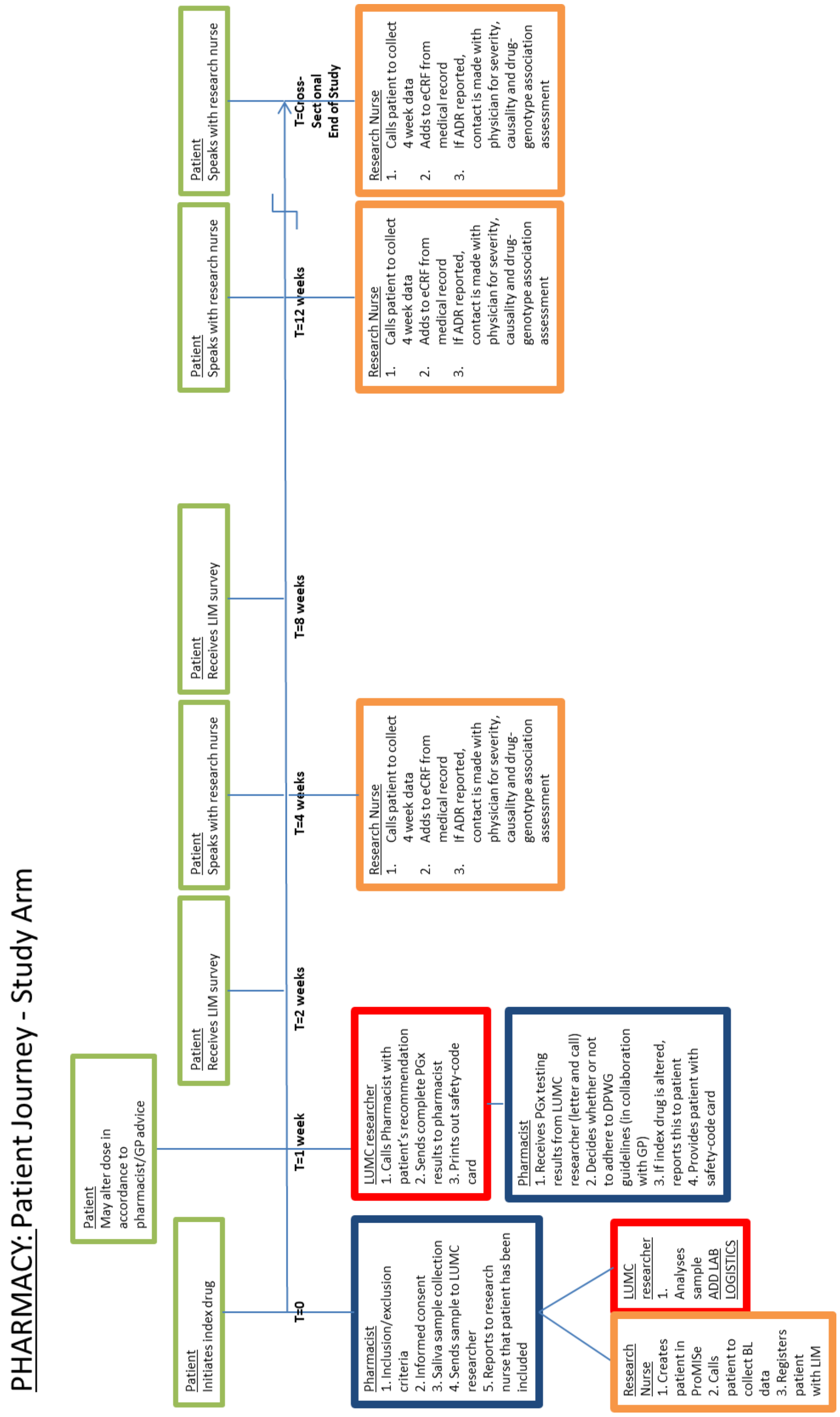

PHARMACY: Patient Journey – Standard of Care Arm

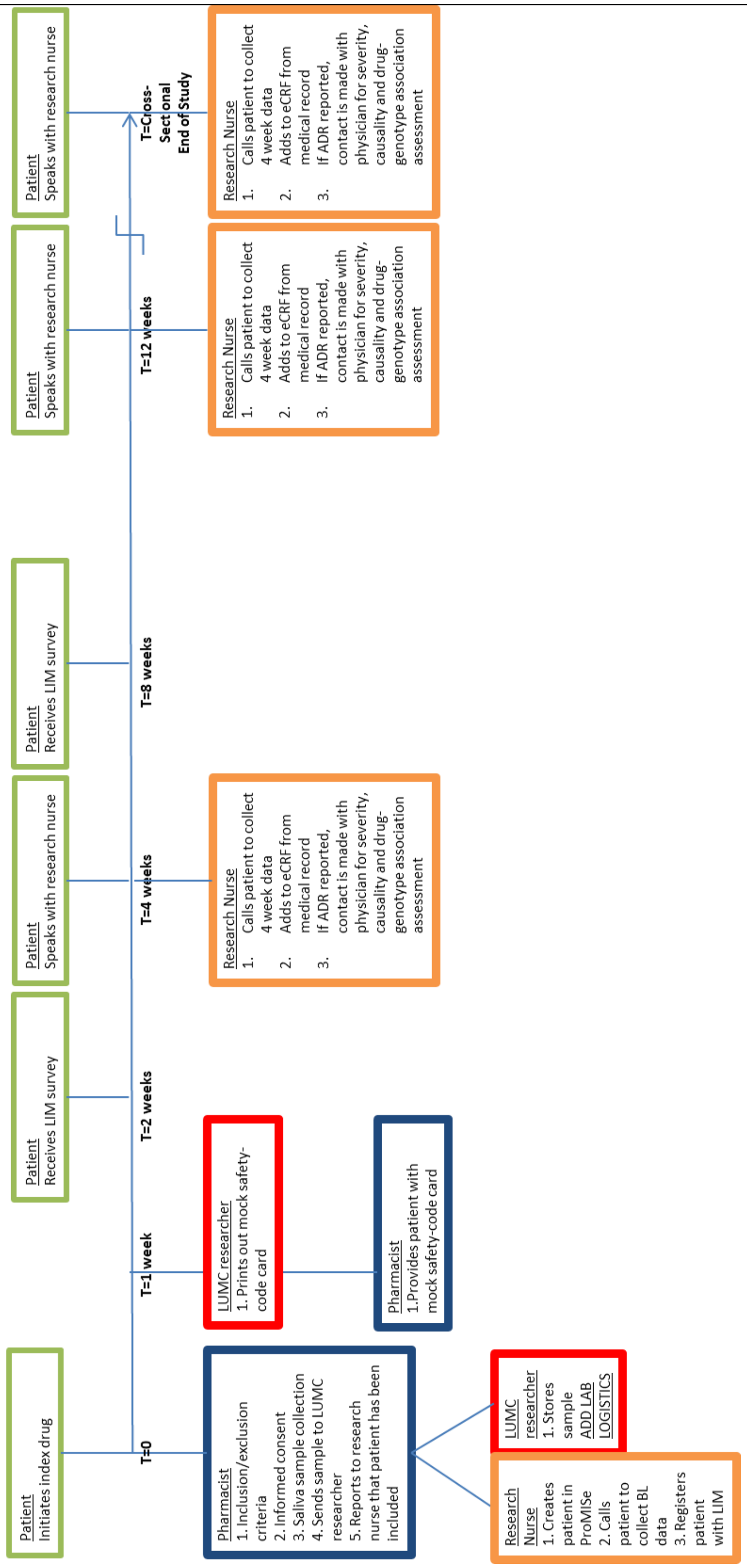

18.3. DPWG Guidelines

The DPWG recommendations below have initially translated from Dutch to English by a certified translation company. The validated translations into all local languages will be completed in December 2016.

Drug-gene interaction	Recommendation
**1480**<br>**CYP2D6 PM:**<br>**CLOMIPRAMINE**	The genetic polymorphism leads to a reduction in CYP2D6's metabolic capacity. This can cause the plasma concentrations of clomipramine and the active metabolite to increase, while reducing those of the potentially cardiotoxic hydroxy metabolites.<br><br>Recommendation:<br>* Indication DEPRESSION:<br>- Reduce the dose to 50% of the normal dose, and monitor the plasma concentrations of clomipramine and N-desmethylclomipramine<br>* Indication ANXIETY DISORDERS:<br>- Select an alternative<br>Some examples of antidepressants that are only partially metabolized by CYP2D6, or not at all, are citalopram and sertraline. If no alternative is possible and adverse effects occur:<br>- Reduce the dose by 50%, and monitor the plasma concentrations of clomipramine and N-desmethylclomipramine. It is not known whether it is possible to reduce the dose to a point at which the adverse effects disappear while efficacy is maintained. Clomipramine and N-desmethylclomipramine both contribute to these adverse effects. Efficacy, however, is due solely to clomipramine.
**1481**<br>**CYP2D6 IM:**<br>**CLOMIPRAMINE**	The genetic polymorphism leads to a reduction in CYP2D6's metabolic capacity. This can cause the plasma concentrations of clomipramine and the active metabolite to increase, while reducing those of the potentially cardiotoxic hydroxy metabolites.<br><br>Recommendation:<br>- Reduce the dose to 70% of the normal dose, and monitor the plasma concentrations of clomipramine and desmethylclomipramine. For the indication of obsessive compulsive disorder and other anxiety disorders only clomipramine is relevant. For toxicity and the indication of depression, both are relevant.
**1482**<br>**CYP2D6 UM:**<br>**CLOMIPRAMINE**	The genetic polymorphism leads to an increase in CYP2D6's metabolic capacity. This can cause the plasma concentrations of clomipramine and the active metabolite to decrease, while increasing those of the potentially cardiotoxic hydroxy metabolites.<br><br>Recommendation:<br>- Select an alternative<br>Some examples of antidepressants that are only partially metabolized by CYP2D6, or not at all, are citalopram and sertraline. If no alternative is possible:<br>- Increase the dose to 150% of the normal dose, and monitor the plasma concentrations of clomipramine and desmethylclomipramine.<br>For the indication of obsessive compulsive disorder and other anxiety disorders only clomipramine is relevant. For toxicity and the indication of depression, both are relevant.
**1538**<br>**CYP2D6 PM:**<br>**VENLAFAXINE**	The genetic polymorphism leads to a reduction in CYP2D6's metabolic capacity. This can cause the plasma concentration of venlafaxine to increase, while reducing that of the active metabolite O-desmethylvenlafaxine. There are indications that the efficacy of venlafaxine is decreased in depressive patients with this genetic polymorphism.<br><br>Recommendation:<br>Based on the literature, it is not possible to make a sufficiently well substantiated recommendation with regard to dose adjustment.<br>- Select an alternative<br>Some examples of antidepressants that are only partially metabolized by CYP2D6, or not at all, are citalopram and sertraline.<br>If no alternative is possible and adverse effects occur:<br>- Reduce the dose, check the plasma concentrations of venlafaxine and O-desmethylvenlafaxine. It is not known whether it is possible to reduce the dose to a point at which the adverse effects disappear while efficacy is maintained. In general, it is assumed that efficacy is determined by the sum of the plasma concentrations of venlafaxine and O-desmethylvenlafaxine. The adverse effects, however, do not seem to be related to this sum.<br>In addition, observations have shown that the efficacy of venlafaxine is decreased in depressive patients with this genetic polymorphism.
**1539**<br>**CYP2D6 IM:**<br>**VENLAFAXINE**	The genetic polymorphism leads to a reduction in CYP2D6's metabolic capacity. This can cause the plasma concentration of venlafaxine to increase, while reducing that of the active metabolite O-desmethylvenlafaxine.

	Recommendation:<br>Based on the literature, it is not possible to make a sufficiently well substantiated recommendation with regard to dose adjustment.<br>- Select an alternative<br>Some examples of antidepressants that are only partially metabolized by CYP2D6, or not at all, are citalopram and sertraline. If no alternative is possible and adverse effects occur: - reduce the dose, check the plasma concentrations of venlafaxine and O-desmethylvenlafaxine.<br>It is not known whether it is possible to reduce the dose to a point at which the adverse effects disappear while efficacy is maintained. In general, it is assumed that efficacy is determined by the sum of the plasma concentrations of venlafaxine and O-desmethylvenlafaxine. The adverse effects, however, do not seem to be related to this sum.
**1540**<br>**CYP2D6 UM: VENLAFAXINE**	The genetic polymorphism leads to an increase in CYP2D6's metabolic capacity. This can cause the plasma concentration of venlafaxine to decrease, while increasing that of the active metabolite O-desmethylvenlafaxine.<br><br>Recommendation:<br>- Be alert to a possible reduction in the sum of the plasma concentrations of venlafaxine and its active metabolite O-desmethylvenlafaxine. If necessary, increase the dose to 150% of the normal dose. If dose adjustment based on therapeutic drug monitoring is not possible, an alternative should be selected. Some examples of antidepressants that are only partially metabolized by CYP2D6, or not at all, are citalopram and sertraline.
**1542**<br>**CYP2D6 PM: ARIPIPRAZOL**	The genetic polymorphism leads to a decrease in CYP2D6's metabolic capacity, which can result in an increase in the sum of the plasma concentrations of aripiprazole and the active metabolite.<br><br>Recommendation:<br>- Advise the prescriber to prescribe no more than 10 mg/day or 300 mg/month (67%-75% of the normal maximum dose of aripiprazole).
**1544**<br>**CYP2D6 PM: IMIPRAMINE**	The genetic polymorphism leads to a decrease in CYP2D6's metabolic capacity, which can result in an increase in the plasma concentrations of imipramine and the active metabolite.<br><br>Recommendation:<br>- Reduce the dose to 30% of the normal dose, and monitor the plasma concentrations of imipramine and desipramine for the purpose of setting the maintenance dose.
**1545**<br>**CYP2D6 IM: IMIPRAMINE**	The genetic polymorphism leads to a reduction in CYP2D6's metabolic capacity, which can result in an increase in the plasma concentrations of imipramine and desipramine.<br><br>Recommendation:<br>- Reduce the dose to 70% of the normal dose, and monitor the plasma concentrations of imipramine and desipramine for the purpose of setting the maintenance dose.
**1546**<br>**CYP2D6 UM: IMIPRAMINE**	The genetic polymorphism leads to an increase in CYP2D6's metabolic capacity, which can result in a decrease in the plasma concentrations of imipramine and desipramine, and an increase in that of the hydroxy metabolites.<br><br>Recommendation:<br>- Select an alternative<br>Some examples of antidepressants that are only partially metabolized by CYP2D6, or not at all, are citalopram and sertraline.<br>If no alternative is possible:<br>- Increase the dose to 170% of the normal dose, and monitor the plasma concentrations of imipramine and desipramine for the purpose of setting the maintenance dose.
**1547**<br>**CYP2D6 PM: ZUCLOPENTHIXOL**	The genetic polymorphism leads to a reduction in CYP2D6's metabolic capacity, which can result in an increase in the plasma concentration of zuclopenthixol.<br><br>Recommendation:<br>- Advise the prescriber to reduce the dose to 50% of the normal dose or to prescribe an alternative, in accordance with existing guidelines.<br>Some examples of antipsychotics that are only partially metabolized by CYP2D6, or not at all, are flupentixol, quetiapine, olanzapine and clozapine.
**1548**<br>**CYP2D6 IM: ZUCLOPENTHIXOL**	The genetic polymorphism leads to a reduction in CYP2D6's metabolic capacity, which can result in an increase in the plasma concentration of zuclopenthixol.<br><br>Recommendation:<br>- Advise the prescriber to reduce the dose to 75% of the normal dose or to prescribe an alternative, in accordance with existing guidelines.<br>Some examples of antipsychotics that are only partially metabolized by CYP2D6, or not at all, are flupentixol, quetiapine, olanzapine and clozapine.

**1549**<br>**CYP2D6 UM: ZUCLOPENTHIXOL**	The genetic polymorphism leads to an increase in CYP2D6's metabolic capacity, which can result in a decrease in the plasma concentration of zuclopenthixol.<br><br>Recommendation:<br>No data from studies into the pharmacokinetics and effects of zuclopenthixol in this phenotype have been published.<br>- Advise the prescriber, as a precaution, to be alert to a decrease in the plasma concentration of zuclopenthixol and, if necessary, to increase the dose based on the clinical effect or to prescribe an alternative, in accordance with existing guidelines.<br>Some examples of antipsychotics that are only partially metabolized by CYP2D6, or not at all, are flupentixol, quetiapine, olanzapine and clozapine.
**1552**<br>**CYP2D6 PM: HALOPERIDOL**	The genetic polymorphism leads to a decrease in CYP2D6's metabolic capacity, which can result in an increase in the plasma concentrations of haloperidol and the active metabolite.<br><br>Recommendation:<br>Advise the prescriber to:<br>- Either reduce the starting dose to 50% of the normal starting dose, and to adjust the dose based on the effect<br>- Or prescribe an alternative.<br>Some examples of antipsychotics that are only very partially metabolized by CYP2D6, or not at all, are flupentixol, flufenazine, quetiapine, olanzapine and clozapine.
**1553**<br>**CYP2D6 UM: HALOPERIDOL**	The genetic polymorphism leads to an increase in CYP2D6's metabolic capacity, which can result in a decrease in the plasma concentrations of haloperidol and reduced haloperidol.<br><br>Recommendation:<br>Based on the limited amount of literature available, it is not possible to make a sufficiently well substantiated recommendation with regard to dose adjustment.<br>Advise the prescriber to:<br>- Either be alert to a possible decrease in the plasma concentrations of haloperidol and reduced haloperidol and to increase the dose based on therapeutic drug monitoring<br>- Or to prescribe an alternative, in accordance with existing guidelines.<br>Some examples of antipsychotics that are only very partially metabolized by CYP2D6, or not at all, are flupentixol, flufenazine, quetiapine, olanzapine and clozapine.
**1554**<br>**CYP2D6 IM: METOPROLOL**	The genetic polymorphism leads to a reduction in CYP2D6's metabolic capacity, which can result in an increase in the plasma concentration of metoprolol.<br><br>Recommendation:<br>* HEART FAILURE:<br>- Advise the prescriber, if possible, to prescribe an alternative, in accordance with existing guidelines or, when setting the dose, to make allowance for the fact that, as a result of the gene polymorphism, it may be necessary to reduce the required dose to 50% of the normal dose.<br>Possible alternatives are bisoprolol or carvedilol.<br>Bisoprolol: advantage: it is not metabolized by CYP2D6; disadvantage: elimination is dependent on renal function.<br>Carvedilol: advantage: elimination is not dependent on renal function; disadvantage: is metabolized by CYP2D6 (albeit to a lesser extent than metoprolol). If no alternative is selected: advise patients that, should their heart failure symptoms worsen, or if they should experience adverse effects such as bradycardia and cold extremities, they should contact the prescriber.<br><br>* OTHER INDICATIONS:<br>No dose adjustment is necessary, as there are no indications in the literature that an increase in the area under the curve (AUC) or in the plasma concentration has a major effect in terms of blood pressure change.<br>- Draw the prescriber's attention to the potential increase in plasma concentration, and to the possibility of an increased risk of adverse effects, such as bradycardia. If necessary, as a precaution, an alternative can be selected. Some examples of β-blockers that are only partially metabolized by CYP2D6, or not at all, are atenolol and bisoprolol. If no alternative is selected: advise patients that, if they should experience adverse effects such as bradycardia and cold extremities, they should contact the prescriber.
**1555**<br>**CYP2D6 PM: METOPROLOL**	The genetic polymorphism leads to a reduction in CYP2D6's metabolic capacity, which can result in an increase in the plasma concentration of metoprolol.<br><br>Recommendation:<br>* HEART FAILURE:<br>- Advise the prescriber to prescribe an alternative, in accordance with existing guidelines or, when setting the dose, to make allowance for the fact that, as a result of the gene polymorphism, it may be necessary to reduce the required dose to 25% of the normal dose.<br>Possible alternatives are bisoprolol or carvedilol.<br>Bisoprolol: advantage: it is not metabolized by CYP2D6; disadvantage: elimination is dependent on renal

	function.<br>Carvedilol: advantage: elimination is not dependent on renal function; disadvantage: is metabolized by CYP2D6 (albeit to a lesser extent than metoprolol). If no alternative is selected: advise patients that, should their heart failure symptoms worsen, or if they should experience adverse effects such as bradycardia and cold extremities, they should contact the prescriber.<br><br>* OTHER INDICATIONS:<br>No dose adjustment is necessary, as there are no indications in the literature that an increase in the area under the curve (AUC) or in the plasma concentration has a major effect in terms of blood pressure change.<br>- Draw the prescriber's attention to the potential increase in plasma concentration, and to the possibility of an increased risk of adverse effects, such as bradycardia. If necessary, as a precaution, an alternative can be selected. Some examples of β-blockers that are only partially metabolized by CYP2D6, or not at all, are atenolol and bisoprolol. If no alternative is selected: advise patients that, if they should experience adverse effects such as bradycardia and cold extremities, they should contact the prescriber.
**1556**<br>**CYP2D6 UM: METOPROLOL**	The genetic polymorphism leads to an increase in CYP2D6's metabolic capacity, which can result in a decrease in the plasma concentration of metoprolol.<br><br>Recommendation:<br>- Advise the prescriber to prescribe an alternative, in accordance with existing guidelines.<br>Possible alternatives are:<br>- HEART FAILURE: bisoprolol or carvedilol.<br>Bisoprolol: advantage: it is not metabolized by CYP2D6; disadvantage: elimination is dependent on renal function.<br>Carvedilol: advantage: elimination is not dependent on renal function; disadvantage: is metabolized by CYP2D6 (albeit to a lesser extent than metoprolol).<br>- OTHER INDICATIONS: atenolol or bisoprolol. Neither are metabolized by CYP2D6.<br>If no alternative is possible: advise the prescriber to increase the dose, based on efficacy and adverse effects, to 250% of the normal dose.
**1557**<br>**CYP2D6 IM: NORTRIPTYLINE**	The genetic polymorphism leads to a reduction in CYP2D6's metabolic capacity, which can result in an increase in the plasma concentration of nortriptyline.<br><br>Recommendation:<br>- Reduce the dose to 60% of the normal dose, and monitor the plasma concentrations of nortriptyline for the purpose of setting the maintenance dose.
**1558**<br>**CYP2D6 PM: NORTRIPTYLINE**	The genetic polymorphism leads to a reduction in CYP2D6's metabolic capacity, which can result in an increase in the plasma concentration of nortriptyline.<br><br>Recommendation:<br>- Reduce the dose to 40% of the normal dose, and monitor the plasma concentration of nortriptyline for the purpose of setting the maintenance dose.
**1559**<br>**CYP2D6 UM: NORTRIPTYLINE**	The genetic polymorphism leads to an increase in CYP2D6's metabolic capacity, which can result in a decrease in the plasma concentration of nortriptyline, and an increase in that of the active metabolite E-10-OH-nortriptyline.<br><br>Recommendation:<br>- Select an alternative<br>Some examples of antidepressants that are only partially metabolized by CYP2D6, or not at all, are citalopram and sertraline.<br>If no alternative is possible:<br>- Increase the dose to 160% of the normal dose, monitor the plasma concentration of nortriptyline, and be alert to an anticipated increase in the plasma concentration of the potentially cardiotoxic, active metabolite E-10-hydroxynortriptyline.
**1565**<br>**CYP2D6 UM: PAROXETINE**	Genetic variation results in increased conversion of paroxetine by the enzyme CYP2D6. This can reduce efficacy.<br><br>Recommendation:<br>Based on the literature, it is not possible to make a recommendation with regard to dose adjustment.<br>If possible, select an alternative.<br>Some examples of antidepressants that are only partially metabolized by CYP2D6, or not at all, are citalopram or sertraline.
**1566**<br>**FACT. V LEIDEN HOMOZYG: CONTRACEP. WITH OESTR.**	Homozygosity for the Factor V Leiden genetic polymorphism causes an increased tendency for blood to clot, resulting in an increased risk of venous thromboembolism. Oestrogen-containing contraceptives may increase this risk still further.<br><br>Recommendation:<br>* In the event of NUMEROUS CASE HISTORIES in a family OR a previous case of VENOUS THROMBOSIS:<br>- Advise the prescriber to avoid the use of oestrogen-containing contraceptives and, as an alternative, to

	prescribe a non-hormone-containing contraceptive, such as a copper-bearing intrauterine device (IUD). Another option is a contraceptive method that only uses progestogens, such as the birth control injection, an IUD with levonorgestrel or etonogestrel implant.<br><br>* OTHER CASES:<br>- Advise patients to avoid additional risk factors for thrombosis (obesity, smoking, etc.).
**1567**<br>**FACT. V LEIDEN HETEROZYG: CONTRACEP. WITH OESTR.**	Heterozygosity for the Factor V Leiden genetic polymorphism causes an increased tendency for blood to clot, resulting in an increased risk of venous thromboembolism. Oestrogen-containing contraceptives may increase this risk still further.<br><br>Recommendation:<br>* In the event of NUMEROUS CASE HISTORIES in a family OR a previous case of VENOUS THROMBOSIS:<br>- Advise the prescriber to avoid the use of oestrogen-containing contraceptives and, as an alternative, to prescribe a non-hormone-containing contraceptive, such as a copper-bearing intrauterine device (IUD). Another option is a contraceptive method that only uses progestogens, such as the birth control injection, an IUD with levonorgestrel or etonogestrel implant.<br><br>* OTHER CASES:<br>- Advise patients to avoid additional risk factors for thrombosis (obesity, smoking, etc.).
**1583**<br>**CYP2D6 PM: CODEINE**	The genetic polymorphism reduces the conversion of codeine into morphine. This can lead to a reduction in pain relief.<br><br>Recommendation:<br>* In the event of COUGHING:<br>- No action required<br><br>* In the event of PAIN:<br>Based on the limited amount of literature, it is not possible to make a sufficiently well substantiated recommendation with regard to dose adjustment for this phenotype.<br>- Select an alternative<br>In this connection, do not select tramadol as this is also metabolized by CYP2D6.<br>Oxycodone is also metabolized by CYP2D6, but can usually be adjusted to give adequate pain relief without giving rise to any adverse effects. If no alternative is possible: advise patients that, in the event of inadequate pain relief, they should contact the prescriber.
**1584**<br>**CYP2D6 IM: CODEINE**	The genetic polymorphism reduces the conversion of codeine into morphine. This can lead to a reduction in pain relief.<br><br>Recommendation:<br>* In the event of COUGHING:<br>- No action required<br><br>* In the event of PAIN:<br>Based on the limited amount of literature, it is not possible to make a sufficiently well substantiated recommendation with regard to dose adjustment for this phenotype.<br>- Be alert to reduced efficacy in the event of inadequate efficacy:<br>- Try increasing the dose; if this does not work select an alternative.<br>In this connection, do not select tramadol as this is also metabolized by CYP2D6.<br>Oxycodone is also metabolized by CYP2D6, but can usually be adjusted to give adequate pain relief without giving rise to any adverse effects.<br>If no alternative is selected: advise patients that, in the event of inadequate pain relief, they should contact the prescriber.
**1585**<br>**CYP2D6 UM: CODEINE**	The genetic polymorphism increases the conversion of codeine into morphine. This can lead to an increase in adverse effects. Mortality has occurred in children.<br><br>Recommendation:<br>Codeine is contraindicated with CYP2D6 UM.<br>If possible, select an alternative.<br>In the event of PAIN: do not select tramadol as this is also metabolized by CYP2D6.<br>Oxycodone is also metabolized by CYP2D6, but can usually be adjusted to give adequate pain relief without giving rise to any adverse effects.
**1586**<br>**CYP2D6 PM: OXYCODONE**	The genetic polymorphism reduces the conversion of oxycodone into the more active metabolite oxymorphone. However, when setting the dose based on pain, an adequate degree of pain relief is usually achieved.<br><br>Recommendation:<br>- Be alert to reduced pain relief
**1587**<br>**CYP2D6 IM: OXYCODONE**	The genetic polymorphism reduces the conversion of oxycodone into the more active metabolite oxymorphone. However, when setting the dose based on pain, an adequate degree of pain relief is

	usually achieved.<br><br>Recommendation:<br>- Be alert to reduced pain relief
**1588**<br>**CYP2D6 UM: OXYCODONE**	The genetic polymorphism increases the conversion of oxycodone into the more active metabolite oxymorphone. However, when setting the dose based on pain, an adequate degree of pain relief is usually achieved without any increase in adverse effects.<br><br>Recommendation:<br>- Be alert to the occurrence of adverse effects (such as drowsiness, confusion, constipation, nausea and vomiting, respiratory depression or urinary retention) and/or advise patients that, if they should experience any adverse effects they should contact the prescriber.
**1589**<br>**CYP2D6 PM: TRAMADOL**	The genetic polymorphism reduces the conversion of tramadol into a more active metabolite. This can lead to a reduction in pain relief.<br><br>Recommendation:<br>It is not possible to make a sufficiently well substantiated recommendation with regard to dose adjustment, as the total analgesic effect changes when the ratio between the parent compound and the active metabolite changes.<br>- Be alert to reduced efficacy in the event of inadequate efficacy<br>- Try increasing the dose; if this does not work select an alternative<br>In this connection, do not select codeine as this is also metabolized by CYP2D6.<br>Oxycodone is also metabolized by CYP2D6, but can usually be adjusted to give adequate pain relief without giving rise to any adverse effects.<br>* If no alternative is selected: advise patients that, in the event of inadequate pain relief, they should contact the prescriber.
**1590**<br>**CYP2D6 IM: TRAMADOL**	The genetic polymorphism reduces the conversion of tramadol into a more active metabolite. This can lead to a reduction in pain relief.<br><br>Recommendation:<br>It is not possible to make a sufficiently well substantiated recommendation with regard to dose adjustment, as the total analgesic effect changes when the ratio between the parent compound and the active metabolite changes.<br>- Be alert to reduced efficacy in the event of inadequate efficacy<br>- Try increasing the dose; if this does not work select an alternative<br>In this connection, do not select codeine as this is also metabolized by CYP2D6.<br>Oxycodone is also metabolized by CYP2D6, but can usually be adjusted to give adequate pain relief without giving rise to any adverse effects.<br>If no alternative is selected: advise patients that, in the event of inadequate pain relief, they should contact the prescriber.
**1591**<br>**CYP2D6 UM: TRAMADOL**	The genetic polymorphism increases the conversion of tramadol into a metabolite with stronger opioid activity. This can lead to an increase in adverse effects.<br><br>Recommendation:<br>The effect of a dose reduction cannot be predicted with any certainty as the total analgesic effect changes when the ratio between the parent compound and the active metabolite changes.<br>- Reduce the dose to 20%-40% of the normal dose<br>If a dose reduction fails to have the desired effect: select an alternative.<br>In this connection, do not select codeine as it is contraindicated with CYP2D6 UM.<br>Oxycodone is also metabolized by CYP2D6, but can usually be adjusted to give adequate pain relief without giving rise to any adverse effects.<br>If no alternative is selected: advise patients that, if they should experience adverse effects (such as drowsiness, confusion, constipation, nausea and vomiting, respiratory depression or urinary retention) they should contact the prescriber.
**1592**<br>**CYP2D6 PM: FLECAINIDE**	The genetic polymorphism leads to a reduction in CYP2D6's metabolic capacity, which can result in an increase in the plasma concentration of flecainide.<br><br>Recommendation:<br>- Reduce the dose to 50% of the normal dose, perform an ECG, and monitor the plasma concentration.
**1593**<br>**CYP2D6 IM: FLECAINIDE**	The genetic polymorphism leads to a reduction in CYP2D6's metabolic capacity, which can result in an increase in the plasma concentration of flecainide.<br><br>Recommendation:<br>- Reduce the dose to 75% of the normal dose, perform an ECG, and monitor the plasma concentration.
**1594**<br>**CYP2D6 UM: FLECAINIDE**	The genetic polymorphism leads to an increase in CYP2D6's metabolic capacity, which can result in a decrease in the plasma concentration of flecainide.<br><br>Recommendation:

	No data from studies into the pharmacokinetics and/or effects of flecainide in this phenotype have been published.<br>- As a precaution, monitor the plasma concentration and perform an ECG or select an alternative.<br>Some examples of antiarrhythmics that are only partially metabolized by CYP2D6, or not at all, are sotalol, disopyramide, quinidine and amiodarone.
**1595**<br>**CYP2D6 PM: PROPAFENONE**	The genetic polymorphism leads to a decrease in CYP2D6's metabolic capacity, which can result in an increase in the plasma concentration of propafenone, and a decrease in that of the active metabolite 5-hydroxypropafenone.<br><br>Recommendation:<br>- Reduce the dose to 30% of the normal dose, perform an ECG, and monitor the plasma concentration.
**1596**<br>**CYP2D6 IM: PROPAFENONE**	The genetic polymorphism leads to a decrease in CYP2D6's metabolic capacity, which can result in an increase in the plasma concentration of propafenone, and a decrease in that of the active metabolite 5-hydroxypropafenone.<br><br>Recommendation:<br>Based on the literature, it is not possible to make a sufficiently well substantiated recommendation with regard to dose adjustment.<br>- Either set the dose based on therapeutic drug monitoring, perform an ECG, and be alert to the occurrence of adverse effects,<br>- Or select an alternative.<br>Some examples of antiarrhythmics that are only partially metabolized by CYP2D6, or not at all, are sotalol, disopyramide, quinidine and amiodarone.
**1597**<br>**CYP2D6 UM: PROPAFENONE**	The genetic polymorphism leads to an increase in CYP2D6's metabolic capacity, which can result in a decrease in the plasma concentration of propafenone, and an increase in that of the active metabolite 5-hydroxypropafenone.<br>Recommendation:<br>Based on the literature, it is not possible to make a sufficiently well substantiated recommendation with regard to dose adjustment.<br>- Either monitor the plasma concentration, perform an ECG and be alert to reduced therapeutic efficacy,<br>- Or select an alternative.<br>Some examples of antiarrhythmics that are only partially metabolized by CYP2D6, or not at all, are sotalol, disopyramide, quinidine and amiodarone.
**1598**<br>**CYP2D6 PM: ATOMOXETINE**	The genetic polymorphism leads to a decrease in CYP2D6's metabolic capacity, which can result in an increase in the plasma concentration of atomoxetine and a decrease in that of the active metabolite.<br><br>Recommendation:<br>- Start with the normal starting dose, but make allowance for the fact that it will probably not be necessary to increase this dose. Advise patients that, if they should experience adverse effects (such as decreased appetite, vomiting, abdominal pain, constipation, insomnia, early awakening, drowsiness, irritability, pupil dilation and itching) they should contact the prescriber.
**1600**<br>**CYP2D6 UM: ATOMOXETINE**	The genetic polymorphism leads to an increase in CYP2D6's metabolic capacity, which can result in a decrease in the plasma concentration of atomoxetine and an increase in that of the active metabolite.<br><br>Recommendation:<br>There are no known data from studies into the pharmacokinetics and/or effects of atomoxetine in this phenotype.<br>- As a precaution, be extra alert to reduced therapeutic efficacy. Advise patients that, in the event of inadequate therapeutic efficacy, they should contact the prescriber. If necessary, as a precaution, an alternative can be selected.<br>Methylphenidate and clonidine are only partially metabolized by CYP2D6, or not at all.
**1601**<br>**CYP2D6 PM: TAMOXIFEN**	This genetic variant reduces the conversion of tamoxifen into its active metabolite endoxifen. This can reduce efficacy.<br><br>Recommendation:<br>- Select an alternative or increase the dose to 40 mg/day and monitor the endoxifen concentration.<br>Studies have shown that PM can achieve an adequate endoxifen concentration when the dose is increased to 40-60 mg/day.<br>For post-menopausal women, aromatase inhibitors are a potential alternative.
**1602**<br>**CYP2D6 IM: TAMOXIFEN**	This genetic variant reduces the conversion of tamoxifen into its active metabolite endoxifen. This can reduce efficacy.<br><br>Recommendation:<br>- Select an alternative or measure the endoxifen concentration and, if necessary, increase the dose by a factor of 1.5-2.<br>For post-menopausal women, aromatase inhibitors are a potential alternative.<br>- If TAMOXIFEN is selected: avoid concomitant therapy with CYP2D6 inhibitors, such as paroxetine or fluoxetine.

**1676**<br>**CYP2C9 IM: PHENYTOIN**	The genetic polymorphism leads to a reduction in CYP2C9's metabolic capacity, which can result in an increase in the plasma concentration of phenytoin.<br><br>Recommendation:<br>- A loading dose does not need to be adjusted. Reduce remaining doses to 75% of the normal dose, and assess the dose based on the effect and the serum level after 7-10 days. Advise patients that, if they should experience adverse effects (such as ataxia, nystagmus, speech disorder, sedation) they should contact the prescriber.
**1677**<br>**CYP2C9 PM: PHENYTOIN**	The genetic polymorphism leads to a reduction in CYP2C9's metabolic capacity, which can result in an increase in the plasma concentration of phenytoin.<br><br>Recommendation:<br>- A loading dose does not need to be adjusted. Reduce remaining doses to 50% of the normal dose, and assess the dose based on the effect and the serum level after 7-10 days. Advise patients that, if they should experience adverse effects (such as ataxia, nystagmus, speech disorder, sedation) they should contact the prescriber.
**1678 CYP2C9*1/*2: PHENYTOIN**	The genetic polymorphism leads to a reduction in CYP2C9's metabolic capacity, which can result in an increase in the plasma concentration of phenytoin.<br><br>Recommendation:<br>- A loading dose does not need to be adjusted. Reduce remaining doses to 75% of the normal dose, and assess the dose based on the effect and the serum level after 7-10 days. Advise patients that, if they should experience adverse effects (such as ataxia, nystagmus, speech disorder, sedation) they should contact the prescriber.
**1679**<br>**CYP2C9*1/*3: PHENYTOIN**	The genetic polymorphism leads to a reduction in CYP2C9's metabolic capacity, which can result in an increase in the plasma concentration of phenytoin.<br><br>Recommendation:<br>- A loading dose does not need to be adjusted. Reduce remaining doses to 75% of the normal dose, and assess the dose based on the effect and the serum level after 7-10 days. Advise patients that, if they should experience adverse effects (such as ataxia, nystagmus, speech disorder, sedation) they should contact the prescriber.
**1680**<br>**CYP2C9*2/*2: PHENYTOIN**	The genetic polymorphism leads to a reduction in CYP2C9's metabolic capacity, which can result in an increase in the plasma concentration of phenytoin.<br><br>Recommendation:<br>- A loading dose does not need to be adjusted. Reduce remaining doses to 50% of the normal dose, and assess the dose based on the effect and the serum level after 7-10 days. Advise patients that, if they should experience adverse effects (such as ataxia, nystagmus, speech disorder, sedation) they should contact the prescriber.
**1681**<br>**CYP2C9*2/*3: PHENYTOIN**	The genetic polymorphism leads to a reduction in CYP2C9's metabolic capacity, which can result in an increase in the plasma concentration of phenytoin.<br><br>Recommendation:<br>- A loading dose does not need to be adjusted. Reduce remaining doses to 50% of the normal dose, and assess the dose based on the effect and the serum level after 7-10 days. Advise patients that, if they should experience adverse effects (such as ataxia, nystagmus, speech disorder, sedation) they should contact the prescriber.
**1682**<br>**CYP2C9*3/*3: PHENYTOIN**	The genetic polymorphism leads to a reduction in CYP2C9's metabolic capacity, which can result in an increase in the plasma concentration of phenytoin.<br><br>Recommendation:<br>- A loading dose does not need to be adjusted. Reduce remaining doses to 50% of the normal dose, and assess the dose based on the effect and the serum level after 7-10 days. Advise patients that, if they should experience adverse effects (such as ataxia, nystagmus, speech disorder, sedation) they should contact the prescriber.
**1683**<br>**CYP2C19 IM: VORICONAZOL**	The genetic polymorphism leads to a reduction in CYP2C19's metabolic capacity, which can result in an increase in the plasma concentration of voriconazol.<br><br>Recommendation:<br>- Monitor the plasma concentration
**1684**<br>**CYP2C19 PM: VORICONAZOL**	The genetic polymorphism leads to a reduction in CYP2C19's metabolic capacity, which can result in an increase in the plasma concentration of voriconazol.<br><br>Recommendation:<br>- Monitor the plasma concentration

**1692**<br>**UGT1A1 PM: IRINOTECAN**	This genetic variant reduces the conversion of irinotecan to inactive metabolites. This increases the risk of severe, life-threatening adverse effects.<br><br>Recommendation:<br>- Reduce the starting dose by 30%<br>If the patient tolerates this starting dose, if necessary, the dose can be increased based upon the neutrophil count.
**1694**<br>**UGT1A1 *28/*28: IRINOTECAN**	This genetic variant reduces the conversion of irinotecan to inactive metabolites. This increases the risk of severe, life-threatening adverse effects.<br><br>Recommendation:<br>- Reduce the starting dose by 30%<br>If the patient tolerates this starting dose, if necessary, the dose can be increased based upon the neutrophil count.
**1820**<br>**CYP2C19 UM: ESCITALOPRAM**	The genetic polymorphism leads to an increase in CYP2C19's metabolic capacity, which can result in a decrease in the plasma concentration of escitalopram.<br><br>Recommendation:<br>- Be alert to a possible reduction in the plasma concentration of citalopram and, if necessary, increase the dose to 175% of the normal dose. If dose adjustment based on therapeutic drug monitoring is not possible, an alternative should be selected.<br>Some examples of antidepressants that are only partially metabolized by CYP2C19, or not at all, are fluoxetine and paroxetine.
**1821**<br>**CYP2C19 IM: ESCITALOPRAM**	The genetic polymorphism leads to an increase in the plasma concentrations of citalopram. This increases the risk of QT prolongation and torsades de pointes.<br><br>Recommendation:<br>- Prescribe up to the following maximum dose levels (50% of the normal maximum dose): adults up to the age of 65: 10 mg/day, and those over the age of 65: 5 mg/day.<br>While monitoring the ECG, the dose can be increased to the normal maximum dose, if necessary.
**1822**<br>**CYP2C19 PM: ESCITALOPRAM**	The genetic polymorphism leads to an increase in the plasma concentrations of citalopram. This increases the risk of QT prolongation and torsades de pointes.<br><br>Recommendation:<br>- Prescribe up to the following maximum doses (50% of the normal maximum dose): adults up to the age of 65: 10 mg/day, and those over the age of 65: 5 mg/day.
**1826**<br>**CYP2C19 UM: ESOMEPRAZOLE**	The genetic polymorphism leads to an increase in CYP2C19's metabolic capacity, which can result in a decrease in the plasma concentration of esomeprazole.<br><br>Recommendation:<br>In ERADICATION THERAPY for Helicobacter pylori:<br>- Increase the dose to 150-200% of the normal dose and advise patients to contact the prescriber if the dyspepsia problems persist.<br>* OTHER INDICATIONS:<br>- Look out for signs of reduced efficacy. If present, increase the dose to 150-200% of the normal dose. Advise patients to contact the prescriber if the dyspepsia problems persist.
**1833**<br>**CYP2C19 UM: LANSOPRAZOLE**	The genetic polymorphism leads to an increase in CYP2C19's metabolic capacity, which can result in a decrease in the plasma concentration of lansoprazole.<br><br>Recommendation:<br>In ERADICATION THERAPY for Helicobacter pylori:<br>- Increase the dose to 300% of the normal dose and advise patients to contact the prescriber if the dyspepsia problems persist.<br>* OTHER INDICATIONS:<br>- Look out for signs of reduced efficacy. If present, increase the dose to 300% of the normal dose. Advise patients to contact the prescriber if the dyspepsia problems persist.
**1841**<br>**CYP2C19 UM: OMEPRAZOLE**	The genetic polymorphism leads to an increase in CYP2C19's metabolic capacity, which can result in a decrease in the plasma concentration of omeprazole.<br><br>Recommendation:<br>In ERADICATION THERAPY for Helicobacter pylori:<br>- Increase the dose to 200-300% of the normal dose.<br>Advise patients to contact the prescriber if the dyspepsia problems persist.<br>* OTHER INDICATIONS:<br>- Look out for signs of reduced efficacy. If present, increase the dose to 200-300% of the normal dose.<br>Advise patients to contact the prescriber if the dyspepsia problems persist.

**1849**<br>**CYP2C19 UM: PANTOPRAZOLE**	The genetic polymorphism leads to an increase in CYP2C19's metabolic capacity, which can result in a decrease in the plasma concentration of pantoprazole.<br><br>Recommendation:<br>In ERADICATION THERAPY for Helicobacter pylori:<br>- Increase the dose to 500% of the normal dose and advise patients to contact the prescriber if the dyspepsia problems persist.<br>* OTHER INDICATIONS:<br>- Look out for signs of reduced efficacy. If present, increase the dose to 500% of the normal dose. Advise patients to contact the prescriber if the dyspepsia problems persist.
**1905**<br>**TPMT IM: AZATHIOPRINE / MERCAPTOPURINE**	The genetic variant reduces the conversion of azathioprine and mercaptopurine to largely inactive metabolites. This increases the risk of severe, life-threatening adverse effects, such as bone marrow suppression.<br><br>Recommendation:<br>- Reduce the starting dose to 50% of the normal dose.<br>Base the adjustment of the starting dose on toxicity (blood picture monitoring) and efficacy.<br>The frequency of monitoring should be increased.<br>If the current dose does not exceed azathioprine 1.5 mg/kg per day or mercaptopurine 0.75 mg/kg per day, no dose adjustment is necessary.
**1906**<br>**TPMT PM: AZATHIOPRINE / MERCAPTOPURINE**	The genetic variant reduces the conversion of azathioprine and mercaptopurine to largely inactive metabolites. This increases the risk of severe, life-threatening adverse effects, such as bone marrow suppression.<br><br>Recommendation:<br>Reduce the starting dose to 10% of the normal dose or select an alternative.<br>Base any adjustment of the starting dose on toxicity (blood picture monitoring) and efficacy.<br>The frequency of monitoring should be increased.<br>If the dose is reduced: advise patients to contact the prescriber if symptoms of bone marrow depression (e.g. sore throat in combination with elevated temperature, frequent nosebleeds and easy bruising) occur.
**1907**<br>**TPMT IM: TIOGUANINE**	The genetic variant reduces the conversion of tioguanine to inactive metabolites. This increases the risk of severe adverse effects, such as bone marrow suppression.<br><br>Recommendation:<br>- Reduce the starting dose to 75% of the normal dose.<br>Base the adjustment of the starting dose on toxicity (blood picture monitoring) and efficacy.<br>The frequency of monitoring should be increased.
**1908**<br>**TPMT PM: TIOGUANINE**	The genetic variant reduces the conversion of tioguanine to inactive metabolites. This increases the risk of severe, life-threatening adverse effects, such as bone marrow suppression.<br><br>Recommendation:<br>- Reduce the starting dose to 6-7% of the normal dose or select an alternative.<br>Base any adjustment of the starting dose on toxicity (blood picture monitoring) and efficacy.<br>The frequency of monitoring should be increased. If the dose is reduced: advise patients to contact the prescriber if symptoms of bone marrow depression (e.g. sore throat in combination with elevated temperature, frequent nosebleeds and easy bruising) occur.
**1910**<br>**VKORC1 TT: ACENOCOUMAROL**	The genetic variant leads to increased sensitivity to acenocoumarol.<br><br>Recommendation:<br>* Examination by the THROMBOSIS CLINIC:<br>- Recommend reducing the starting dose to 50% of the normal starting dose.<br>* OTHERWISE:<br>- Recommend reducing the starting dose to 50% of the normal starting dose.<br>- Recommend checking the INR more frequently. *<br>The starting dose and maintenance dose can be calculated using an algorithm, as used in EU-PACT.
**1912**<br>**VKORC1 TT: FENPROCOUMON**	The genetic variant leads to a lower dose being required. However, if the patient is regularly examined by the thrombosis clinic, there is no significant increase in the haemorrhage risk.<br><br>Recommendation:<br>* Examination by the THROMBOSIS CLINIC:<br>- Recommend reducing the starting dose to 50% of the normal starting dose.<br>* NO examination by the thrombosis clinic:<br>- Recommend reducing the starting dose to 50% of the normal starting dose.<br>- Check the INR more frequently. *<br>The starting dose and maintenance dose can be calculated using an algorithm, as used in EU-PACT.

**1914**<br>**CYP2C19 PM: IMIPRAMINE**	The genetic polymorphism leads to a reduction in CYP2C19's metabolic capacity, which can result in an increase in the plasma concentration of imipramine.<br><br>Recommendation:<br>- Reduce the dose to 70% of the normal dose and monitor the plasma concentration of imipramine and desipramine, or select an alternative.<br>Some examples of antidepressants that are only partially metabolized by CYP2C19, or not at all, are fluvoxamine and mirtazapine.
**1920**<br>**CYP2D6 IM: AMITRIPTYLINE**	The genetic polymorphism leads to a decrease in CYP2D6's metabolic capacity, which can result in an increase in the plasma concentrations of amitriptyline and its active metabolite nortriptyline, and a decrease in the plasma concentrations of the active metabolites E-10-OH-amitriptyline and E-10-OH-nortriptyline.<br><br>Recommendation:<br>If possible, select an alternative.<br>Some examples of antidepressants that are only partially metabolized by CYP2D6, or not at all, are citalopram and sertraline.<br>If no alternative is possible: reduce the dose to 60% of the normal dose and monitor the plasma concentrations of amitriptyline and nortriptyline.<br>Because the adverse effects are related to the nortriptyline plasma concentration and the efficacy is related to the plasma concentration of amitriptyline plus nortriptyline, which is not influenced as strongly by CYP2D6, it is not known whether it is possible to reduce the dose to a point at which the adverse effects disappear while efficacy is maintained.
**1921**<br>**CYP2D6 PM: AMITRIPTYLINE**	The genetic polymorphism leads to a decrease in CYP2D6's metabolic capacity, which can result in an increase in the plasma concentrations of amitriptyline and its active metabolite nortriptyline, and a decrease in the plasma concentrations of the active metabolites E-10-OH-amitriptyline and E-10-OH-nortriptyline.<br><br>Recommendation:<br>If possible, select an alternative.<br>Some examples of antidepressants that are only partially metabolized by CYP2D6, or not at all, are citalopram and sertraline.<br>If no alternative is possible:<br>- Reduce the dose to 50% of the normal dose and monitor the plasma concentrations of amitriptyline and nortriptyline.<br>Because the adverse effects are related to the nortriptyline plasma concentration and the efficacy is related to the plasma concentration of amitriptyline plus nortriptyline, which is not influenced as strongly by CYP2D6, it is not known whether it is possible to reduce the dose to a point at which the adverse effects disappear while efficacy is maintained.
**1922**<br>**CYP2D6 UM: AMITRIPTYLINE**	The genetic polymorphism leads to an increase in CYP2D6's metabolic capacity, which can result in a decrease in the plasma concentrations of amitriptyline and its active metabolite nortriptyline, and an increase in the plasma concentrations of the active metabolites E-10-OH-amitriptyline and E-10-OH-nortriptyline.<br><br>Recommendation:<br>If possible, select an alternative.<br>Some examples of antidepressants that are only partially metabolized by CYP2D6, or not at all, are citalopram and sertraline.<br>If no alternative is possible:<br>- Increase the dose to 125% of the normal dose, monitor the plasma concentration and be alert to the possibility of therapy failing due to a reduction in the plasma concentrations of amitriptyline and nortriptyline and an increase in the plasma concentration of the potentially cardiotoxic, active hydroxy metabolites.
**2008**<br>**CYP2C19 IM: SERTRALINE**	The genetic polymorphism leads to a reduction in CYP2C19's metabolic capacity, which can result in an increase in the plasma concentrations of sertraline and desmethylsertraline.<br>Recommendation:<br>- Do not use a dose higher than 100 mg/day. Adjust the dose on the basis of the response and adverse effects and/or on the basis of the plasma concentration of sertraline plus desmethylsertraline.
**2009**<br>**CYP2C19 PM: SERTRALINE**	The genetic polymorphism leads to a reduction in CYP2C19's metabolic capacity, which can result in an increase in the plasma concentrations of sertraline and desmethylsertraline.<br><br>Recommendation:<br>- Do not use a dose higher than 50 mg/day. Adjust the dose on the basis of the response and adverse effects and/or on the basis of the plasma concentration of sertraline plus desmethylsertraline.
**2015**<br>**CYP2D6 IM: DOXEPIN**	The genetic polymorphism leads to a reduction in CYP2D6's metabolic capacity, which can result in an increase in the plasma concentrations of doxepin and nordoxepin.

	Recommendation:<br>- Reduce the dose to 80% of the normal dose and monitor the plasma concentrations of doxepin and nordoxepin for the purpose of setting the maintenance dose.
**2016**<br>**CYP2D6 PM: DOXEPIN**	The genetic polymorphism leads to a reduction in CYP2D6's metabolic capacity, which can result in an increase in the plasma concentrations of doxepin and nordoxepin.<br><br>Recommendation:<br>- Reduce the dose to 40% of the normal dose and monitor the plasma concentrations of doxepin and nordoxepin for the purpose of setting the maintenance dose.
**2017**<br>**CYP2D6 UM: DOXEPIN**	The genetic polymorphism leads to an increase in CYP2D6's metabolic capacity, which can result in a decrease in the plasma concentrations of doxepin and nordoxepin, and an increase in the plasma concentrations of the hydroxy metabolites.<br><br>Recommendation:<br>- Select an alternative.<br>Some examples of antidepressants that are only partially metabolized by CYP2D6, or not at all, are citalopram and sertraline. If no alternative is possible, increase the dose to 200% of the normal dose and monitor the plasma concentrations of doxepin and nordoxepin for the purpose of setting the maintenance dose.
**2357**<br>**CYP3A5 HOMOZYGOUS EXPRESSOR: TACROLIMUS**	The genetic variant leads to increased conversion of tacrolimus to inactive metabolites, making a higher dose necessary. Adjustment of the starting dose increases the probability of the tacrolimus concentration being within the target range prior to the start of therapeutic drug monitoring on day 3. However, there is no direct evidence of that leading to better clinical outcomes.<br><br>Recommendation:<br>* Indications OTHER than liver transplantation:<br>- Increase the starting dose to 250% of the starting dose associated with the desired outcome in non-expressors.<br>Subsequent adjustment of the dose should be based on therapeutic drug monitoring.<br>NB: The starting dose associated with the desired outcome in non-expressors may be lower than the normal starting dose. In the example below, the starting dose associated with the desired outcome in non-expressors was 75% of the normal starting dose. Therefore, increasing the starting dose to 250% of the starting dose associated with the desired outcome in non-expressors implies increasing it to 200% of the normal starting dose. Example: After 3 days, Thervet et al. observed a median trough tacrolimus concentration of 14.0 ng/ml at a starting dose of 0.15 mg/kg 2x per day in 4 renal transplant patients who were homozygous expressors. At a starting dose of 0.1 mg/kg 2x per day, the concentration was 5.6 ng/ml (n = 6). Their target value was 10-15 ng/ml. That is lower than the target value sought in the Netherlands in the first 2-4 weeks following renal transplantation (15-20 ng/ml). In the reference group of non-expressors, median trough concentrations of 16.6 ng/ml and 12,0 ng/ml were observed at doses of, respectively 0.1 mg/kg 2x per day and 0.075 mg/kg 2x per day. In this hospital, the first dose is administered to patients whose CYP3A5 genotype is not known. The second dose is a reduced dose based on the patient's genotype.<br><br>* LIVER transplantation:<br>The metabolism of tacrolimus is influenced not only by the patient's genotype, but also by the genotype of the transplanted liver.<br>* LIVER genotype is also HOMOZYGOUS EXPRESSOR:<br>- Increase the starting dose to 250% of the normal starting dose.<br>Subsequent adjustment of the dose should be based on therapeutic drug monitoring.<br>* LIVER is ANOTHER genotype:<br>The literature provides insufficient information to support a dosage recommendation.
**2358**<br>**CYP3A5 HETEROZYGOUS EXPRESSOR: TACROLIMUS**	The genetic variant leads to increased conversion of tacrolimus to inactive metabolites, making a higher dose necessary. Adjustment of the starting dose increases the probability of the tacrolimus concentration being within the target range prior to the start of therapeutic drug monitoring on day 3. However, there is no direct evidence of that leading to better clinical outcomes.<br><br>* Indications OTHER than liver transplantation:<br>- Increase the starting dose to 175% of the starting dose associated with the desired outcome in non-expressors.<br>Subsequent adjustment of the dose should be based on therapeutic drug monitoring.<br>NB: The starting dose associated with the desired outcome in non-expressors may be lower than the normal starting dose. In the example below, the starting dose associated with the desired outcome in non-expressors was 75% of the normal starting dose. Therefore, increasing the starting dose to 175% of the starting dose associated with the desired outcome in non-expressors implies increasing it to 130% of the normal starting dose. Example: After 3 days, Thervet et al. observed a median trough tacrolimus concentration of 12.3 ng/ml at a starting dose of 0.15 mg/kg 2x per day in heterozygous renal transplant patients. Their target value was 10-15 ng/ml. That is lower than the target value sought in the Netherlands in the first 2-4 weeks following renal transplantation (15-20 ng/ml). In the reference group of non-expressors, median trough concentrations of 16.6 ng/ml and 12,0 ng/ml were observed at doses

	of, respectively 0.1 mg/kg 2x per day and 0.075 mg/kg 2x per day. In this hospital, the first dose is administered to patients whose CYP3A5 genotype is not known. The second dose is a reduced dose based on the patient's genotype.<br><br>* LIVER transplantation:<br>The metabolism of tacrolimus is influenced not only by the patient's genotype, but also by the genotype of the transplanted liver.<br>* LIVER genotype is also HETEROZYGOUS EXPRESSOR:<br>- Increase the starting dose to 175% of the normal starting dose.<br>Subsequent adjustment of the dose should be based on therapeutic drug monitoring.<br>* LIVER is ANOTHER genotype:<br>The literature provides insufficient information to support a dosage recommendation.
**2447**<br>**CYP2D6 PM: PIMOZIDE**	The genetic variant leads to an increase in the plasma concentration of pimozide. That increases the risk of potentially life-threatening QT prolongation.<br><br>Recommendation:<br>- Prescribe up to the following maximum doses (25% of the normal maximum dose): adults 5 mg/day, children 0.025 mg/kg per day, up to a maximum of 1 mg/day.
**2448**<br>**CYP2D6 IM: PIMOZIDE**	The genetic variant leads to an increase in the plasma concentration of pimozide. That increases the risk of potentially life-threatening QT prolongation.<br><br>Recommendation:<br>- Prescribe up to the following maximum doses (60-65% of the normal maximum dose): adults 12 mg/day, children 0.065 mg/kg per day, up to a maximum of 2.6 mg/day.
**2548**<br>**CYP2C19 PM: CLOPIDOGREL**	The genetic polymorphism leads to a decrease in CYP2C19's metabolic capacity, which can result in a decrease in the plasma concentration of the active metabolite of clopidogrel. Patients with this genetic polymorphism are at elevated risk of a poor clopidogrel response.<br><br>Recommendation:<br>- Consider an alternative.<br>Prasugrel and ticagrelor are only partially metabolized by CYP2C19, or not at all.
**2549**<br>**CYP2C19 IM: CLOPIDOGREL**	The genetic polymorphism leads to a decrease in CYP2C19's metabolic capacity, which can result in a decrease in the plasma concentration of the active metabolite of clopidogrel. Patients with this genetic polymorphism are at elevated risk of a poor clopidogrel response.<br><br>Recommendation:<br>- Consider an alternative.<br>Prasugrel and ticagrelor are only partially metabolized by CYP2C19, or not at all.
**2551**<br>**DPD GENACT 0: FLUOROURACIL / CAPECITABINE**	The genetic variant affects DPD, leading to the reduced conversion of fluorouracil/capecitabine to inactive metabolites. This may cause intracellular concentrations of the active metabolite of fluorouracil/capecitabine to increase, leading to a greater risk of serious, potentially fatal, toxicity.<br><br>Recommendation:<br>- Select an alternative.<br>Tegafur is not an alternative, as this is also metabolized by DPD.<br>NB: If a patient has two different genetic variants that have resulted in an inactive DPD enzyme (e.g. *2A and *13), this recommendation will only apply if the variants are located on different alleles. If both variants are located on the same allele, the patient will have a gene activity score of 1, and the recommendation associated with this score should be followed. The only way to distinguish between these two situations is to measure the activity of the enzyme (phenotyping).
**2552**<br>**DPD GENACT 1: FLUOROURACIL / CAPECITABINE**	The genetic variant affects DPD, leading to the reduced conversion of fluorouracil/capecitabine to inactive metabolites. This may cause intracellular concentrations of the active metabolite of fluorouracil/capecitabine to increase, leading to a greater risk of serious, potentially fatal, toxicity.<br><br>Recommendation:<br>- Reduce the starting dose to 50% of the normal dose or select an alternative.<br>Base the adjustment of the starting dose on toxicity and efficacy. Tegafur is not an alternative, as this is also metabolized by DPD.<br>NB1: Reduction of the dose as described is well-supported for *1/*2A. In patients with 2 alleles associated with reduced activity (e.g. 2846T/2846T or 1236A/1236A), the dose reduction is calculated from the dose reduction observed for *1/2846T.<br>NB2: If a patient has two different genetic variants associated with a less active DPD enzyme (e.g. 2846T and 1236A), the recommendation above applies only if the variants are located on different alleles. If both variants are located on the same allele, the patient will have a gene activity score of 1.5, and the recommendation associated with that score should be followed. The only way to distinguish between these two situations is to measure the activity of the enzyme (phenotyping).

**2553**<br>**DPD GENACT 0: TEGAFUR**	This genetic variant reduces the conversion of tegafur to inactive metabolites. That increases the risk of severe, potentially fatal toxicity.<br><br>Recommendation:<br>Tegafur is contraindicated in patients with a gene activity score of 0 (zero).<br>- Select an alternative.<br>Do not select fluorouracil or capecitabine, as these are also metabolized by DPD.<br>NB: If a patient has two different genetic variants that have resulted in an inactive DPD enzyme (e.g. *2A and *13), this recommendation will only apply if the variants are located on different alleles. If both variants are located on the same allele, the patient will have a gene activity score of 1, and the recommendation associated with this score should be followed. The only way to distinguish between these two situations is to measure the activity of the enzyme (phenotyping).
**2554**<br>**DPD GENACT 1: TEGAFUR**	This genetic variant reduces the conversion of tegafur to inactive metabolites. That increases the risk of the normal dose leading to severe, potentially fatal toxicity.<br><br>Recommendation:<br>- Select an alternative or start with a reduced dose and adjust the starting dose on the basis of toxicity and efficacy.<br>Fluorouracil and capecitabine are not usable alternatives, as they are also metabolized by DPD. The literature does not provide an adequate basis on which to make a recommendation regarding dose adjustment. It is recommended that the starting doses of fluorouracil and capecitabine be reduced to 50% of the normal dose.<br>NB: If a patient has two different genetic variants associated with a less active DPD enzyme (e.g. 2846T and 1236A), the recommendation above applies only if the variants are located on different alleles. If both variants are located on the same allele, the patient will have a gene activity score of 1.5, and the recommendation associated with that score should be followed. The only way to distinguish between these two situations is to measure the activity of the enzyme (phenotyping).
**4055**<br>**SLCO1B1 521TC: SIMVASTATIN**	The genetic polymorphism can lead to reduced transportation of simvastatin to the liver. That can result in an elevated simvastatin plasma concentration and thus to an increased risk of myopathy.<br><br>Recommendation:<br>- Select an alternative.<br>When selecting an alternative, consider any additional risk factors for statin-induced myopathy that may be present.<br>Rosuvastatin and pravastatin are not influenced as strongly by SLCO1B1 polymorphisms. Nor are they influenced by CYP3A4 inhibitors, such as amiodarone, verapamil and diltiazem. Fluvastatin is not influenced by SLCO1B1 polymorphisms or CYP3A4-inhibitors.<br>If no alternative is possible:<br>- Avoid simvastatin doses exceeding 40 mg/day and advise patients to contact the prescriber in the event of muscle problems.
**4056**<br>**SLCO1B1 521CC: SIMVASTATIN**	The genetic polymorphism leads to reduced transportation of simvastatin to the liver. That results in an elevated simvastatin plasma concentration and thus to an increased risk of myopathy.<br><br>Recommendation:<br>- Select an alternative.<br>When selecting an alternative, consider any additional risk factors for statin-induced myopathy that may be present.<br>Rosuvastatin and pravastatin are not influenced as strongly by SLCO1B1 polymorphisms. Nor are they influenced by CYP3A4 inhibitors, such as amiodarone, verapamil and diltiazem. Fluvastatin is not influenced by SLCO1B1 polymorphisms or CYP3A4-inhibitors.
**4057**<br>**SLCO1B1 521TC: ATORVASTATIN**	The genetic polymorphism can lead to reduced transportation of atorvastatin to the liver. That can result in an elevated atorvastatin plasma concentration and thus to an increased risk of myopathy.<br><br>Recommendation:<br>* Patient has SIGNIFICANT ADDITIONAL RISK FACTORS for statin-induced myopathy:<br>- Select an alternative.<br>Rosuvastatin and pravastatin are influenced by SLCO1B1 polymorphisms to a broadly similar extent, but they are not influenced by CYP3A4-inhibitors, such as amiodarone, verapamil and diltiazem.<br>Fluvastatin is not influenced by SLCO1B1 polymorphisms or CYP3A4-inhibitors. If no alternative is possible, advise patients to contact the prescriber in the event of muscle problems.<br>* Patient has NO significant additional risk factors for statin-induced myopathy:<br>- Advise patients to contact the prescriber in the event of muscle problems.
**4058**<br>**SLCO1B1 521CC: ATORVASTATIN**	The genetic polymorphism can lead to reduced transportation of atorvastatin to the liver. That can result in an elevated atorvastatin plasma concentration and thus to an increased risk of myopathy.<br><br>Recommendation:<br>* Patient has SIGNIFICANT ADDITIONAL RISK FACTORS for statin-induced myopathy:<br>- Select an alternative.

	Rosuvastatin and pravastatin are influenced by SLCO1B1 polymorphisms to a broadly similar extent, but they are not influenced by CYP3A4-inhibitors, such as amiodarone, verapamil and diltiazem.<br>Fluvastatin is not influenced by SLCO1B1 polymorphisms or CYP3A4-inhibitors.<br>If no alternative is possible:<br>- Advise patients to contact the prescriber in the event of muscle problems.<br>* Patient has NO significant additional risk factors for statin-induced myopathy:<br>- Advise patients to contact the prescriber in the event of muscle problems.
**4195**<br>**CYP2C19 IM: CITALOPRAM**	The genetic polymorphism leads to an increase in the plasma concentrations of citalopram. This increases the risk of QT prolongation and torsades de pointes.<br><br>Recommendation:<br>- Prescribe up to the following maximum daily doses:<br>* Adults up to 65 years old: 20 mg in tablet form or 22 mg in droplet form From 65 years old: 10 mg in tablet form or droplet form<br>While monitoring the ECG, the dose may if necessary be increased to the normal maximum dose:<br>* Adults up to 65 years old: 40 mg in tablet form or 32 mg in droplet form From 65 years old: 20 mg in tablet form or 16 mg in droplet form
**4196**<br>**CYP2C19 PM: CITALOPRAM**	The genetic polymorphism leads to an increase in the plasma concentrations of citalopram. That increases the risk of QT prolongation and torsades de pointes.<br><br>Recommendation:<br>- Prescribe up to the following maximum doses (50% of the normal maximum doses):<br>* Adults up to 65 years old: 20 mg in tablet form or 16 mg in droplet form From 65 years old: 10 mg in tablet form or 8 mg in droplet form
**4642**<br>**CYP1A2*1C-HETEROZYGOUS: CLOZAPINE**	The genetic polymorphism can lead to reduced metabolism of clozapine. That can result in an elevated clozapine plasma concentration and thus to an increased risk of adverse effects.<br><br>Recommendation:<br>The literature does not provide an adequate basis on which to make a recommendation regarding dose reduction.<br>- Adjust the dose on the basis of the plasma concentration of clozapine.<br>The therapeutic range suggested for clozapine is 200-600 ng/ml.
**4643**<br>**CYP1A2*1C/*1C: CLOZAPINE**	The genetic polymorphism can lead to reduced metabolism of clozapine. That can result in an elevated clozapine plasma concentration and thus to an increased risk of adverse effects.<br>Recommendation:<br>The literature does not provide an adequate basis on which to make a recommendation regarding dose reduction.<br>- Adjust the dose on the basis of the plasma concentration of clozapine.<br>The therapeutic range suggested for clozapine is 200-600 ng/ml.
**4652**<br>**HLA-B*5701: FLUCLOXACILLIN**	HLA-B*5701-positive patients are at 80 times greater risk of flucloxacillin-induced liver damage. The incidence is nevertheless low (1-2 cases per 1000 people).<br><br>Recommendation:<br>- Check the patient's liver function regularly and select an alternative if liver enzymes and/or bilirubin increase.
**4754**<br>**CYP2B6 IM: EFAVIRENZ**	The genetic variant can lead to reduced metabolism of efavirenz. This can result in an elevated efavirenz plasma concentration and thus to an increased risk of adverse effects. For the majority of the IM, however, the efavirenz plasma concentration remains within the therapeutic range.<br><br>Recommendation:<br>- If adverse effects occur, check the efavirenz plasma concentration and, if necessary, reduce the dose to 400 or 200 mg/day.<br>The therapeutic range determined for efavirenz is 1000-4000 ng/ml.
**4755**<br>**CYP2B6 PM: EFAVIRENZ**	The genetic variant can lead to reduced metabolism of efavirenz. This can result in an elevated efavirenz plasma concentration and thus to an increased risk of adverse effects.<br><br>Recommendation:<br>* Efavirenz in MONO preparation form:<br>*Body mass index NOT EXCEEDING 25: - Start with a dose of 400 mg/day and thereafter adjust the dose as necessary, on the basis of the plasma concentration (reduce further to 200 mg/day or, exceptionally, increase to 600 mg/day).<br>The therapeutic range determined for efavirenz is 1000-4000 ng/ml.<br>* Body Mass Index MORE THAN 25:<br>- Start with a dose of 600 mg/day and thereafter adjust the dose as necessary, on the basis of the plasma concentration (reduce to 400 or 200 mg/day).<br>The therapeutic range determined for efavirenz is 1000-4000 ng/ml.<br><br>* Efavirenz in COMBINATION preparation form:

	- Start with the combination preparation and thereafter adjust the efavirenz dose as necessary, on the basis of the plasma concentration (reduce to 400 or 200 mg/day).<br>The therapeutic range determined for efavirenz is 1000-4000 ng/ml.<br><br>Considerations:<br>Excluding studies carried out in Africa, the median of the average plasma concentration for PM is above the therapeutic range. A recent study observed a virological response to efavirenz at 400 and 600 mg/day that was similar to that observed in patients not selected on the basis of genotype. The risk of an underdose resulting from reduction of the starting dose to 400 mg/day is very small, therefore.<br>Both administration in combination preparation form and the avoidance of unnecessary adverse effects associated with an excessive plasma concentration promote therapy adherence.<br>With PM, it is not necessary to take account of the possible use of CYP2B6-inductors, such as rifampicin. Because CYP2B6's metabolic capacity is greatly reduced or entirely lacking, induction is of little or no relevance.
**4891**<br>**DPD GENACT 0.5: TEGAFUR**	This genetic variant reduces the conversion of tegafur to inactive metabolites. That increases the risk of the normal dose leading to severe, potentially fatal toxicity.<br><br>Recommendation:<br>The literature does not provide an adequate basis on which to make a recommendation regarding dose reduction.<br>- Select an alternative or start with a low dose and adjust the starting dose on the basis of toxicity and efficacy.<br>Fluorouracil and capecitabine are not usable alternatives, as they are also metabolized by DPD. The literature does not provide an adequate basis on which to make a recommendation regarding dose adjustment. It is recommended that the starting doses of fluorouracil and capecitabine be reduced to 25% of the normal dose.<br>NB: This recommendation will only apply if the two genetic variants are located on different alleles. If both variants are located on the same allele, the patient will have a gene activity score of 1, and the recommendation associated with this score should be followed. The only way to distinguish between these two situations is to measure the activity of the enzyme (phenotyping).
**4892**<br>**DPD GENACT 1.5: TEGAFUR**	This genetic variant reduces the conversion of tegafur to inactive metabolites. That increases the risk of the normal dose leading to severe, potentially fatal toxicity.<br><br>Recommendation:<br>- Select an alternative or start with a reduced dose and adjust the starting dose on the basis of toxicity and efficacy.<br>Fluorouracil and capecitabine are not usable alternatives, as they are also metabolized by DPD.<br>The literature does not provide an adequate basis on which to make a recommendation regarding dose adjustment. It is recommended that the starting doses of fluorouracil and capecitabine be reduced to 75% of the normal dose.
**4893**<br>**DPD GENACT 0.5: FLUOROURACIL / CAPECITABINE**	The genetic variant affects DPD, leading to the reduced conversion of fluorouracil/capecitabine to inactive metabolites. This may cause intracellular concentrations of the active metabolite of fluorouracil/capecitabine to increase, leading to a greater risk of serious, potentially fatal, toxicity.<br><br>Recommendation:<br>- Reduce the starting dose to 25% of the normal dose or select an alternative.<br>Base the adjustment of the starting dose on toxicity and efficacy. Tegafur is not an alternative, as this is also metabolized by DPD.<br>NB: This recommendation will only apply if the two genetic variants are located on different alleles. If both variants are located on the same allele, the patient will have a gene activity score of 1, and the recommendation associated with this score should be followed. The only way to distinguish between these two situations is to measure the activity of the enzyme (phenotyping).
**4894**<br>**DPD GENACT 1.5: FLUOROURACIL / CAPECITABINE**	The genetic variant affects DPD, leading to the reduced conversion of fluorouracil/capecitabine to inactive metabolites. This may cause intracellular concentrations of the active metabolite of fluorouracil/capecitabine to increase, leading to a greater risk of serious, potentially fatal, toxicity.<br><br>Recommendation:<br>- Reduce the starting dose to 75% of the normal dose or select an alternative.<br>Base the adjustment of the starting dose on toxicity and efficacy. Tegafur is not an alternative, as this is also metabolized by DPD.
**6233 - CYP2C9 IM: WARFARIN**	This gene variation reduces the conversion of warfarin to inactive metabolites. This can increase the risk of bleeding.<br><br>Recommendation:<br><br>1. use 65% of the standard initial dose<br><br>The genotype-specific initial dose and maintenance dose can be calculated using an algorithm.

	Algorithms for Caucasian patients usually contain only the *2 and *3 allele. If the activity of the reduced-activity alleles is comparable to the activity of *2 or *3, then the algorithm can be completed as if *1/*2 or *1/*3 is present. See https://www.knmp.nl/patientenzorg/medicatiebewaking/farmacogenetica for Excel files containing calculation modules for oral and equivalent intravenous doses. From day 6 on the standard algorithm without genotype information can be used to calculate the dose.<br>Modified dose algorithms have been developed for patients of African or (East) Asian heritage.
**6234 - CYP2C9 PM: WARFARIN**	This gene variation reduces the conversion of warfarin to inactive metabolites. This can increase the risk of bleeding.<br><br>Recommendation:<br><br>1. use 20% of the standard initial dose<br><br>The genotype-specific initial dose and maintenance dose can be calculated using an algorithm. Algorithms for Caucasian patients usually contain only the *2 and *3 allele. If the activity of the reduced-activity alleles is comparable to the activity of *2 or *3, then the algorithm can be completed as if *2 or *3 is present. See https://www.knmp.nl/patientenzorg/medicatiebewaking/farmacogenetica for Excel files containing calculation modules for oral and equivalent intravenous doses. From day 6 on the standard algorithm without genotype information can be used to calculate the dose.<br>Modified dose algorithms have been developed for patients of African or (East) Asian heritage.
**6228 - CYP2C9*1/*2: WARFARIN**	NO action is required for this gene-drug interaction.<br><br>Genetic variation may lead to a decrease in the required maintenance dose. However, there is insufficient evidence that this causes problems when therapy is initiated as usual.
**6229 - CYP2C9*1/*3: WARFARIN**	This gene variation reduces the conversion of warfarin to inactive metabolites. This can increase the risk of bleeding.<br><br>Recommendation:<br><br>1. use 65% of the standard initial dose<br><br>The genotype-specific initial dose and maintenance dose can be calculated using an algorithm, as used in EU-PACT: see https://www.knmp.nl/patientenzorg/medicatiebewaking/farmacogenetica<br>From day 6 on the standard algorithm without genotype information can be used to calculate the dose.
**6230 - CYP2C9*2/*2: WARFARIN**	This gene variation reduces the conversion of warfarin to inactive metabolites. This can increase the risk of bleeding.<br><br>Recommendation:<br><br>1. use 65% of the standard initial dose<br><br>The genotype-specific initial dose and maintenance dose can be calculated using an algorithm, as used in EU-PACT: see https://www.knmp.nl/patientenzorg/medicatiebewaking/farmacogenetica<br>From day 6 on the standard algorithm without genotype information can be used to calculate the dose.
**6231 - CYP2C9*2/*3: WARFARIN**	This gene variation reduces the conversion of warfarin to inactive metabolites. This can increase the risk of bleeding.<br><br>Recommendation:<br><br>1. use 45% of the standard initial dose<br><br>The genotype-specific initial dose and maintenance dose can be calculated using an algorithm, as used in EU-PACT: see https://www.knmp.nl/patientenzorg/medicatiebewaking/farmacogenetica.<br>From day 6 on the standard algorithm without genotype information can be used to calculate the dose.
**6232 - CYP2C9*3/*3: WARFARIN**	This gene variation reduces the conversion of warfarin to inactive metabolites. This can increase the risk of bleeding.<br><br>Recommendation:<br><br>1. use 20% of the standard initial dose<br><br>The genotype-specific initial dose and maintenance dose can be calculated using an algorithm, as used in EU-PACT: see https://www.knmp.nl/patientenzorg/medicatiebewaking/farmacogenetica<br>From day 6 on the standard algorithm without genotype information can be used to calculate the dose.
**6235 – VKORC1 CT: WARFARIN**	NO action is required for this gene-drug interaction.

	The genetic variation results in a reduction in the required dose and an increase in the risk of excessively severe inhibition of blood clotting during the first month of the treatment. However, the effect is small and CT is also the most common genotype, meaning that the standard treatment will primarily be based on patients with this genotype.
**6236 – VKORC1 TT: WARFARIN**	The genetic variation results in increased sensitivity to warfarin. This results in an increase in the risk of excessively severe inhibition of blood clotting (INR >4) during the first month of the treatment.<br><br>Recommendation:<br><br>1. use 60% of the standard initial dose<br><br>The genotype-specific initial dose and maintenance dose can be calculated using an algorithm, as used in EU-PACT: see https://www.knmp.nl/patientenzorg/medicatiebewaking/farmacogenetica.<br>From day 6 on the standard algorithm without genotype information can be used to calculate the dose.

18.4. Scientific Advisory Board

Prominent clinicians and scientists representing key aspects of the U-PGX proposal have accepted to participate in the Scientific Advisory Board (SAB) of U-PGx. Project execution will be continuously advised and critically reflected by this strong advisory board.

1. ***Mark J. Ratain, MD (Chair)***

Professor & Chairman of Center for Personalised Therapeutics, University of Chicago Medicine, Chicago, USA. Main expertise: Pharmacology

2. ***Russ Altman, MD***

Kenneth Fong Professor and Professor of Bioengineering of Genetics, of Medicine (General Medical Discipline) and, by courtesy, of Computer Science, Stanford University at San Paulo, USA. Main expertise: Bioinformatics

3. ***Dan Roden, MD***

Professor of Pharmacology, Assistant Vice Chancellor for Personalised Medicine, Professor of Medicine, William Stokes Chair in Experimental Therapeutics, Vanderbilt University, Nashville, USA. Main expertise: Pharmacology

4. ***Michel Eichelbaum, MD***

Professor & former chair of the Dr. Margarete Fischer-Bosch-Institute of Clinical Pharmacology, Stuttgart/Germany, Professor and former chairman of Clinical Pharmacology at the University of Tübingen, Germany. Main expertise: Pharmacology/Pharmacogenetics

5. ***David H.U. Haerry***

David Haerry is a patients' advocate. He is co-chair of the Patient and Consumer Working Party of the European Medicines Agency (EMA).

6. ***Mary Relling PharmD***

Scientist at St. Jude Children's Research Hospital where she is a member of the St. Jude Faculty, chair of the Pharmaceutical Sciences Department and leader of the Clinical Pharmacogenetics Implementation Consortium (CPIC) Initiative.

Tasks: The Scientific Advisory Board will ensure a high standard of research and monitor the progress of the project by taking part in the annual General Assembly Meetings. Whenever appropriate, the

consortium will consult the Advisory Board for recommendations to improve the performance of the consortium.

18.5. Organogram of U-PGx Consortium

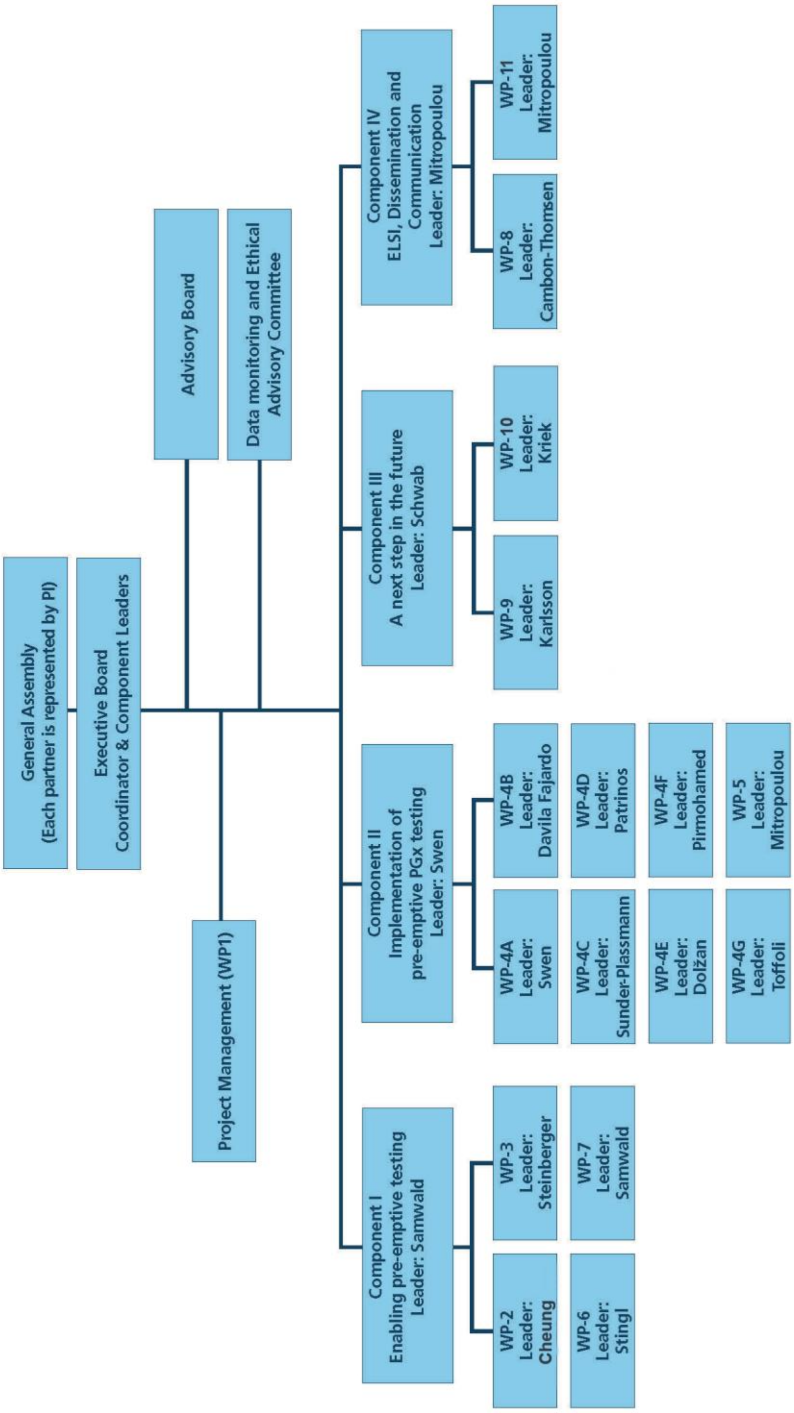

Supplement: Supplement 1. — Trial Protocol [file jamanetwopen-e2449441-s001.pdf]
