# Supplementary material for: Clinical Benefits and Utility of Pretherapeutic DPYD and UGT1A1 Testing in Gastrointestinal Cancer: A Secondary Analysis of the PREPARE Randomized Clinical Trial
Source: JAMA Netw Open. 2024 Dec 6;7(12):e2449441. doi: 10.1001/jamanetworkopen.2024.49441 (PMC11624585; doi:10.1001/jamanetworkopen.2024.49441)
Supplement: Supplement 2. — eMethods. Detailed Methods eResults. Detailed Results eFigure 1. Frequency of Toxicity According to Grade and Causality and Hospitalization Costs eFigure 2. Swimmer Plot: Case-by-Case Description of Treatment Adherence for Actionable Genotypes Carriers eFigure 3. Crossed Drug/Gene Comparison of Dose Density eFigure 4. Quality-Adjusted Life Years eReferences [file jamanetwopen-e2449441-s002.pdf]

Supplementary Online Content

Roncato R, Bignucolo A, Peruzzi E, et al. Clinical benefits and utility of pretherapeutic *DPYD* and *UGT1A1* testing in gastrointestinal cancer: a secondary analysis of the PREPARE randomized clinical trial. *JAMA Netw Open*. 2024;7(12):e2449441. doi:10.1001/jamanetworkopen.2024.49441

eMethods. Detailed Methods

eResults. Detailed Results

eFigure 1. Frequency of Toxicity According to Grade and Causality and Hospitalization Costs

eFigure 2. Swimmer Plot: Case-by-Case Description of Treatment Adherence for Actionable Genotypes Carriers

eFigure 3. Crossed Drug/Gene Comparison of Dose Density

eFigure 4. Quality-Adjusted Life Years

eReferences

This supplementary material has been provided by the authors to give readers additional information about their work.

© 2024 Roncato R et al. *JAMA Network Open.*

eMethods. Detailed Methods

Study protocol and study design

PREPARE trial was an open-label, multicenter, controlled, cluster-randomized crossover implementation study. Full details of the PREPARE trial were previously published[2]. Eligible patients were determined according to inclusion and exclusion criteria described in detail by van der Wouden et al.[3]. All participants provided informed consent. The PREPARE trial adhered to the principles outlined in the 1975 Declaration of Helsinki (revised 1983) and was granted ethical approval by the local ethics committees. According to PREPARE randomization countries were block randomized as clusters to start with either genotype-informed drug prescribing (intervention group) or standard clinical care (control group). After 19 months, countries crossed over to the other group.

PREPARE trial was carried out in seven European countries with no restrictions regarding the choice of index drug prescription triggering the patient's enrollment. However, each country presented a specific clinical orientation. Between March 2017 and July 2020, in Italy patients were enrolled in the PREPARE trial under the coordination of CRO-Aviano Hospital, with the inclusion of 20 index drugs out of the 42 listed in the study protocol (i.e., acenocoumarol, atorvastatin, capecitabine, carbamazepine, clomipramine, clopidogrel, codeine, flecainide, fluorouracil, irinotecan, metoprolol, oxycodone, paroxetine, propafenone, simvastatin, tacrolimus, tamoxifen, tramadol, venlafaxine, and warfarin). Italian clinical centers were the only centers which were solely focused on oncology; therefore, they enrolled most patients treated with fluoropyrimidines (FP, capecitabine and systemic 5-fluorouracil) and irinotecan (IRI).

This secondary analysis includes patients enrolled in PREPARE across three participating clinical centers in Italy (Centro di Riferimento Oncologico - Aviano, San Filippo Neri Hospital- Rome, Ca'Foncello Hospital - Treviso). Patients in this secondary analysis can be considered as treated and followed up according to a single country sequential study design: testing a standard treatment approach for a time block and then switching to a genotype-informed strategy (Figure 1). According to the randomization procedure of the PREPARE trial, patients enrolled within the Italian centers initiated the standard of care intervention (control arm) for a duration of 18 months (2017-03-07 to 2018-09-30), after which the implementation of pharmacogenetic-guided prescribing (intervention arm) commenced for a subsequent cohort of patients (2018-10-01 to 2020-07-01).

Genotyping and intervention

For all enrolled participants, either a blood or saliva sample was collected for genomic DNA extraction prior to initiating the index drug. All patients enrolled in the PREPARE trial were successfully genotyped for the entire genetic panel of 50 polymorphisms across 12 pharmacogenes with clinical relevance[1]. The genotyping was performed using KASP technology in conjunction with the standardized LGC Genomics SNPline platform 1. DNA samples from patients enrolled in the control arm were processed after the end of the enrollment period. For patients enrolled in the intervention arm, the pharmacogenetic report including both the patient's genotype of all variants in the PREPARE panel and the clinical recommendation according to the most recent version of the Dutch Pharmacogenetics Working Group (DPWG) guidelines[1] was delivered to the treating physician and the patient within a 3-day turnaround (faster than the 7 days requested by the PREPARE protocol). All patients received a Medication Safety Code card containing a QR code that stored the patient's encoded pharmacogenetic test results. Scanning the QR code led to a website providing relevant DPWG recommendations. The Medication Safety Code card could be used to guide dose and drug selection for the index drug or any subsequently prescribed drugs.
Patients were defined as carriers of an "actionable genotype" if they were treated with fluoropyrimidines (FP, either capecitabine or 5-fluorouracil) and presented at least one select genetic variant among *DPYD*2A*, *DPYD*13*, *DPYD c.2846A>T*, or *DPYD c.1236G>A* or were treated with irinotecan (IRI) and were carriers of at least two select genetic variants among *UGT1A1*28*, *UGT1A1*6*, and/or *UGT1A1*27*. For carriers of an actionable genotype enrolled in the intervention arm, physicians received a pharmacogenetics report with clinical recommendation according to the DPWG guidelines before treatment initiation. Adherence to the dosing advice was not obligatory and left to the discretion of the treating physician.

Clinical and demographic data collection

Personal characteristics and demographics were recorded at baseline, whereas information regarding toxic events, hospitalizations, costs, and clinical outcomes, including Life Quality Assessments, were recorded at up-to four time points: baseline (study inclusion), 4 and 12 weeks and 18 months after the beginning of treatment.

According to the PREPARE protocol, the minimum follow-up period for each patient was 12 weeks and treatment outcome data were collected by research personnel either in person (for inpatients) or by telephone (for outpatients).
The severity of the recorded adverse drug reactions (ADRs) recorded at 4 weeks, 12 weeks, and 18 months after the start of treatment was assessed by the physician, while causality was independently evaluated by two study researchers. A medical doctor was consulted in case of controversy. All collected data were entered into an electronic case report form (eCRF). A random 10% sample of severity and causality assessments was independently reassessed by trained assessors from Lareb (the Netherlands Pharmacovigilance Centre), who were blinded to the patients' study group allocation.

Outcomes

Direct healthcare costs reimbursed by the Italian healthcare system included hospitalization costs (ordinary recovery and emergency room access) and the costs of managing adverse drug reactions on an outpatient basis (including blood test analysis, instrumental examinations, additional drugs, outpatient visits, etc.). The cost of oncologic treatment was not included due to the variety of regimens involved (i.e. monoclonal antibodies). Economic data were retrieved systematically through business intelligence and data analytics systems acquiring and integrating the information from the various healthcare company production sources. The economic evaluation of each hospitalization included both diagnosis-related groups (DRGs) and hospital discharge cards (SDOs).
Utility values were planned to be collected at four scheduled points consistent with the PREPARE protocol: baseline (study inclusion), 4 weeks, 12 weeks, and 18 months after the beginning of treatment. Utility values served as the basis for calculating quality-adjusted life years (QALYs), reflecting the average utility score reported by patients across these intervals. These scores were derived from both the patient's overall assessment of quality of life on a scale from 1 (very bad) to 100 (excellent) and weighted responses to the quality-of-life questionnaire. Inclusion in the analysis was limited to patients who completed a minimum of two out of the four assessments, drawing from at least one specified data source, resulting in a total of 491 participants (control group n=281, intervention group n=210). The computation of QALYs involved multiplying the utility score by patient survival, which was calculated based on the follow-up period of 18 months for quality-of-life assessment, providing a quantifiable measure of clinical benefit.

To assess the intensity of treatment in patients with an actionable genotype, we first calculated the measured dose intensity for FP and/or IRI for each patient with an actionable genotype, followed by the planned dose intensity for the scheduled regimen. Measured dose intensity represents the total mg of FP and/or IRI delivered per unit of time to the patient, divided by the actual days of treatment, and is expressed as mg/day. This calculation accounts for dosage reductions, suspensions, and premature discontinuations. In contrast, the planned dose intensity is based on the standard dosage mg/m2 and duration for the scheduled regimen with FP and/or IRI. On the contrary the planned dose intensity for the scheduled regimen has been based on the setting and pathology (www.eviq.org.au). Dose density was evaluated as the ratio of the measured dose intensity to the planned dose intensity for the scheduled regimen, expressed as a percentage. Additional data required for this analysis were successfully collected for 32 out of 40 patients with actionable genotypes.

Statistical analysis

Data are presented as absolute frequencies and percentages or as medians and interquartile ranges (IQRs). Differences between categorical variables were assessed using chi-squared or Fisher's Exact test as appropriate, whereas differences between continuous variables were evaluated based on median values using the Mann-Whitney test. Logistic regression analysis was used to estimate crude and adjusted odds ratios (ORs) with the corresponding 95% confidence intervals (CIs). Due to the cost distribution, quantile regression was used to model the median and 75th percentile of total toxicity costs, adjusting for sex and age. For each percentile modeled, predicted adjusted costs were calculated. $P<0.05$ was considered to be significant. We chose to report costs at the 75th percentile and to highlight the highest quartile as a representation of cost distribution because the majority of patients have low costs, some close to zero, whereas a minority have exceptionally high management costs. Gatekeeping analysis was carried out only on carriers of an actionable genotype (gatekeeper 1 group). Only if the difference was statistically significant, the analysis was carried out also on the overall population (gatekeeper 2 group). QALYs and dose density were described as mean percentage and relative standard deviation. Overall survival (OS) was calculated from the index drug start to the date of death or last follow-up. OS data were analyzed by the Kaplan-Meier method, and differences between groups were assessed using the log-rank test. All statistical analyses were performed using STATA statistical software.

© 2024 Roncato R et al. *JAMA Network Open.*

eResults. Detailed Results

Demographic and clinical characteristics

Of the 1,232 patients enrolled at the Italian implementation site, 563 were included in this secondary analysis. Most patients (n= 444, 78.8%) were on treatment with a FP-based regimen, while 20.2% (114 patients) were treated with a combination regimen including both FP and IRI, and 1% (5 patients) with IRI alone. Treatment was mostly given as a combination regimen (79.7% versus 20.3% as a monotherapy) and in a metastatic setting (43.9% versus 26.3% in neoadjuvant and 22.6% in adjuvant). The most common tumor type was colorectal cancer (63.2%), followed by gastric and gastro-esophageal junction cancer (17.8%), pancreatic (9.8%), anal (4.3%), esophageal cancer (2.7%) and bile ducts cancer (2.3%) (eTable 1 in Supplement 2).
A total of 2,135 ADR of any grade were recorded during the follow-up period. The majority (n=1,723, 80.7%) were non-hematological. Notably, nausea/vomiting was the most prevalent (n=432 recorded events), followed by fatigue (n=352), diarrhea (n=316), peripheral sensory neuropathy (n=284), hand-foot syndrome (n=48), and cardiotoxicity (n=37). In contrast, 412 events (19.3%) were hematological toxicities, the most common being neutropenia (n=168), followed by leukopenia (n=115) and anemia (n=71).
The majority of toxic events (n=1,728 out of 2,135; 80.9%) were classified as being "possibly" or "probably" related to the index drug according to the LCAT. In addition, 15 toxic events had a "definite" association with the drug. Toxicities considered to be "unlikely" associated with the index drugs were less common, except for peripheral sensory neuropathy, which was primarily associated with platinum-based co-treatment. Hematological toxicities were most frequently "likely" associated with FP and/or IRI, with 378 related toxic events out of 412 (91.7%) versus 1,377 out of 1,738 (79.2%) non-hematological toxicities (eFigure 1 in the Supplement 1).

Comparison of hospitalization and costs between arms

Overall, the recorded hospitalization events were mainly "likely" related to the treatment (55.1% versus 44.9% related to the disease). A total of 96 hospitalization events related to toxicity management (both access to emergency room and ordinary recovery) were recorded, 55.2% of them in the control arm and 44.8% in the intervention arm. The median length of hospital stay was 6 days and did not significantly differ between the two arms (IQR 4-15 in control arm, IQR 2-11 in intervention arm, *P*=0.292).

The overall sum of toxicity management-related costs was €344,339 and similarly distributed between the control and intervention arms (€177,149 and €167,190, respectively). Hospitalization was the major cost item, accounting for 96.7% of the total costs (eFigure 1 in the Supplement 1). On average, the cost of hospitalization was €3,396, with a minimum of €23 and a maximum of €12,224. The average cost of an emergency room visit was €131, with a minimum of €23 and a maximum of €336. For ordinary hospitalization, the average cost was €4,452, with a minimum of €1,198 and a maximum of €12,224.

Intensity of treatment

An exploratory analysis of FP and IRI treatment intensity was performed in 32 patients with any actionable genotype by calculating the dose density measured over the entire course of treatment. To enable this evaluation, FP and IRI dosing data were collected. All changes in drug dosing after the start of treatment were recorded, as well as delays in the administration of subsequent cycles or premature discontinuation of treatment.
Patients in the control arm started treatment with between 79 and 104 percent of the full standard dose. In the study arm, all patients with an actionable genotype started treatment according to specific pharmacogenetic recommendations (Table 1). Patients enrolled in the control arm had an overall mean delay of 48 days (median 37 days), compared with 21 days in the intervention arm (median 16).

To provide an integrated picture of overall dose density, including a timeline with dose reductions, overall delays, and treatment interruptions for each patient, we designed a swimmer plot (eFigure2). Notably, early FP treatment interruptions, marked with a red cross in eFigure2A, are fewer in the intervention arm (1/12, 8.3%) compared to the control arm (4/8, 50.0%). Similarly, for IRI in eFigure2B, none of the 4 patients in the intervention arm (0%) discontinued early, compared to 1 out of 8 patients (12.5%) in the control arm. Regarding dose reductions (marked with a ladder with downward arrow), in the intervention arm, 4 out of 12 patients treated with FP (33.3%) required dose reductions, compared to 3 out of 8 patients (37.5%) in the control arm. For IRI, none of the 4 patients in the intervention arm (0%) experienced dose reductions, as compared to 6 out of 8 patients (75.0%) in the control arm. Despite the exploratory nature of the investigation, due to the low number of evaluable patients, an improved

treatment adherence in the intervention arm as compared to the control arm could be appreciated based on above reported parameters.

An additional analysis was performed on patients receiving a combination regimen including both FP and IRI (n=17 patients). A description of the results stratified by highly impacting variants such as *DPYD**2A or *UGT1A1**28 is summarized in the sunflower plot in eFigure 3B. Despite the number of patients in each strata is too low to draw any final conclusion, the data suggests a noteworthy effect of *DPYD**2A or *UGT1A1**28 on the dose density of IRI and FP, respectively. Anecdotally, one patient with *DPYD**1/*2A genotype in the control arm had an IRI dose density of 50%, compared to 83% for one patient with the *DPYD**1/*2A genotype in the intervention arm (eFigure 3B). For 6 patients with *UGT1A1**28/*28 genotype in the control arm FP dose density was 56%, compared to 70% for 4 patients with *UGT1A1**28/*28 genotype in the intervention arm (eFigure 3B).

© 2024 Roncato R et al. *JAMA Network Open.*

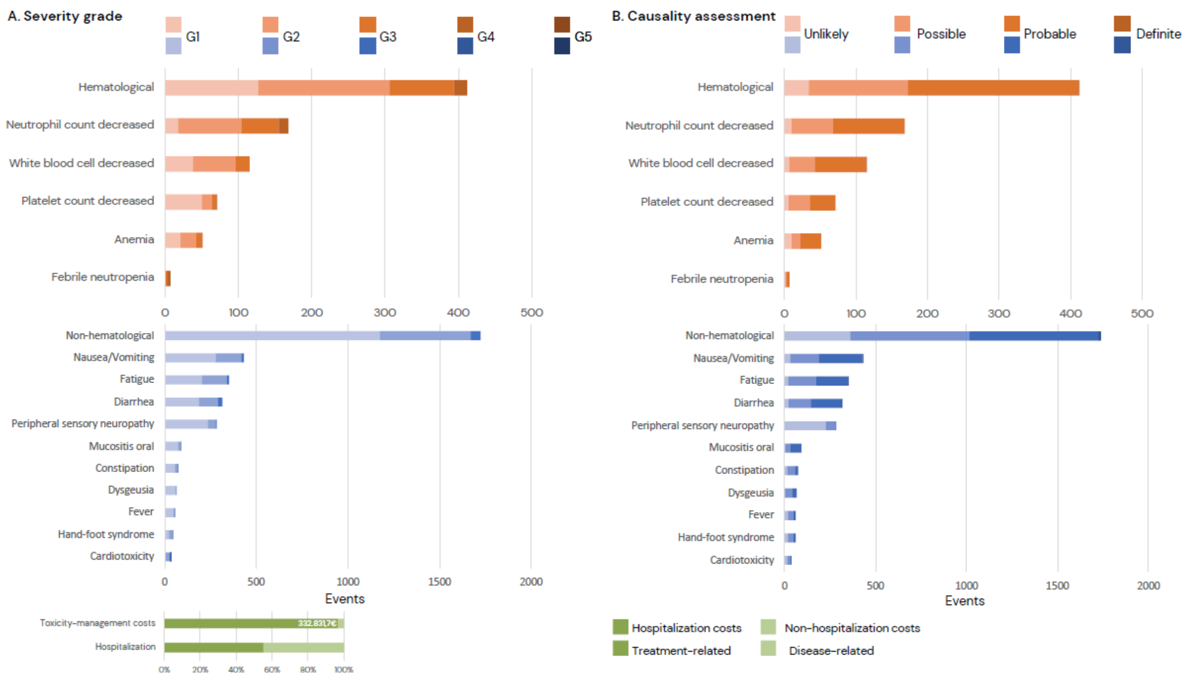

eFigure 1. Frequency of toxicity according to grade (A) and causality (B) and hospitalization costs. Impact of hospitalization costs on toxicity-management costs (A) and of treatment-related hospitalization events (B) on the total recorded among gastrointestinal cancer patients enrolled in the PREPARE trial in Italy.

© 2024 Roncato R et al. *JAMA Network Open.*

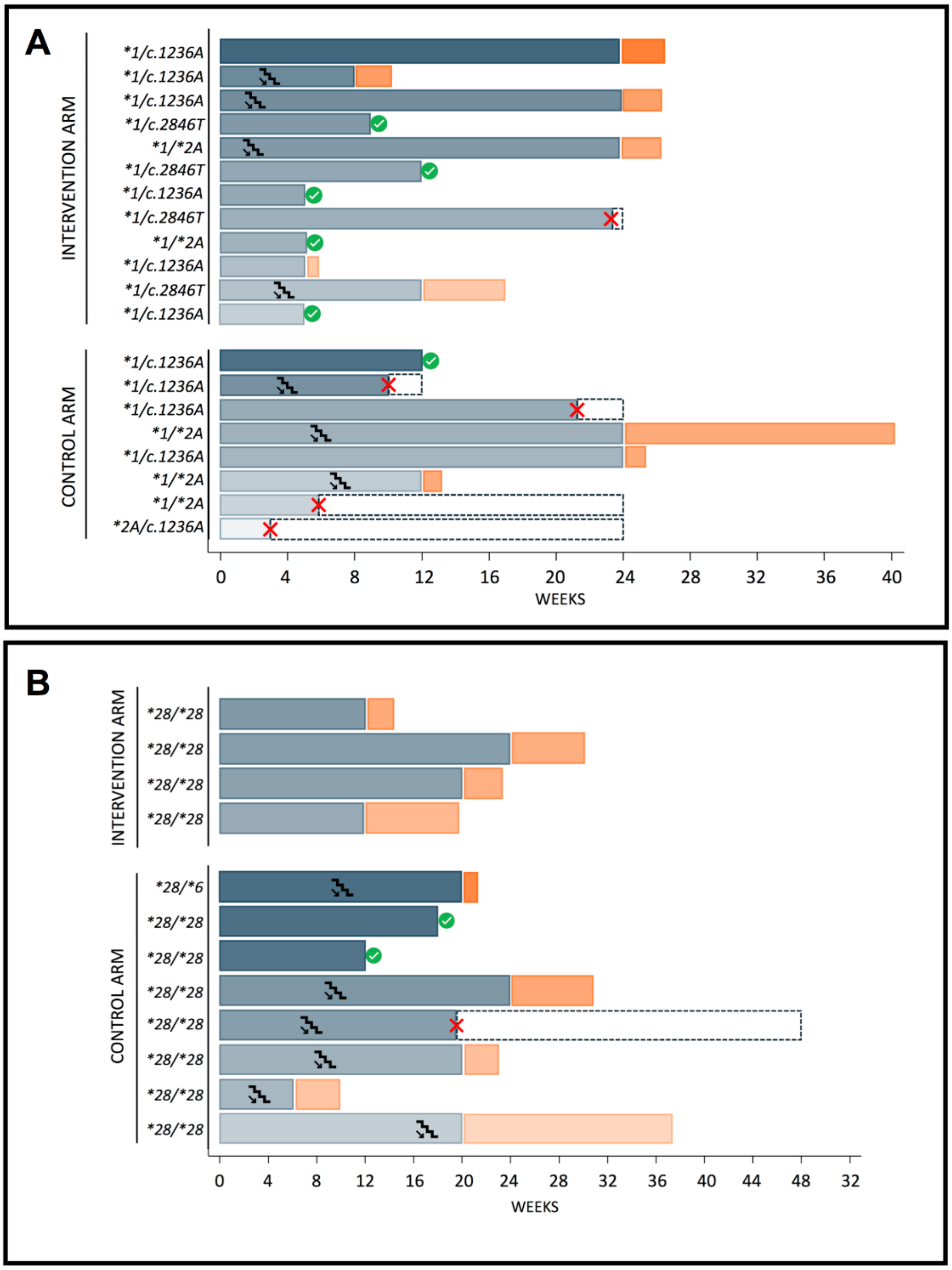

eFigure 2. Swimmer Plot: Case-by-Case Description of Treatment Adherence for Actionable Genotypes Carriers.

A) Fluoropyrimidines-related dose density, treatment delays, interruptions and dose reductions in patients with any actionable DPYD genotype. B) Irinotecan-related dose density, treatment delays, interruptions and dose reductions in patients with any actionable UGT1A1 genotype.

Each bar with a solid line represents the entire treatment duration for each patient. The blue bars indicate the duration of the drug administration within the planned treatment duration. The orange bars represent the additional duration of the drug administration beyond the planned treatment duration, due to delays in treatment. The shade of colors represents the percentage of overall dose density. The bars with the dashed outline represent the part of treatment not completed by the patient, due to treatment interruption.
The ladder with a downward arrow marks a dosage reduction.
The green checkmark marks a treatment course completed as planned.
The red cross indicates early treatment interruption.

© 2024 Roncato R et al. *JAMA Network Open.*

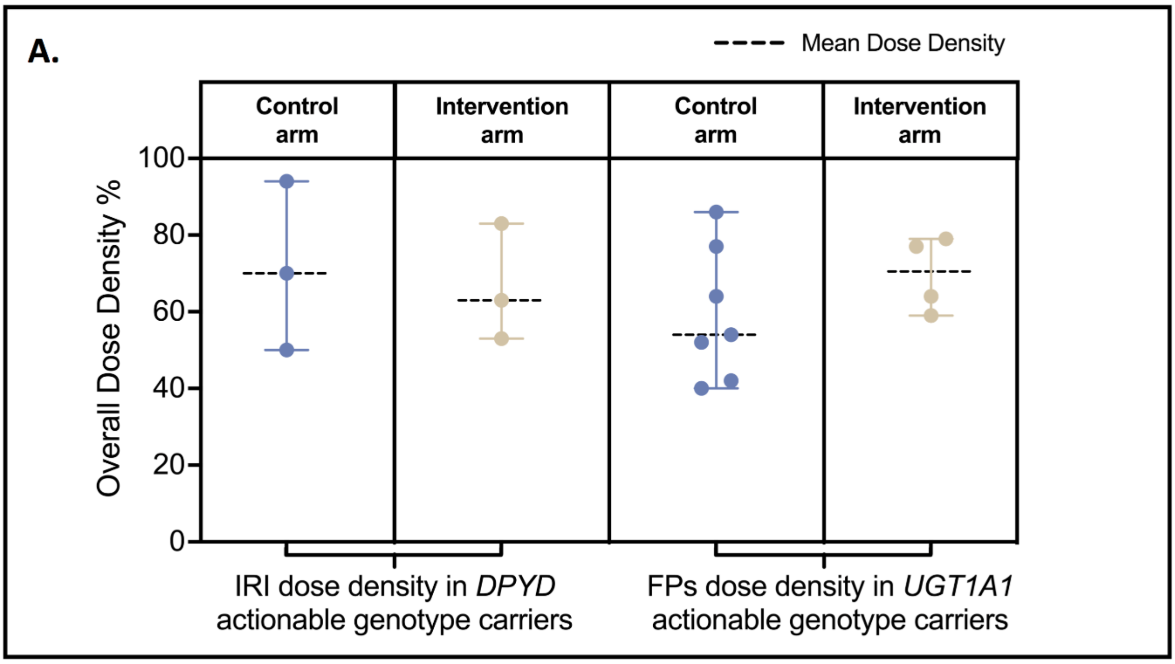

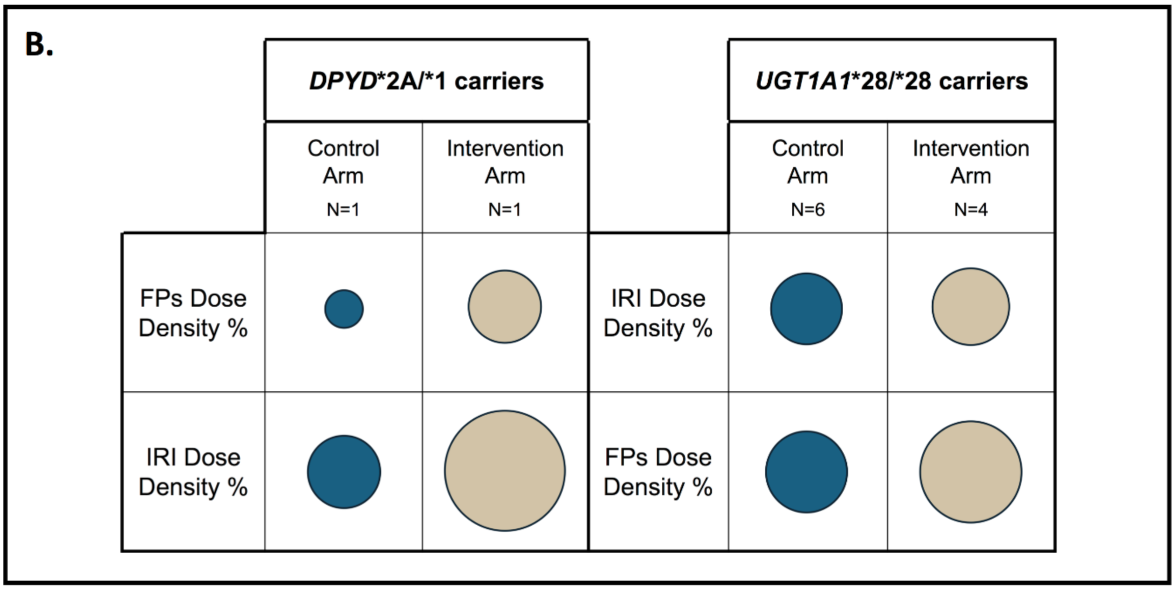

eFigure 3. Crossed drug/gene comparison of dose density.

A) Analysis performed on 17 patients receiving a combination regimen including both FP and IRI. Each dot represents a patient. The dose density of IRI in patients with a DPYD actionable genotype and of IRI in patients with a DPYD actionable genotype, in the control and intervention arms, respectively are reported.

B) Description of dose density for *DPYD**2A/*1 or *UGT1A1**28/*28 carriers receiving a combination of FP and IRI. Mean dose density percentage in patients in the control and intervention arms is represented with the size of the petals. Light blue represents the Control Arm, while light brown represents the Intervention Arm.

Abbreviation: N, number of patients; FP, fluoropyrimidines; IRI, irinotecan

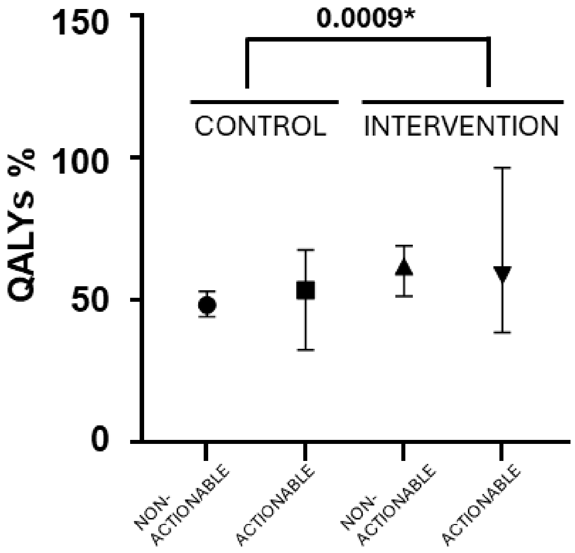

		**Patients**	**QALYs**		***P*-value***
		**N**	**Mean (%)**	**SD (%)**	
Control Arm	Non-actionable	259	53	26	Ref
	Actionable	22	53	27	1.000
Intervention Arm	Non-actionable	194	61	29	**0.006**
	Actionable	16	66	33	0.377

eFigure 4. Quality-Adjusted Life Years
Analysis of Quality-Adjusted Life Years in a subset of 491 patient between the two arms (281 in the control arm and 210 in the intervention arm).
Abbreviations: QALYs, Quality-Adjusted Life Years; N, number of patients; SD, standard deviation. The median value is highlighted in the graph while the error bars describe 95%CI.
**P*-value by Mann-Whitney test

© 2024 Roncato R et al. *JAMA Network Open.*

eReferences

1. Lunenburg CATC, van der Wouden CH, Nijenhuis M, et al. Dutch Pharmacogenetics Working Group (DPWG) guideline for the gene-drug interaction of DPYD and fluoropyrimidines. *Eur J Hum Genet*. 2020;28(4):508-517. doi:10.1038/s41431-019-0540-0

2. Swen JJ, van der Wouden CH, Manson LE, et al. A 12-gene pharmacogenetic panel to prevent adverse drug reactions: an open-label, multicentre, controlled, cluster-randomised crossover implementation study. Lancet. 2023;401(10374):347-356. doi:10.1016/S0140-6736(22)01841-4

3. van der Wouden CH, Cambon-Thomsen A, Cecchin E, et al. Implementing Pharmacogenomics in Europe: Design and Implementation Strategy of the Ubiquitous Pharmacogenomics Consortium. *Clin Pharmacol Ther*. 2017;101(3):341-358. doi:10.1002/cpt.602

© 2024 Roncato R et al. *JAMA Network Open.*
