# Supplementary material for: Clinical Benefits and Utility of Pretherapeutic DPYD and UGT1A1 Testing in Gastrointestinal Cancer: A Secondary Analysis of the PREPARE Randomized Clinical Trial
Source: JAMA Netw Open. 2024 Dec 6;7(12):e2449441. doi: 10.1001/jamanetworkopen.2024.49441 (PMC11624585; doi:10.1001/jamanetworkopen.2024.49441)
Supplement: Supplement 4. — Data Sharing Statement [file jamanetwopen-e2449441-s004.pdf]

Data Sharing Statement

Roncato. Clinical Benefits and Utility of Pretherapeutic DPYD and UGT1A1 Testing in Gastrointestinal Cancer. *JAMA Netw Open*. Published December 06, 2024. doi:10.1001/jamanetworkopen.2024.49441

Data
Additional Information: PREemptive Pharmacogenomic Testing for Preventing Adverse Drug REactions (PREPARE), https://clinicaltrials.gov/study/NCT03093818 NCT03093818
Data available: No

Additional Information
Explanation for why data not available: The original trial report includes a statement on data sharing reporting "A complete deidentified dataset will be made accessible, together with a data dictionary, for a minimum of 5 years. Requests for access to the data can be made by sending an email together with a research plan to the corresponding author and will be evaluated by and require authorisation from the Ubiquitous Pharmacogenomics Consortium executive board."
